# Identification of key regulatory genes connected to NF-κB family of proteins in visceral adipose tissues using gene expression and weighted protein interaction network

**DOI:** 10.1371/journal.pone.0214337

**Published:** 2019-04-23

**Authors:** Jamal S. M. Sabir, Abdelfatteh El Omri, Noor A. Shaik, Babajan Banaganapalli, Majed A. Al-Shaeri, Naser A. Alkenani, Nahid H. Hajrah, Zuhier A. Awan, Houda Zrelli, Ramu Elango, Muhummadh Khan

**Affiliations:** 1 Center of Excellence in Bionanoscience Research, King Abdulaziz University, Jeddah, Saudi Arabia; 2 Genomics and Biotechnology Section and Research Group, Department of Biological Sciences, Faculty of Science, King abdulaziz University, Jeddah, Saudi Arabia; 3 Department of Genetic Medicine, Faculty of Medicine, King Abdulaziz University, Jeddah, Saudi Arabia; 4 Biology- Zoology Division, Department of Biological Sciences, Faculty of Science, King Abdulaziz University, Jeddah, Saudi Arabia; 5 Department of Clinical Biochemistry. Faculty of Medicine, King Abdulaziz University, Jeddah, Saudi Arabia; King Saud University, SAUDI ARABIA

## Abstract

Obesity is connected to the activation of chronic inflammatory pathways in both adipocytes and macrophages located in adipose tissues. The nuclear factor (NF)-κB is a central molecule involved in inflammatory pathways linked to the pathology of different complex metabolic disorders. Investigating the gene expression data in the adipose tissue would potentially unravel disease relevant gene interactions. The present study is aimed at creating a signature molecular network and at prioritizing the potential biomarkers interacting with NF-κB family of proteins in obesity using system biology approaches. The dataset GSE88837 associated with obesity was downloaded from Gene Expression Omnibus (GEO) database. Statistical analysis represented the differential expression of a total of 2650 genes in adipose tissues (p = <0.05). Using concepts like correlation, semantic similarity, and theoretical graph parameters we narrowed down genes to a network of 23 genes strongly connected with NF-κB family with higher significance. Functional enrichment analysis revealed 21 of 23 target genes of NF-κB were found to have a critical role in the pathophysiology of obesity. Interestingly, GEM and PPP1R13L were predicted as novel genes which may act as potential target or biomarkers of obesity as they occur with other 21 target genes with known obesity relationship. Our study concludes that NF-κB and prioritized target genes regulate the inflammation in adipose tissues through several molecular signaling pathways like NF-κB, PI3K-Akt, glucocorticoid receptor regulatory network, angiogenesis and cytokine pathways. This integrated system biology approaches can be applied for elucidating functional protein interaction networks of NF-κB protein family in different complex diseases. Our integrative and network-based approach for finding therapeutic targets in genomic data could accelerate the identification of novel drug targets for obesity.

## Introduction

Obesity is a complex, multi-factorial metabolic disorder caused by the complex inter talk between an individual’s physiology and genotype with the environment. It is characterized by the development of the chronic inflammation in different tissues including adipose tissue and liver, leading to fat mass accumulation and weight gain [1]. Penetration of the macrophages and increased levels in proinflammatory cytokines is observed in adipose tissues in obese condition. The increased expression of TNF-α marks the first indication of a proinflammatory cytokine release in adipose tissues of obese mice [2] [3]. The ubiquitous complication of obesity is faulty insulin signaling in these tissues. Insulin resistance links obesity with cardiovascular diseases, type 2 diabetes, osteoarthritis, hypertension, and different forms of cancer [4].

It is acknowledged that chronic sub-clinical inflammation plays a crucial role in the initiation and progression of metabolic diseases [5]. Consistent with its key role in directing inflammatory responses, several studies have implicated the transcription factor Nuclear Factor-κB (NF-κB) in the initiation and progression of metabolic diseases, thereby further supporting the critical role of inflammation mediated metabolic disorders. The NF-κB protein family consists of five members, including *REL*, *RELA*, *RELB*, *NFΚB1*, and *NFΚB2*. NF-κB family of transcription factors regulates the expression of genes implicated in many important physiological responses such as inflammation, proliferation, differentiation, cell adhesion and apoptosis [6, 7]. The NF-κB pathway is a chief regulator of inflammatory processes and is associated with insulin resistance and pancreatic β cell dysfunction in the metabolic syndrome [8]. The NF-κB pathway ties the inflammatory and metabolic responses together. NF-κB pathway, being a key player in inflammation may help as an entry point for better understanding the metabolic diseases [9]. NF-κB proteins are activated by proinflammatory cytokines, apoptotic mediators, metabolic stress and chemical agents [10]. Genome-wide association studies of gene expression in adipose tissue have shown extensive inflammatory gene networks associated with obesity [11].

Correlating genes with diseases is a major challenge in human health for understanding disease biology and therapy. Predicting novel protein-protein interactions by incorporating high throughput functional genomics data has become a key approach in interpreting the protein functions and understanding molecular functions the inside cell [12]. In general, an efficient approach to study the regulatory role of genes implicated in any complex metabolic disorder like obesity is to create a biological network of functionally related genes [13]. The advent of networks and systems biology has revolutionized the transcriptomic and genomic approach to biology and influenced every aspect of biological research. Network-based methods have turned out to be more powerful and informative in studying the disease mechanism [14] [15]. In the genomics era, high-throughput researches have generated huge biological networks of interacting molecules. These networks are depicted as nodes connected by edges in complex graphs [16] [17]. In this framework, the characterization of biological networks by graph topological properties have become popular for gaining insight into the global network structure for normal and disease conditions.

In the present research analysis, we focused on NF-κB proteins and their inflammatory inducers. We followed well established network biology concepts in systems biology to identify the genes connected to NF-κB proteins with valid distinction from lean to obese samples. We filtered genes in such a way; they have a strong correlation with NF-κB as targets or inducers. Systems biology highlights a remarkable impact in understanding the interaction between genes and their associated pathways at the genome level [18] [19]. Hence its methods have been adopted in this study to identify the key NF-κB regulatory molecules and their pathways associated with obesity [20]. We used gene correlation, semantic similarity and topological parameters based on graph theory for transcriptome data to identify biomarkers. Gene correlation is based on the notion that genes with similar expression patterns are more likely to interact with each other more often [21]. The semantic similarity is based on the fact that genes associated with phenotypically similar diseases are often functionally associated at the molecular level [22] [23]. Candidate gene signatures are identified by calculating functional association between given genes and the known disease genes [24]. To gain insight into the organization and structure of the complex protein interaction network, we used topological parameters like degree, betweenness centrality [25]. It computes and represents nodes, edges, heterogeneity, and connected components. In this study, visceral adipose tissue gene expression datasets from lean female and obese female adolescents were collected from GEO database and analyzed. Statistical and knowledge based systemic investigations of high throughput data were considered to create a signature molecular network to identify candidate genes connected to the NF-κB family of proteins in obesity. We implemented a reliable integrated network-based method for identification of key signatures and their pathways implicated in the pathogenesis and to elucidate protein-protein interactions of the NF-κB proteins and obesity. Our biological network-based investigation will provide the novel association with potential biological insights and support future translational research on NF-κB proteins and obesity.

## Materials and methods

### Collection of datasets

**Fig 1:**
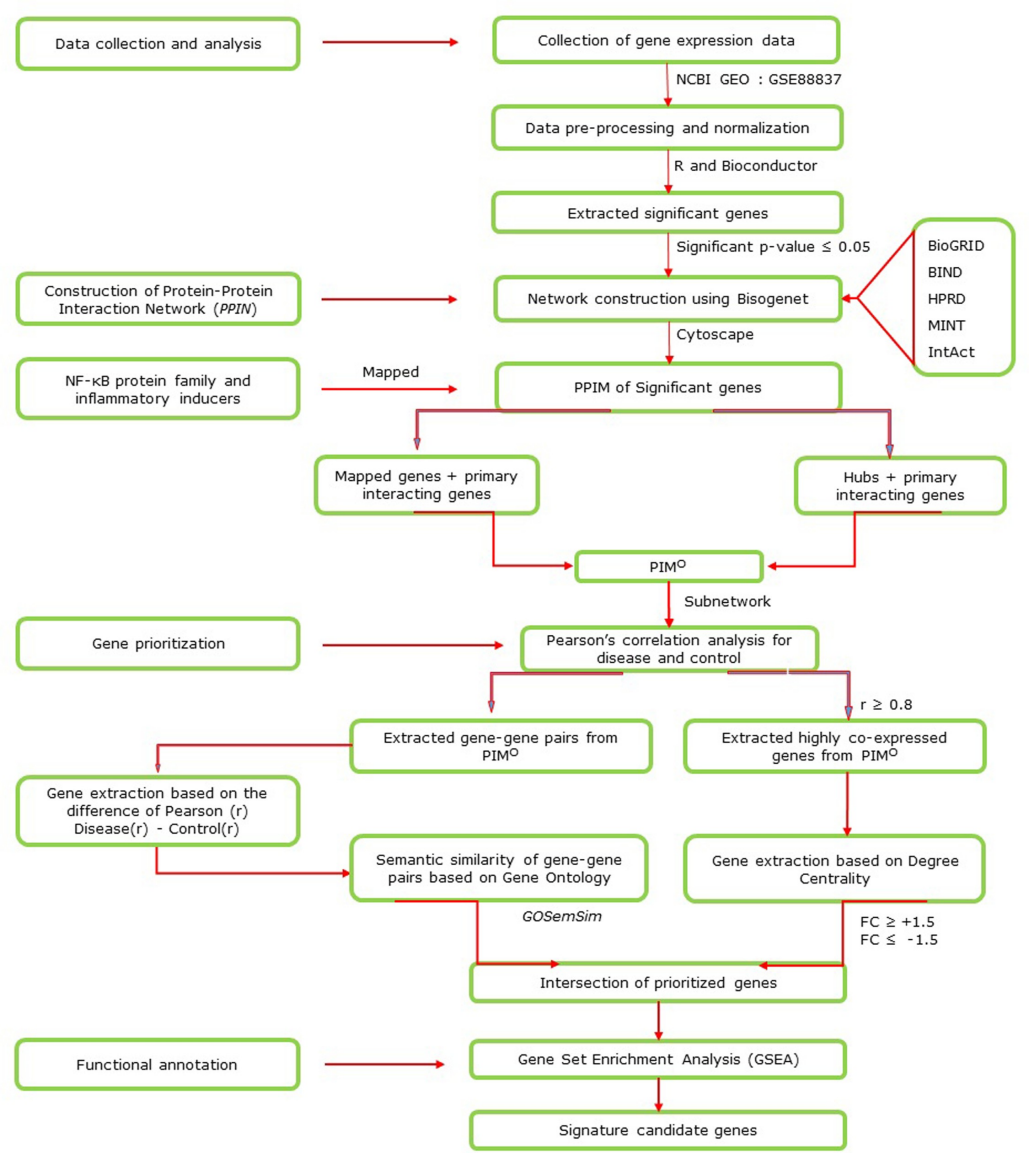
The overall work design of our pipeline. Present research analysis for exploring candidate genes from expression profiles.

The gene expression dataset GSE88837 associated with obesity was downloaded from Gene Expression Omnibus (GEO) database (www.ncbi.nlm.nih.gov/geo) [26]. The selected dataset GSE88837 was generated using the platform GPL570: Affymetrix Human Genome U133 Plus 2.0 Array. The dataset covers expression profiles of lean and obese adolescent females from visceral adipose tissue. The expression profiles consist of 14 samples from lean female adolescents (BMI < 25) and 16 samples from obese female adolescents (BMI > 25). The detailed sample information is given in the **S1 Table**. The overall work design used in the research investigations is shown in Fig 1.

### Expression data pre-processing and normalization

Expression level data analysis of the samples was performed using R packages [27] [28]. In order to standardize and reduce the technical noise in the probe level data, all the samples in the CEL file were loaded into *Affy* package, and raw signal values of each probe sets were normalized by baseline to a median of all samples using Robust Multiarray Average (RMA) algorithm. This algorithm normalizes the raw signals by creating an expression matrix from the data which involves background correction and log2 transformation followed by quantile normalization [29]. Next, the normalized samples were experimentally classified into normal (control) and obese (disease) sets. Further, the identification of statistically significant differentially expressed genes (DEGs) between the normal and obese samples was performed using unpaired t-statistic. The Benjamini and Hochberg’s false discovery rate with *p* value ≤ 0.05 was applied to identify the statistically significant list of DEGs.

### Construction of protein-protein interaction map

We used Bisogenet, a cytoscape plugin, to retrieve all the possible interactions among the DEGs obtained from the expression profiles [23]. Bisogenet retrieves the interaction among the significant genes from high-throughput experiments and literature data stored in Database of Interacting Proteins (DIP), Biomolecular Interaction Network Database (BIND), Human Protein Reference Database (HPRD), Biological General Repository for Interaction Datasets (BioGRID), The Molecular Interaction database (MINT) and IntAct databases [30]. Protein-Protein Interaction Map (PPIM) is a scale free network, based on a heterogeneous distribution of its node’s connectivity, in which several nodes have low connectivity, and a few nodes have a large number of connections [31]. In PPIM, nodes represent genes and edges are the physical or functional connection between the nodes. An edge built between two nodes points out protein binding, metabolic action or regulatory crosstalk among the nodes [32].

### Construction of subnetwork

Considering PPIM on a large scale, focusing down to every protein may be of less importance. The complex interactome PPIM was decomposed to a significant subnetwork of Significant Protein Interaction Network (S^PIN^) by following network biology concepts. From the PPIM, we extracted genes that belong to (a) hubs based on degree centrality (DC) and betweenness centrality (BC), (b) proteins of NF-κB family, and (c) inflammatory inducers of NF-κB proteins. To visualize and weigh the network centrality parameters (DC and BC) for each protein in the network, the PPIM developed from Bisogenet was standardized and incorporated into Cytoscape 3.2.1 [33]. The Cytoscape plugin Network Analyzer [25] was implemented to capture the local and global centrality parameters of the network [34].

### Identification of hub proteins

The degree of a node is the total number of edges that are linked to that particular node. Nodes with high DC in any network have large numbers of functional or interacting partners. In the constructed interactome PPIM, nodes with high degree connectivity correspond to essential genes. Moreover, several interacting functional partners in biological interactome are more likely to be involved in important biological pathways and cellular processes [32]. Implementing this concept, genes with high DC were chosen as hub proteins. Also, quite a lot of studies have proposed that genes associated with the disease have higher connectivity and cross-talks when compared to non-diseased genes which support the impact of hubs in the biological network [7, 35, 36]. Therefore, identifying hub molecules in the network can provide a better understanding of the pathogenesis of the disease. We adopted an approach, which has been formerly applied by Rakshit et al., [37] to identify the hubs. The DC cut-off threshold formula for choosing the hub protein is defined as:

Hubs=Avg(DC)+[2×SD(DC)] (Formula 1)

where *Avg* is the average degree centrality across all significant genes in the PPIM and *SD* represents their standard deviation [37].

As mentioned earlier, genes with high DC corresponds to essential genes, but DC does not measure the significance of a gene in the interactome on a global scale [38]. Hence, a global parameter BC was introduced to scale the properties of a gene at the whole interactome level. The BC of a node is the control of a node that exerts over the interactions of other nodes which are functionally relevant in the network. This centrality magnitude favors nodes that link dense networks, rather than nodes that are located inside the dense cluster [39]. BC is calculated using the formula:

$$BC\;\left(n\right)=\sum_{s\neq n\neq t}\left[\frac{\sigma_{st}\left(n\right)}{\sigma_{st}}\right]$$ (Formula 2)

where ‘s’ and ‘t’ are nodes in the network other than ‘$n’,\;\sigma_{st}$ represents the number of shortest paths from ‘s’ to ‘t’, and $\sigma_{st}\left(n\right)$ is the number of shortest paths from s to t that ‘n’ lies on [40]. Using the node betweenness distribution, genes positioned in the top 50% are scaled as hub genes.

#### Proteins of NF-κB family

The NF-κB family consists of five proteins, NFKB1, NFKB2, REL, RELA, and RELB. S2 Table represents the details of the proteins of the NF-κB family.

#### Inflammatory inducers of NF-κB proteins

We collected inflammatory inducers of NF-κB proteins from the database of NF-kB Transcription Factors (www.bu.edu/nf-kb/) maintained by Boston University. Twenty eight inflammatory molecules were reported in the database shown in S3 Table. For the ease of exploration, all NF-κB family proteins, hubs and inflammatory inducers of NF-κB proteins together, we use the term *HIN*^*NF*^. The genes of *HIN*^*NF*^ with their primary interacting partners were pulled out from the complex interactome PPIM to construct S^PIN^.

### Construction of weighted gene-gene correlation map

The gene-gene correlation map across the entire gene set in the S^PIN^ was generated using Pearson’s correlation algorithm. Correlation (r) between every pair of genes in the microarray data sets was ranked based on Pearson’s correlation coefficient (PCC). The PCC between pairs of genes is calculated using the formula mentioned in Formula 3.

$$PCC\;\left(r\right)=\frac{\sum_{i=1}^{n}(x_{i}-\underset{-}{x})\;\left(y_{i}-\underset{-}{y}\;\right)}{\sqrt{\sum_{i=1}^{n}(x_{i}-\underset{-}{x})²}\;\sqrt{\sum_{i=1}^{n}(y_{i}-\underset{-}{y}\;)²}}$$ (Formula 3)

where $\underset{-}{x}$ and $\underset{-}{y}$are the sample mean of the expression values in control and diseased state of the two genes, respectively.

#### Gene prioritization algorithm

Prioritizing the most promising candidate genes in the interactome is a challenging and time consuming task. Thus, we implemented the following filtering measures centered on biological insights to prioritize the genes in the interactome of S^PIN^.

### Pearson correlation coefficient between gene pairs in S^PIN^

In this approach, PCC between the gene-gene pairs in S^PIN^ was generated for both disease and control group separately. Next, the difference of PCC (*D*_*PCC*_) between gene pairs of disease and control groups was calculated using the following formula [37]:

$$D_{PCC}=Disease\left(r\right)-Control\left(r\right)$$ (Formula 4)

where $Disease\left(r\right)$ is the PCC of disease samples and Control(r) represents PCC of control samples. Higher $D_{PCC}$ score implies distinct variation in gene’s interaction from control to disease condition. To increase the stringency, gene pairs with absolute difference score, $D_{PCC}$ ≥ 1 were screened for further analysis.

### Functional similarity between gene pairs

Generally, genes having an association in phenotypically similar diseases are often functionally associated at the molecular level [20]. The functional likeness between two genes is measured using encoded evidence in the Gene Ontology (GO) hierarchies. In the current analysis, we applied Wang’s measure of similarity [41] to the molecular function (MF) hierarchy. This measure determines the semantic similarity of two genes based on the locations of GO terms in the graph and their semantic relations with their ancestor terms. The score of semantic similarity between the terms ranges between 0 and 1. A higher score implies a strong functional association between the genes. The semantic similarity between gene pairs is calculated using the following formula:

$$S_{GO}\left(X,Y\right)=\frac{\;\sum_{t\in T_{X}\cap T_{Y}}\left(S_{X}\left(t\right)+S_{Y}\left(t\right)\right)}{\sum_{t\in T_{X}}S_{X}\left(t\right)+\;\sum_{t\in T_{Y}}S_{Y}\left(t\right)}$$ (Formula 5)

where $T_{X}$ represents the set of all its ancestor terms including term X itself and $S_{X}\left(t\right)$ denotes the contribution of a term *t* ∈ $T_{X}$ to the semantics of X based on the relative locations of t and X in the graph. Since a gene can be annotated by many GO terms, we used Best-Match Average (BMA) method which combine semantic similarity scores of several GO terms and calculates the average of all maximum similarities on each row and column. Based on this concept, we employed R package, *GoSemSim* [42] to measure the semantic similarity between gene pairs with score $D_{PCC}$≥ 1. Next, gene pairs were filtered on semantic score ≥ 0.5 as higher score implies stronger association.

### Co-expression analysis of genes in S^PIN^

In this method, we focused on the gene pairs whose expression is significantly correlated by selecting the r value cut-off, r ≥ 0.8, as higher r value implies stronger association [37]. Next, from the correlation matrix, genes in S^PIN^ showing higher correlation, r ≥ 0.8 were screened for both disease and control groups. Further, DC difference of S^PIN^ (${DC}_{S^{PIN}}$) between disease and control groups was calculated as follows:

$$DC_{S^{PIN}}=DC_{Disease}-DC_{Control}$$ (Formula 6)

where ${DC}_{Disease}$ and $DC_{Control}$ represents degree of the node in disease and control group respectively. The DC difference of a node represents its gain or loss of functional partners. Positive value of ${DC}_{S^{PIN}}$ denotes loss of its interacting genes and negative value denotes gain of interacting genes from healthy to diseased state. Next, the genes in S^PIN^ with FC of 1.5 (-1.5 ≥ FC ≥ +1.5) were mapped to genes of ${DC}_{S^{PIN}}$ to identify genes with distinct variation based on fold change. These filtered genes are used for downstream functional enrichment analysis.

### Gene set enrichment analysis (GSEA)

Genes do not interact in isolation, and unforeseen cross-talk may lead to dysregulated functions [43]. Hence, to comprehend the biological system, it demands the knowledge of the interconnectivity of genes in several processes and pathways that ascend from both physical and functional interactions. Such biological interactome can be developed by assessing the functional characteristics of the genes. Performing functional enrichment analysis on gene sets is a crucial step in understanding high-throughput biological data [44]. This approach substantiates that the genes involved in a biological experiment are functionally significant and helps to discover unintended interaction between the genes. Functional enrichment of the filtered genes was performed using ToppGene Suite [45].

### Tissue specific analysis

Tissue specific interactions were identified using GIANT (Genome-scale Integrated Analysis of gene Networks in Tissues). GIANT powers a gold standard tissue-specific analysis to elucidate roles of genes and expose changes in those roles across tissues [38]. GIANT identifies tissue-specific connections by combining diverse functional genomics data over 61400 experiments for 283 diverse tissues and cell types [46]. GIANT generates an interactive network for the queried genes with specific edge weight ranging from 0 to 1. Higher the score higher is the relationship confidence between the genes.

## Results

### Assessment of gene expression profiles

**Fig 2:**
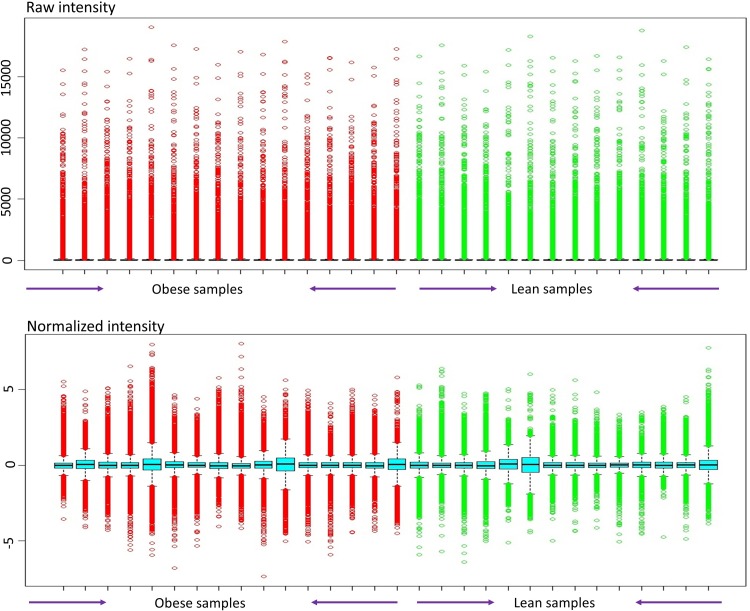
Gene expression data before and after normalization. The horizontal axis represents the samples, and the vertical axis represents the gene expression values.

High throughput experimental gene expression profiles of visceral adipose tissues collected from 14 lean female adolescents (BMI < 25) and 16 obese female adolescents (BMI > 25) were analyzed to pinpoint the dysregulations in key molecular signatures affected in adipose tissues. The raw expression profiles containing 54675 (probes) entities were normalized using the Robust Multiarray Average (RMA) algorithm, reduced to 22482 non-redundant data points. Further, we screened 2650 differentially expressed genes (DEGs) with a statistical significance of p value ≤ 0.05. The normalized datasets are represented as box plots to show the data distribution (**Fig 2).**

### Constructed protein-protein interaction map

A total of 2650 significant genes obtained from the expression analysis were queried in Bisogenet, a Cytoscape plugin, to create PPIM by retrieving all possible associations among genes. The PPIM was then stabilized by removing the outliers, self-loops and duplicated edges to assess the standardized topological characteristics for each gene. The plugin generated a complex PPIM, comprised of 2650 nodes (genes) and 169118 edges (interactions) with 63.82 average edge-node ratios. Further, Network Analyzer plugin was employed to calculate local (degree centrality) and global (betweenness centrality) parameters of the network. An overview of the top 10 significant genes based on the highest degree is presented in **Table 1**along with general centrality parameters.

**Table 1:** List of top 10 significant genes with the highest degree with their general centrality parameters obtained from network analysis.

Gene	BC	CC	DC
UBC	0.096	0.739	1640
PHF8	0.051	0.617	988
EGR1	0.019	0.567	639
CHD2	0.012	0.553	524
FOS	0.011	0.553	515
JUND	0.011	0.549	479
APP	0.010	0.575	707
EBF1	0.010	0.560	594
STAT3	0.009	0.549	493
IRF1	0.007	0.537	386

#BC = Betweenness Centrality, CC = Closeness Centrality, DC = Degree Centrality

### Significant protein interaction map (S^PIN^)

**Fig 3:**
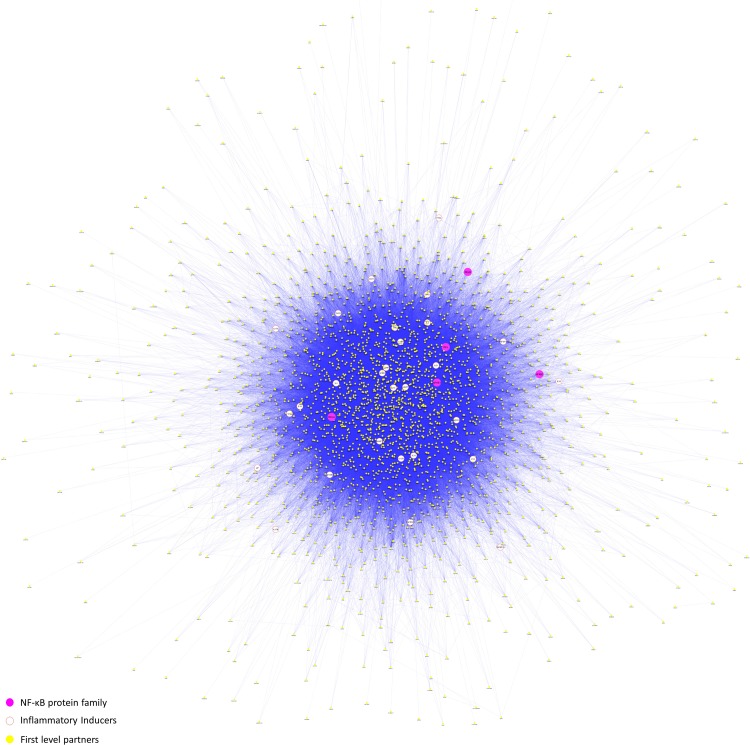
Significant protein interaction map. S^PIN^, developed from *HIN*^*NF*^ and their first level interacting partners.

The genes of PPIM were classified into hubs on the basis of topological parameters for the construction of a significant protein interaction network. Hubs are the key features as they indicate critical intersections among clusters in the network if removed the network will be disrupted [47]. The threshold cut-off for hubs and bottlenecks were specified based on the Formulas 1 and 2. Total of 28 genes was screened as inflammatory inducers of NF-κB proteins. Implementing this method, we selected 1261 hubs, 28 inflammatory inducers and 5 proteins of NF-κB family. **H**ubs, **I**nflammatory inducers and **N**F-κB protein family (***HIN***^***NF***^) were together comprised of 1277 genes (17 redundant genes, which comes from inducers and NFKB were removed). *HIN*^*NF*^ genes with their respective first level interacting partners were extracted from PPIM to build significant protein interaction map, S^PIN^ (Fig 3). In total 2525 S^PIN^ genes were selected for downstream analysis.

### Gene-gene correlation and semantic similarity of genes in S^PIN^

The expression matrix across 2525 genes in the S^PIN^ was created for both control and disease samples based on Pearson’s correlation algorithm. The algorithm generated PCC for 70225010 gene pairs from 2525 genes for both control and disease samples (Formula 3). A detailed parametric downstream analysis was performed on the 70225010 gene pairs to dissect most indispensable signatures from the interactome. For this purpose, we followed filtering methods centered on biological insights for gene prioritization.

### PCC between gene pairs in S^PIN^

From the correlation map of 70225010 gene pairs obtained, we developed an in-house algorithm to screen and match 169118 gene pairs that are present in S^PIN^. Then, the difference of *PCC* ($D_{PCC}$) between these extracted gene pairs was calculated from disease and control samples based on the Formula 4. To narrow down the most crucial gene pairs, we considered a higher absolute difference score of ≥ 1 for${\;D}_{PCC}$. We have chosen a higher score of $D_{PCC}$ as it illustrates the significant variation or instability in interactions among the gene pairs from control to disease state. We obtained 2424 gene pairs comprising of 1423 genes (This include some of the inducers and NFkB and 1261 genes in the input) with ${\;D}_{PCC}\;$greater than or equal to one. **Table 2**shows list of top 10 gene pairs with higher absolute *D*_*PCC*_ score (S4 Table).

**Table 2:** An overview of the top 10 significant genes gene pairs with the highest absolute *D*_*PCC*_ score.

Gene pairs		Pearson's Correlation Coefficient (r)		
Term 1	Term 2	Disease(r)	Control(r)	Abs (*D*_*PCC*_)
TRIAP1	MAN2B2	-0.8449	0.790618	1.63552058
DCAF8	NFATC2IP	-0.81999	0.7863	1.6062919
P4HA1	PFDN4	0.800708	-0.79465	1.59535325
XRCC5	EPHX2	-0.74657	0.793173	1.53974146
PCNA	RUFY3	-0.74982	0.779376	1.52919467
PSMB2	SPG20	-0.72599	0.801969	1.5279635
LRP8	THBS3	-0.80379	0.715712	1.51949733
MCM3	TOP2A	0.711379	-0.79084	1.5022185
SP3	RCC2	-0.72834	0.75025	1.4785944
CCT3	MAN2B2	-0.7553	0.708207	1.46350675

### Semantic similarity between gene pairs

**Fig 4:**
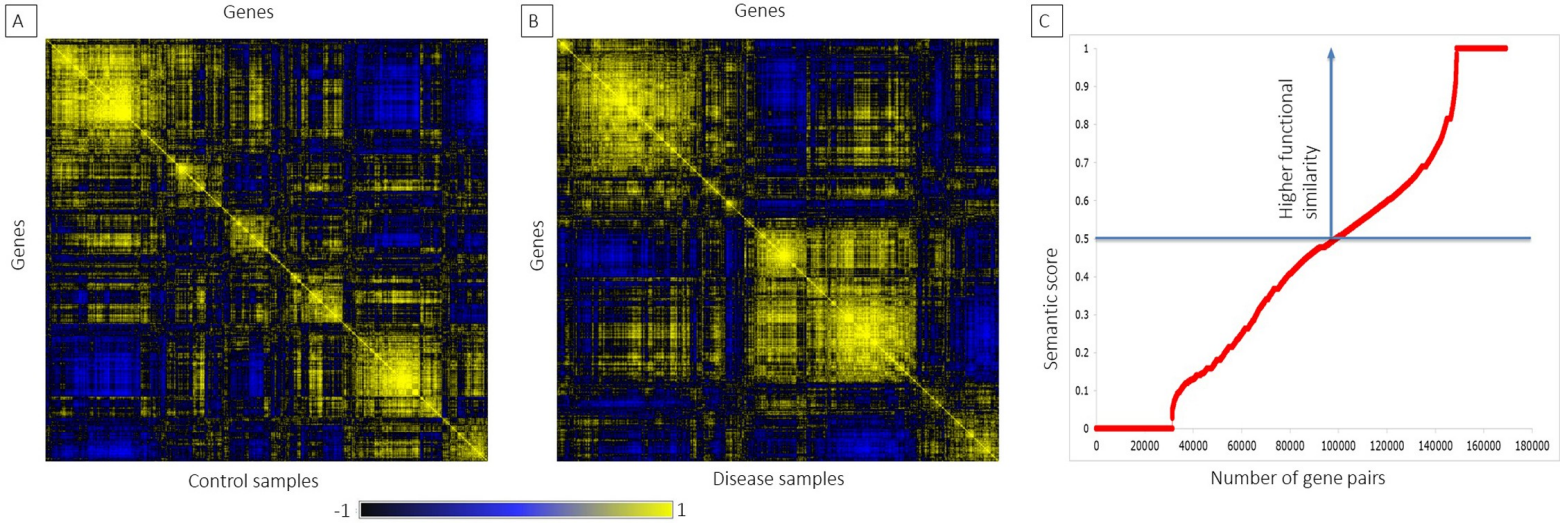
Representation of gene-gene correlation plot and semantic similarity graph. The correlation plots illustrate significant variations in gene expression among the gene-gene pairs in the control and disease samples. A). Gene-gene correlation of normal samples (control), B). Gene-gene correlation of obese samples (disease), C). The graph depicts semantic similarity between all pairs of genes and the blue arrow represents gene pairs with higher functional similarity.

The gene pairs with higher $D_{PCC}$ score was screened from the S^PIN^, and we applied Wang’s measure of semantic similarity [41]. We developed a semantic similarity score for 2424 gene pairs with $D_{PCC}$ ≥ 1 by exploiting *GoSemSim* package in R. Next, we filtered 992 gene pairs, comprising 809 genes, with strong functional association based on higher a semantic score of ≥ 0.5, as shown in the Fig 4A, 4B and 4C. Table 3 depicts a list of top 10 gene pairs with the highest semantic score (*S*_*GO*_) and absolute *D*_*PCC*_ score (S5 Table).

**Table 3:** The table represents the top 10 gene pairs with the higher semantic score (S_GO_) with their absolute *D*_*PCC*_ score.

Gene pairs		Score	
Term 1	Term 2	Abs (*D*_*PCC*_)	S_*GO*_
MCM3	TOP2A	1.502219	1
XRCC5	RBM17	1.451234	1
TEX10	PELP1	1.416311	1
NEDD4L	PRICKLE1	1.413676	1
MCM3	HLA-C	1.412932	1
COL4A1	FZR1	1.39018	1
TOP2A	BIK	1.38565	1
ESRRG	RPRD1A	1.379133	1
RAB35	COG6	1.364074	1
HDAC1	RBM17	1.356559	1

### Co-expression analysis of S^PIN^

**Fig 5:**
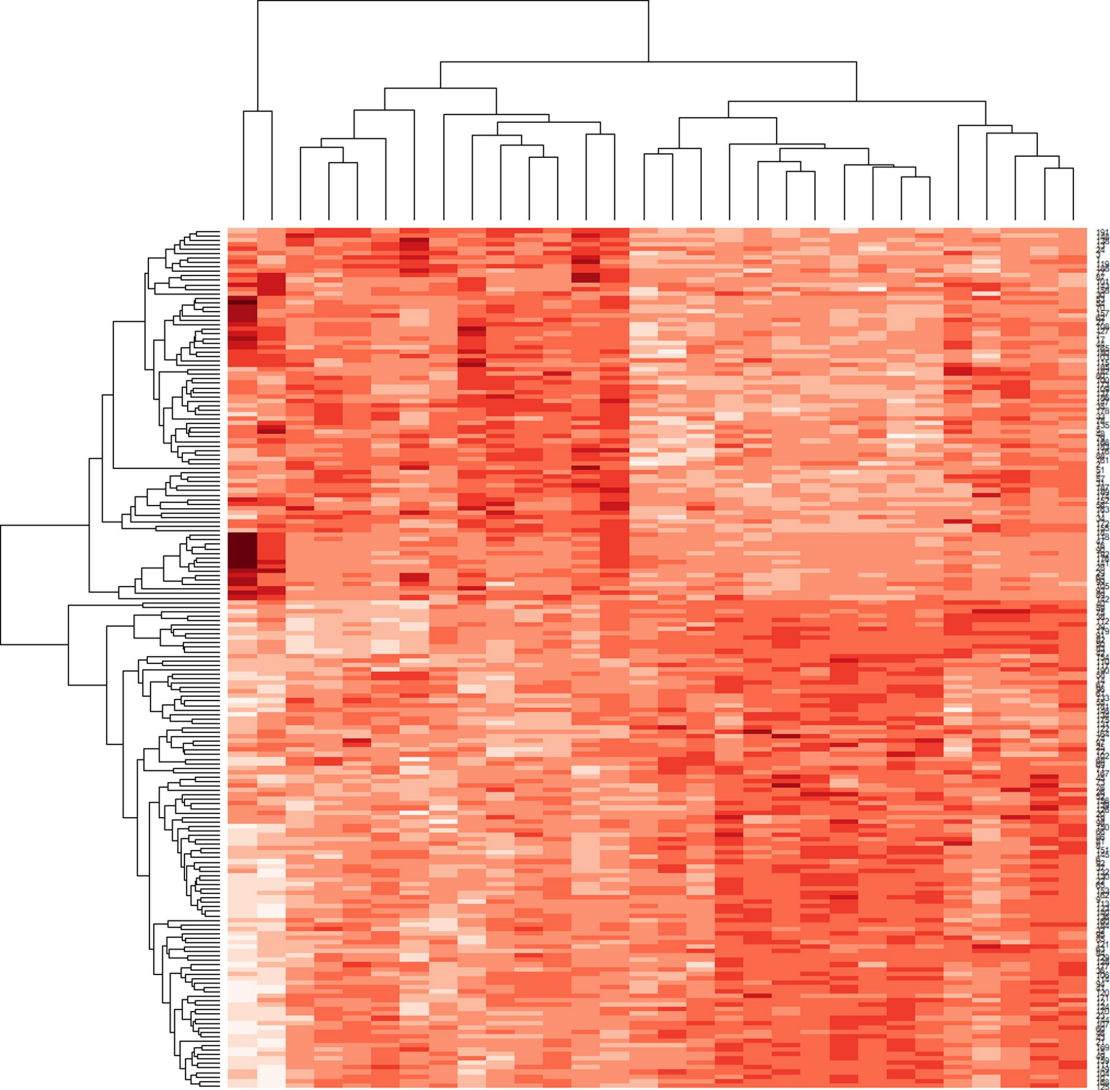
Expression pattern of the filtered genes from *HIN*^*NF*^ and primary functional partners contributing to total of 193 genes. The gene expression pattern analysis clearly depicts variation in expression in disease and control samples.

In this approach, gene pairs were selected based on the following established concepts. i) The expression level of genes with high positive correlation. ii) Genes with similar expression patterns are more likely to interact with each other. Thus, gene pairs with higher correlation were screened for both disease and control sample sets. Gene pairs with r ≥ 0.8 from correlation map were chosen, as higher r score represents stronger association [37]. Next, the degree (number of functional partners) of 809 genes obtained from the aforementioned method with higher correlation was extracted using an in-house script. Again, DC difference of these genes was calculated using the formula in Formula 6 (section 2.6) which denotes alterations in connectivity of the gene from control to the diseased state. Alterations of gene connectivity in biological networks are linked to substantial phenotypic changes [48]. Positive value of ${DC}_{S^{PIN}}$ for a gene indicates gain of connectivity or functional partners and the negative value represents the loss of connectivity or functional partners. We applied a FC threshold of ±1.5 (-1.5 ≥ FC ≥ +1.5) to filter deregulated genes based on FC. We obtained total of 193 genes by this filtering approach, in which 112 genes with loss of functional partners and 74 gene with gain of functional partners (S6 Table). The node degree of the remaining 7 genes did not change in between controls and disease samples. We developed heat maps for these genes from their expression pattern to visualize and interpret more comprehensively (Fig 5).

### Gene set enrichment analysis

**Fig 6:**
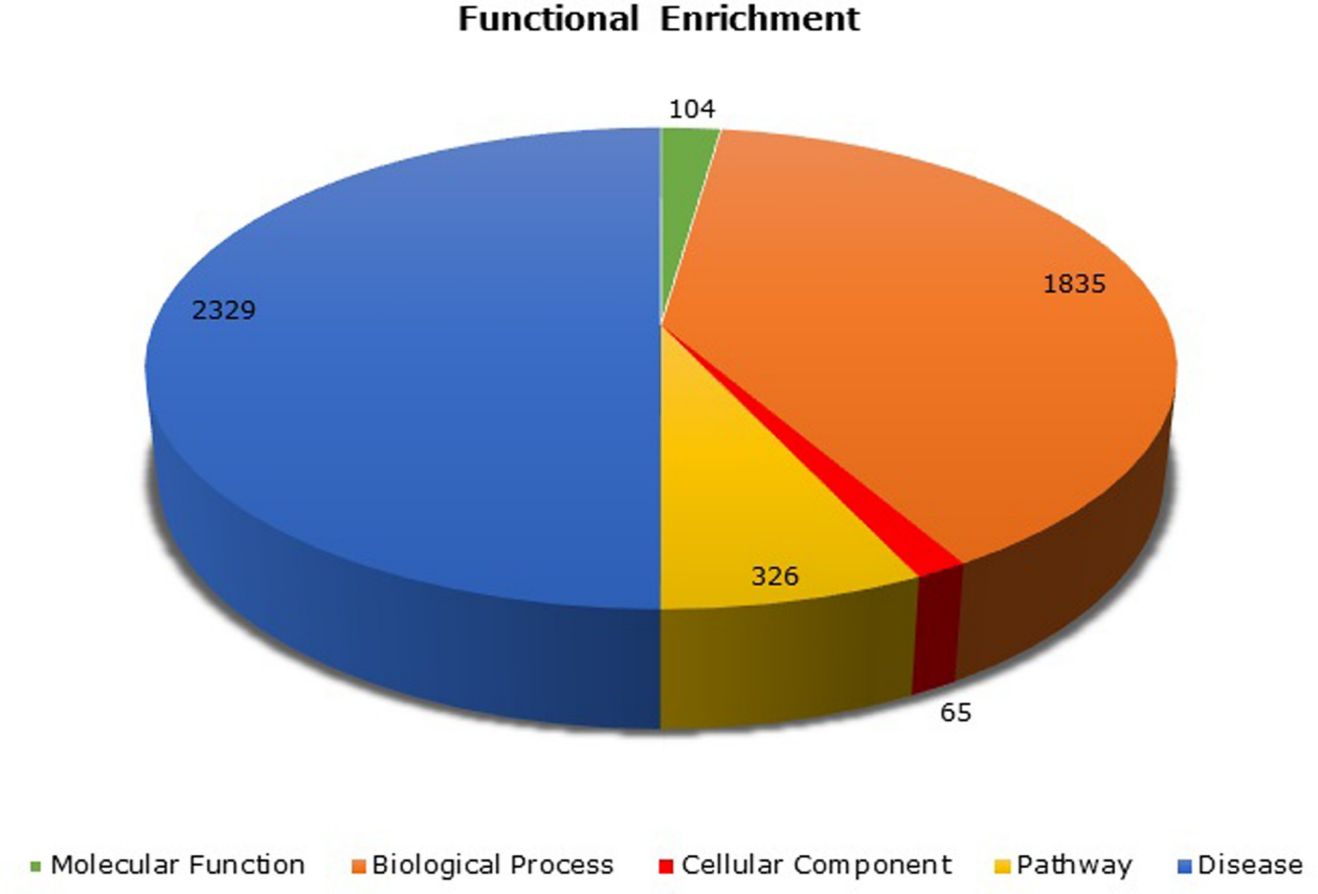
Gene enrichment. The overall view of gene set enrichment analysis on filtered genes.

**Fig 7:**
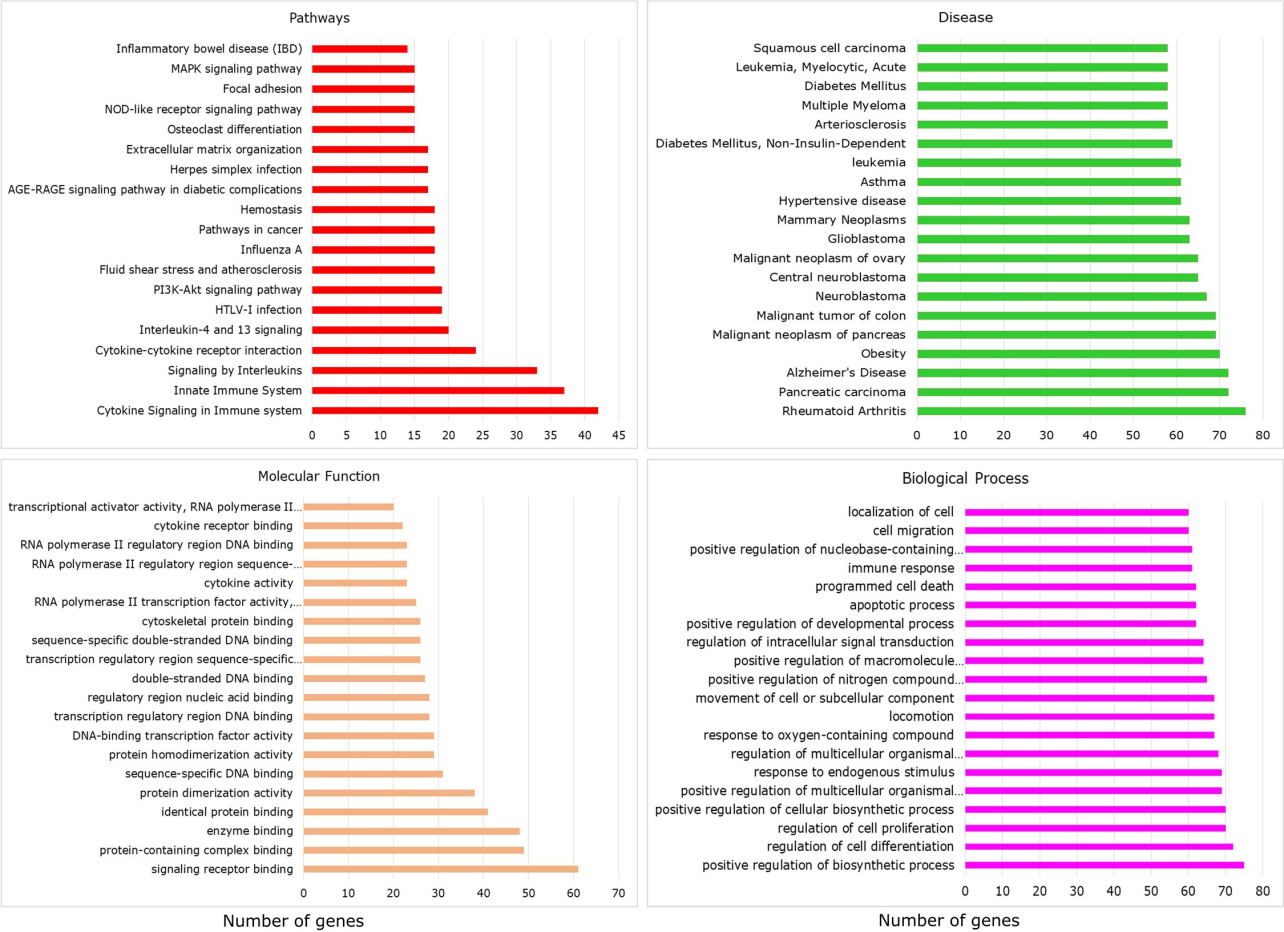
Top 20 terms of gene set enrichment analysis for the pathways, disease, molecular function and biological process. Genes enriched are more closed to inflammatory diseases and pathways.

To assess the functional and biological significance of the filtered 193 genes, we used the ToppGene functional annotation suite. Functional enrichment implemented using ToppGene represented annotated genes by exploring databases like KEGG, Reactome, Pathway Interaction Database, DisGeNET and GO databases with a p-value threshold ≤ 0.05. The gene sets were enriched with 1835 biological process (BP), 104 molecular function (MF), 65 Cellular Component (CC), 326 Pathways and 2329 diseases. The pie chart of enrichment analysis is shown in Fig 6. Enrichment analysis represented about 70 genes in obesity. Thus, the occurrence of known obesity susceptible genes in the prioritized list substantiates the relevance of our approach. Thus, the occurrence of known obesity susceptible genes in the prioritized list substantiates the relevance of our approach. Again, most of the genes in the enriched network represented their association in diseases like *Rheumatoid arthritis*, *Pancreatic carcinoma*, *Alzheimer's disease*, *Diabetes Mellitus* and *Hypertensive disease*. The biological process associated with these genes included *immune response*, *response to lipid*, *MAPK cascade*, *NF-kappa B signaling*, *inflammatory response*, *response to nutrient levels*, *response to insulin*, *regulation of peptidyl-tyrosine phosphorylation* and *response to glucose*. Genes were also enriched in pathways like *Cytokine-cytokine receptor interaction*, *PI3K-Akt signaling pathway*, *Hemostasis*, *NOD-like receptor signaling*, *Focal adhesion*, *NOD-like receptor signaling*, *TNF signaling*, *NF-kappa B signaling*, *Glucocorticoid receptor regulatory network*, *Adipocytokine signaling*, and *Toll-like receptor signaling*. The enriched pathways, diseases, and biological process show a high correlation with obesity. The list of top 20 functional annotations with the respective gene count is depicted in Fig 7.

**Fig 8:**
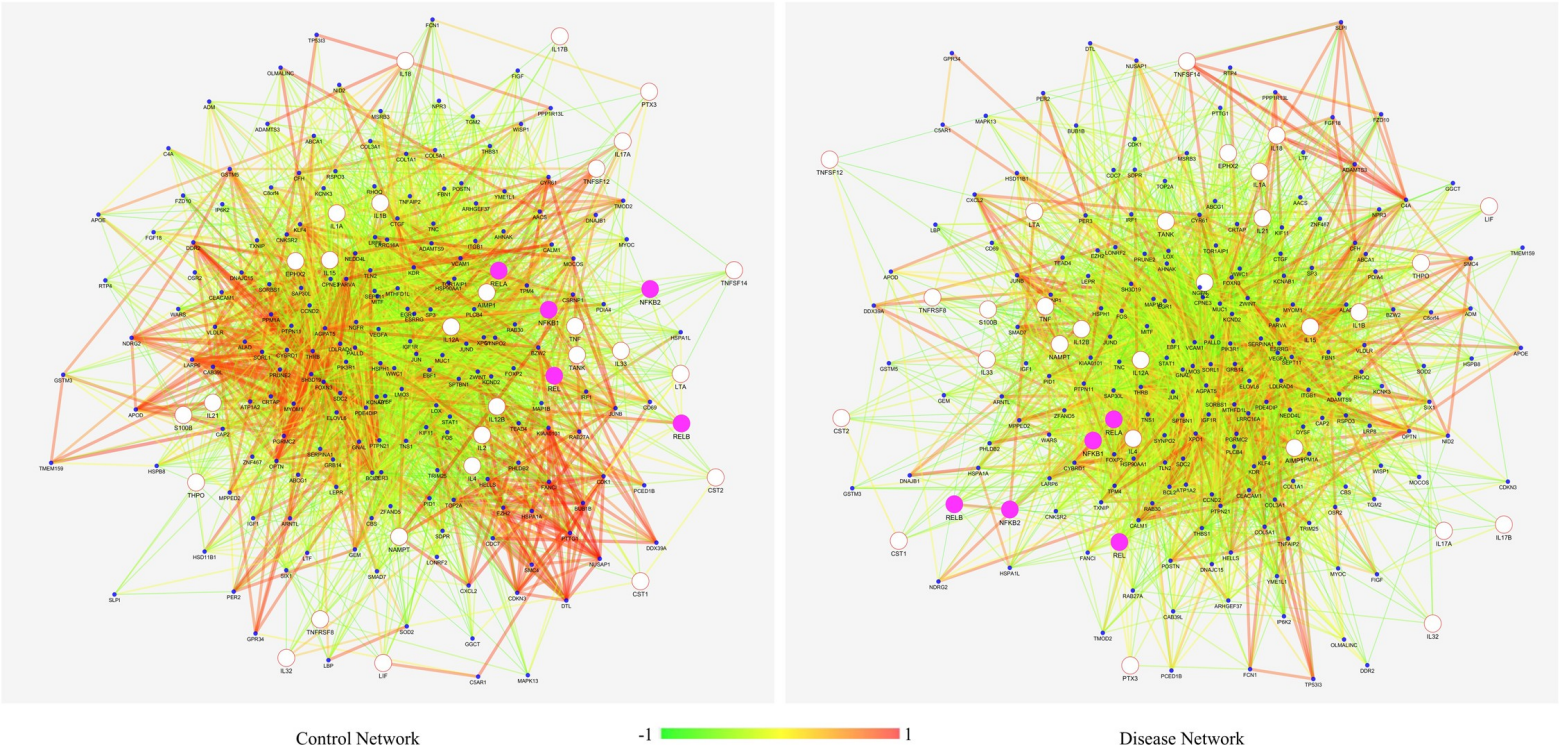
The prioritized network developed from control samples (control network) and disease samples (disease network). Control Network and Disease Network represent significant changes with strong altered connections of network connectivity from normal to obese state. The average connectivity of nodes in the control state is 15.42, and it has decreased to 14.75 in disease state depicts the overall loss in the interaction in obese condition.

**Fig 9:**
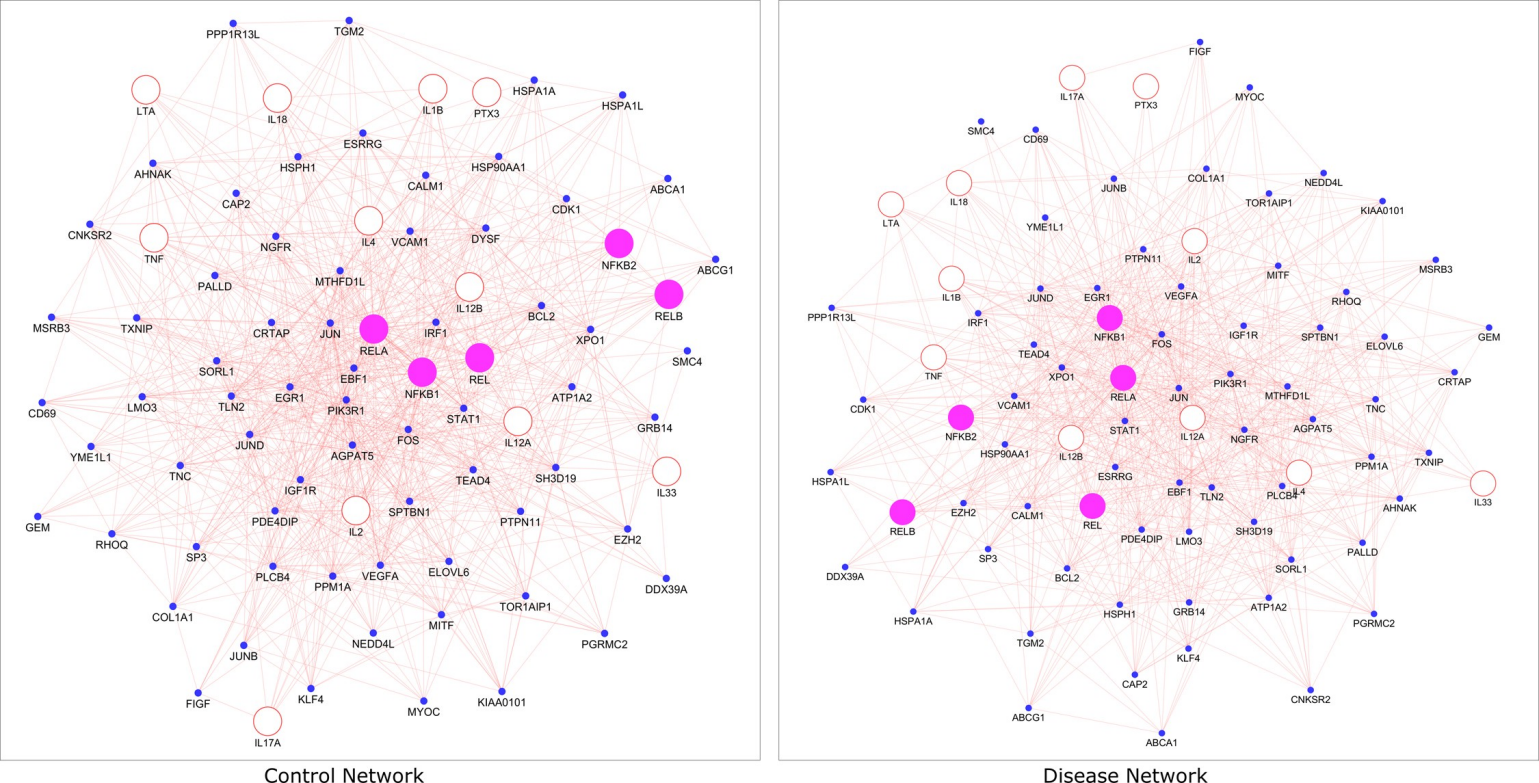
PPI network of control and disease. Genes connecting to the family of NF-κB proteins where pink nodes represent the NF-κB protein family.

An attempt was made to detect all potential interactions of filtered 193 genes, inflammatory inducers, and NF-κB protein family in both control and disease conditions to validate the analysis methods we applied in this study. This study was attempted because differentially co-expressed genes tend to associate in several biological processes which may lead to complementary or adverse effects [49]. The prioritized genes were extracted from S^PIN^ and co-expressed network for control and disease conditions separately. Next, we combined the control S^PIN^ network with control co-expression and disease S^PIN^ network with disease co-expression to create two separate sub-networks of disease and control genes (Fig 8). The newly generated *Control Network* and *Disease Network*, based on protein-protein interaction and co-expression interaction, were compared to delineate the major variations between them. We observed a significant alteration in the connectivity of genes from control to disease state. The connectivity of the nodes in *Control Network* is 3408, and it has decreased to 3259 in *Disease Network*, clearly indicating loss of functional partners in the overall disease network. There is also the loss of high correlation in *Disease Network*. Next, we focused on genes concerning their interaction with *NFKB1*, *NFKB2*, *REL*, *RELA*, and *RELB* (family of NF-κB proteins). Out of 193 genes, we obtained 68 genes with direct interaction to the family of NF-κB proteins as represented in Fig 9.

### Tissue specific analysis

**Fig 10:**
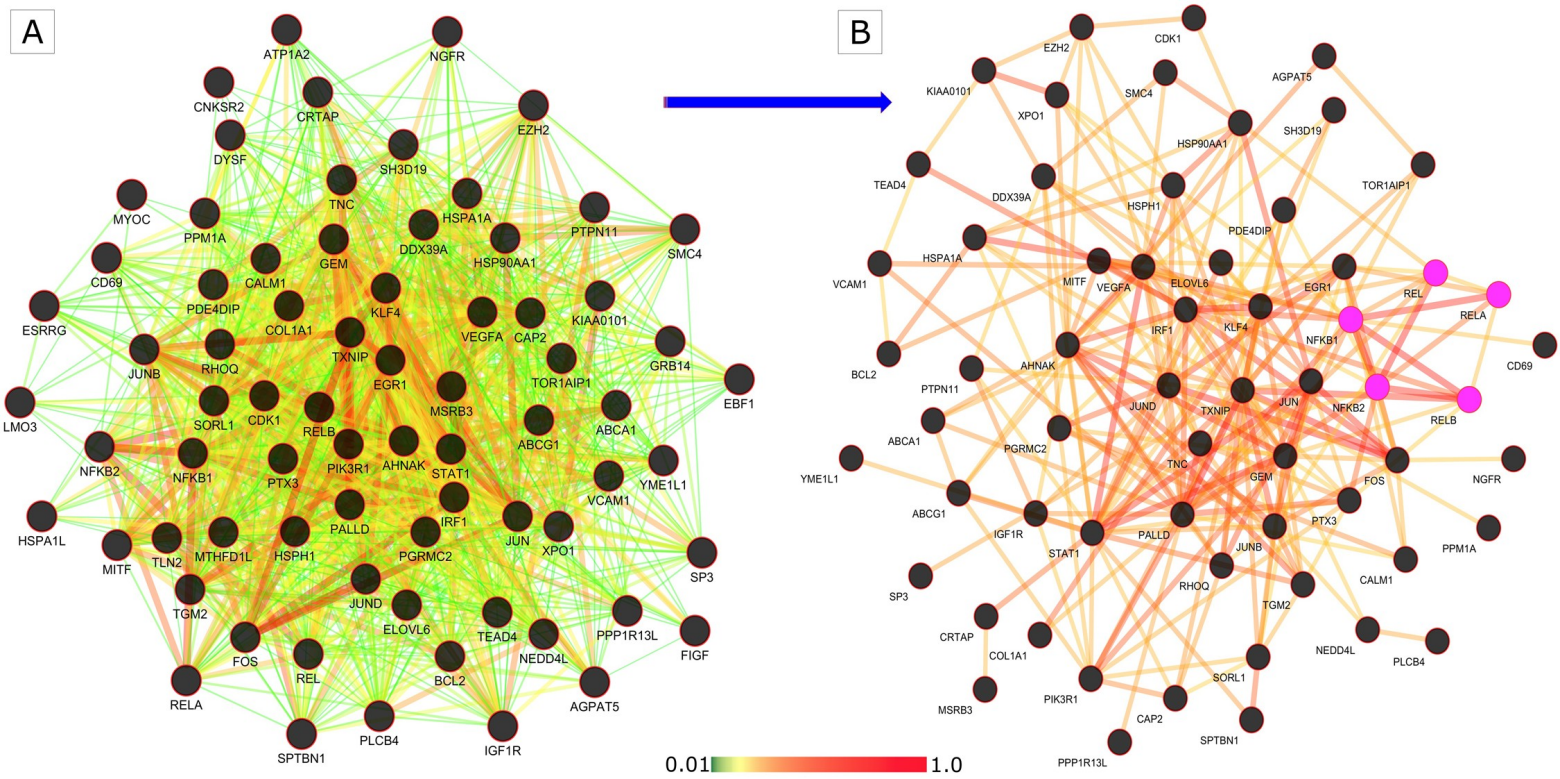
The expression and interaction of genes in adipose tissue using GIANT analysis. A) The dense network formed from 68 genes and proteins of the NF-κB family. B) The decomposed network based on the edge weight of 0.4 and above.

**Fig 11:**
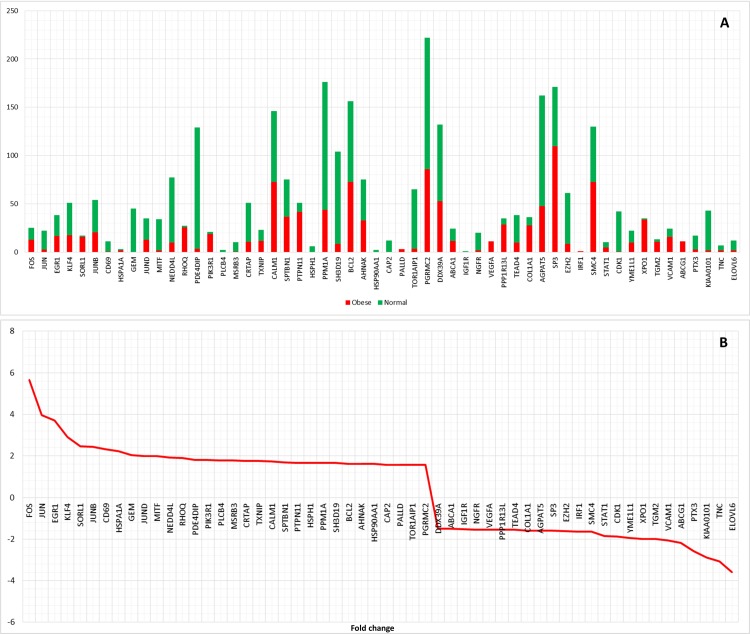
Genes connected to NF-κB protein family with their characteristics. A) Genes with their number of functional partners in obese and normal conditions, B) The fold change of these connected genes to the family of NF-κB proteins.

In GIANT, web interactive tool, we selected adipose tissue and queried 68 genes along with NF-κB proteins to check the tissue level expression of these genes. The tool generated an interactive network for the genes with specific edge weight ranging from 0 to 1. The tissue level expression and interaction of these genes were further filtered using a reasonable score of 0.4 and above. Thus, the dense network was decomposed to a smaller significant network with 56 candidate genes as shown in Fig 10. We further focused on fold change, change in some functional partners from normal to the obese condition of 56 genes which are connected to the NF-κB family. The most upregulated gene (FC = 5.65) is *FOS* (Fos Proto-Oncogene, AP-1 Transcription Factor Subunit) followed by *JUN* (Jun proto-oncogene, AP-1 transcription factor subunit) with an FC of 3.96. *ELOVL6* (ELOVL Fatty Acid Elongase 6) is the most downregulated gene (FC = -3.58) followed by *TNC* (Tenascin C) with FC of -3.07. The gene *SP3* (Sp3 transcription factor) has the highest deviation of functional partners from normal to obese followed by *PTPN11* (protein tyrosine phosphatase, non-receptor type 11). Various reports suggest genes described above connected to NF-κB protein family with distinct variations are involved in obesity or obesity related metabolic disorders [11, 50–52]. The overall landscape of these genes with their functional partners and fold change is depicted as a graph as shown in Fig 11.

**Fig 12:**
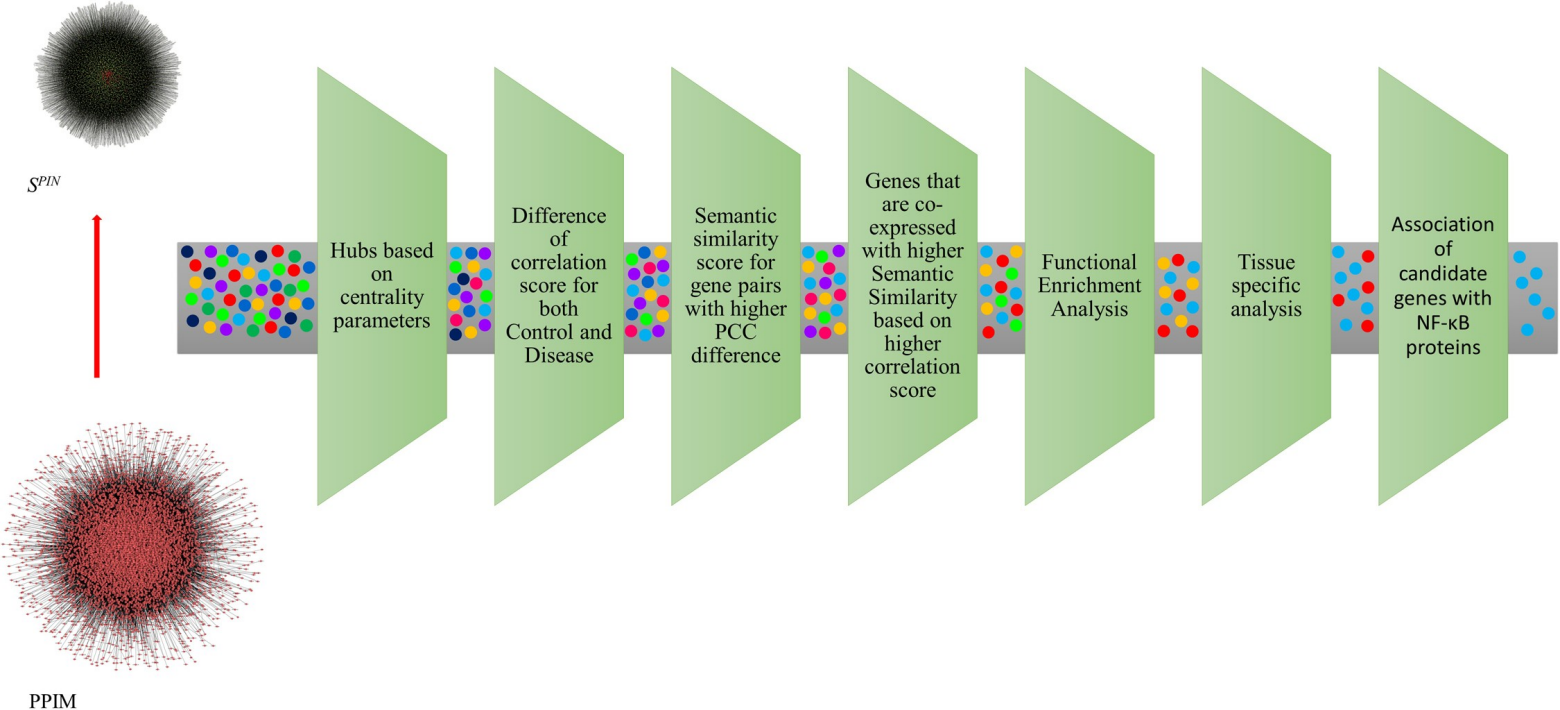
Pictorial representation of the filtering criteria used in the approach to identify biologically relevant functional nodes connected NF-κB proteins, obesity and related syndrome. A total of 2650 genes from PPIM was narrowed down to 21 target genes of NF-κB proteins associated with obesity using the filtering criteria centered on biological insights.

We performed extensive literature and database mining to pinpoint the role of 56 candidate genes obtained from the tissue analysis. We identified an association of 40 genes with obesity related pathways (S7 Table). The identified genes have shown strong functional association with obesity and related metabolic disorders. The genes like *VEGFA*, *ELOVL6*, *JUNB*, *PIK3R1*, *ABCG1*, *CD69*, *PTX3*, *SORL1*, *BCL2*, and *VCAM1* are reported with high impact elucidating its critical role in obesity and related metabolic syndrome. Interestingly, PTX3 is one of the inflammatory inducers of NF-κB proteins [45]. PTX3 is released in response to inflammation, and it regulates the immune response in association with NF-κB [46]. The genes are further interrelated with target genes of NF-κB protein family. About 23 genes were found to be the target genes of NF-κB protein family [53]. The identified target genes are *COL1A1*, *PIK3R1*, *VEGFA*, *TGM2*, *VCAM1*, *FOS*, *TXNIP*, *HSPA1A*, *IRF1*, *STAT1*, *GEM*, *PPP1R13L*, *IGF1R*, *HSP90AA1*, *TNC*, *EGR1*, *JUND*, *CD69*, *BCL2*, *PTX3*, *JUNB*, *JUN*, and *MITF*. Except for two genes (*GEM* and *PP1R13L*), all other 21 target proteins were found to be strongly associated with obesity or obesity related disorders. The genes *GEM* (GTP-binding protein GEM) and *PPP1R13L* (Protein Phosphatase 1 Regulatory Subunit 13 Like) are interesting target genes of NF-κB as they can act as novel targets for obesity or related syndrome. Twenty one target genes of NF-κB have shown strong association with obesity and the occurrence of *GEM* and *PPP1R13L* with known obesity related genes provides strong evidence to pick them as potential target genes. Hence, we report 23 genes as the most promising key signatures which are linked to NF-κB protein family and obesity or related syndrome. A pictorial representation of the filtering criteria we implemented in this research is represented in Fig 12.

## Discussion

Traditional approaches for gene expression exploration are centered on detecting single genes which show variations among two conditions of interest. Even though it is worthwhile, they do not identify biological processes, such as metabolic pathways, transcriptional regulations, and stress reactions that are spread through an entire gene’s network. Network biology is based on the concept that multifaceted or complex diseases generally do not progress due to disturbances in a single gene, but rather from alterations in complex pathways involving several interactions. Furthermore, biological processes inside our body are directed by the well-defined organization of protein complexes. In disease conditions, alterations in protein interaction network may lead to complementary effects through cascading events triggered by the deregulated protein to other proteins in the interacting network [12]. Network biology offers a platform to explore the biological and molecular mechanisms that could trigger the human disease. In this study, we explored the concepts of network biology, gene correlation, functional similarity and fold change to assess distinct variations related to the expression of the genes in obesity. We observed substantial alterations in the expression level of each prioritized gene in two experimental conditions. Here, it is important to highlight, that the genes prioritized in our approach are highly promising key signatures as they have following properties. (a) they show distinct variation in gene expression from a control state to disease state, (b) there is high functional similarity (semantic similarity) among the genes related to NF-κB protein family and obesity (d) they show distinct variation in functional partners from control to disease state.

**Fig 13:**
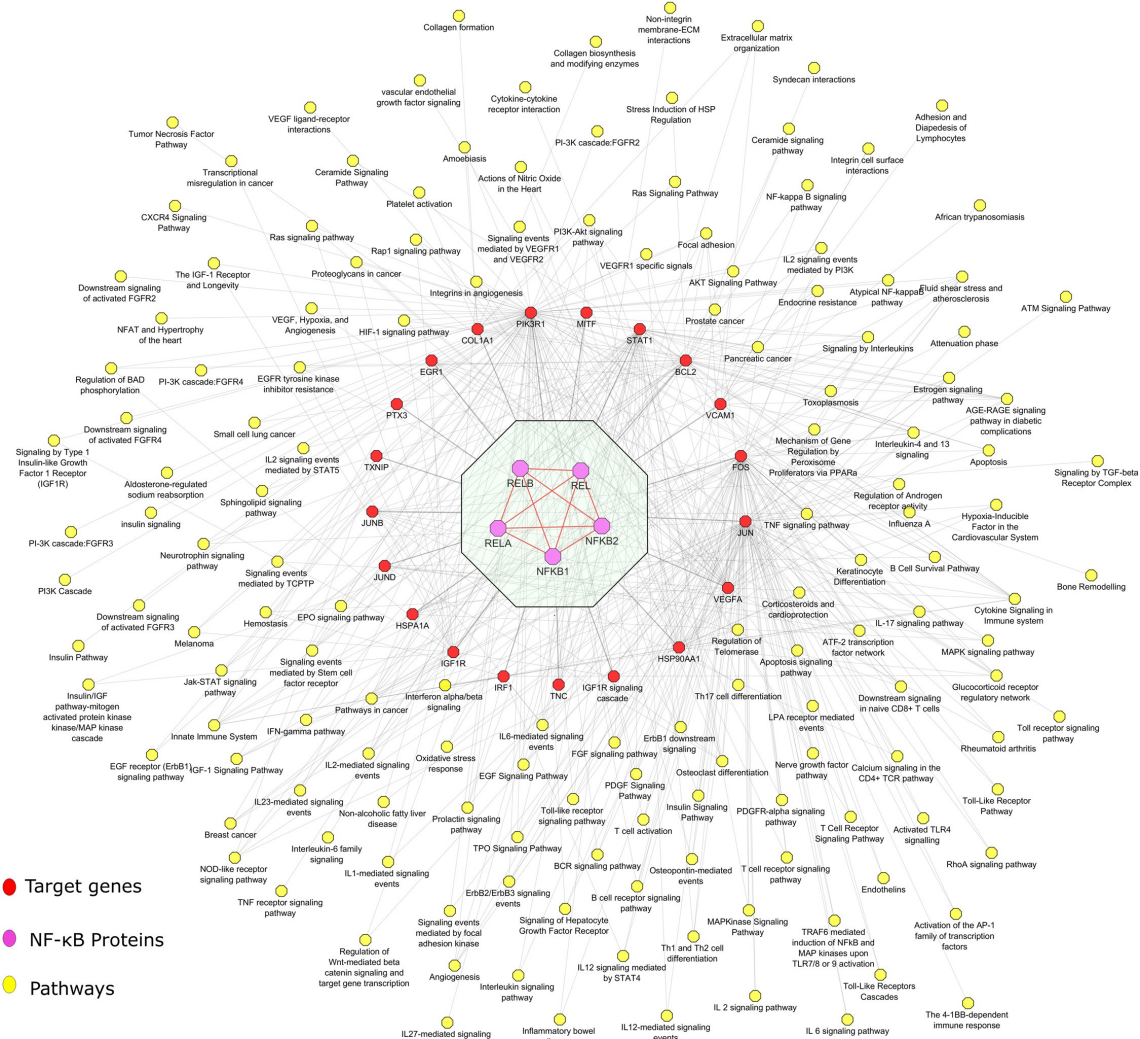
The pathway enrichment map. Thepotential target proteins of NF-κB protein family.

Implementing the computational pipeline, which is detailed in the methods section, we have narrowed down the number of genes to 40. Out of which 21 target proteins are from the NF-κB protein family that can act as potential biomarkers in obesity. Also, two promising genes GEM and PPP1R13L were predicted as novel potential biomarkers of obesity or related syndrome as they share characteristics of known obesity genes in the prioritized list. We performed extensive literature and database mining to pinpoint the role of these candidate genes obtained from the overall analysis. The genes like *VEGFA*, *JUNB*, *PIK3R1*, *CD69*, *PTX3*, *BCL2*, *IGF1R* and *VCAM1* are reported in the literature revealing their significant role in obesity and related metabolic syndrome. For example, Elias et al., (2013) [54] reports the potential role of *VEGFA* in the control of energy metabolism and adipose tissue function and Yu et al., (2016) [55] describes the association of *VCAM1* with obesity and inflammation markers. We developed a pathway map for the target proteins of NF-κB protein family from functional enrichment file as shown in Fig 13. The enrichment shows the pathways associated with these molecules are closely associated with obesity and inflammation. The molecules were involved in pathways like the NF-kappa B signaling pathway, Cytokine Signaling in Immune system, Insulin Signaling Pathway, MAPK signaling pathway, Angiogenesis, EGF Signaling Pathway, Glucocorticoid receptor regulatory network, Toll receptor signaling pathway, AKT Signaling Pathway, TNF signaling pathway, Focal adhesion, Interleukins signaling pathway, Jak-STAT signaling pathway and PI3K-Akt signaling pathway. Except for two genes (*GEM* and *PPP1R13L*), all other 21 target proteins were found to be strongly associated with obesity. Thus, we authenticate the genes identified through our approach are possible potential biomarkers of obesity or obesity related disorders. For example, glucocorticoid regulatory network contributes a major role in obesity as they are important hormones in the regulation of metabolic homeostasis [56]. Asensio et al., (2004) [57] reports that glucocorticoid hormones regulates the synthesis and discharge of hypothalamic neuropeptides, by inducing autonomic nervous system mediated processes and urge for excessive food intake. The action of the hypothalamopituitary-adrenal axis is seemingly raised in human obesity. The NF-κB target genes *IRF1*, *STAT1*, *JUN*, *HSP90AA1*, *FOS*, and *EGR1*, were enriched in the pathway of Glucocorticoid receptor regulatory network and homeostasis pointing towards their critical role in obesity.

Inflammatory pathways play a crucial role in metabolic disease like obesity. The NF-κB target genes *BCL2*, *STAT1*, *JUNB*, *PIK3R1*, *VCAM1*, *HSP90AA1*, *VEGFA*, and *FOS*, are involved in the pathway of signaling by interleukins. Similarly, *IRF1*, *BCL2*, *STAT1*, *JUNB*, *PIK3R1*, *VCAM1*, *HSP90AA1*, *VEGFA*, *FOS*, *and EGR1* are involved in *cytokine signaling in the immune system*. *Insulin signaling* and *PI3K-Akt signaling pathways* are another major pathways which influence obesity or related syndrome [58, 59]. Insulin is a critical modulator of all phases of adipocyte biology, and adipocytes are extremely insulin-responsive cell types. Insulin promotes adipocyte triglyceride stores by some mechanisms, including raising the differentiation of preadipocytes to adipocytes, triggering glucose transport and lipogenesis. The association among obesity and insulin resistance is seen across all racial groups and is evident across the full range of body weights. Also, many epidemiologic studies reveal that the risk for diabetes, and likely insulin resistance, increases as body fat content [4, 14]. The NF-κB target genes *JUN*, *PIK3R1*, *FOS*, and *IGF1R* are enriched in the *insulin signaling pathway* which could contribute to obesity related disorders. Similarly, NF-κB target genes enriched in pathways associated with obesity or related syndrome. The main pathways include *angiogenesis* [60] [61], *hypoxia* [62] [63], *oxidative stress* [64], t*oll-like receptor signaling pathway* [65]. The 23 filtered genes are correlated in multiple ways like gene pattern, functional similarity, association to NF-κB protein family and inflammatory inducers and have shown distinct deregulation from control to a disease state. These genes are also involved in pathways that are leading to obesity or related syndrome. This result anticipates that the potential properties of identified genes could be a possible target or biomarker for obesity or related disorders. Further validation using trials in the wet-lab, in vitro, and in vivo, are proposed to delineate the major impact of these potential genes in the etiopathogenesis.

## Conclusions

Overall, our integrative expression data analysis has revealed the molecular interaction network between NF-κB protein and other obesity associated candidate genes in adipose tissues. One of the interesting highlights of the study is two promising genes *GEM*, and *PPP1R13L* were predicted as novel potential biomarkers of obesity or related syndrome as they share characteristics of known obesity genes in the prioritized list. We further demonstrated that NF-kB regulates the inflammation in adipose tissues through several molecular signaling pathways like NF-kappa B, PI3K-Akt, Glucocorticoid receptor regulatory network, and Cytokine Signaling pathways. Our research can be further extended by experimentally validating the results using in vitro and in vivo approaches which will further help to identify selective therapeutic agents. Through this study, we showed how simultaneous protein interaction network-based approaches could be applied for elucidating functional protein interaction networks of NF-kB protein in complex diseases with inflammation background.

## Supporting information

S1 Table Go-annotations list of genes. (XLSX)

S2 Table Details of the NF-κB family of proteins. (PDF)

S3 Table The list of samples and their characteristics used in the research analysis. (PDF)

S4 Table List of inflammatory inducers of NF-κB proteins. (PDF)

S5 Table DPCC for normal vs obsese genes. (PDF)

S6 Table Sgo annotations based on DPcc in normal vs Obese. (PDF)

S7 Table List of genes involved in obesity or obesity related disorders with their fold change and statistical. (PDF)

## References

[1] Tanti JF, Ceppo F, Jager J, Berthou F. Implication of inflammatory signaling pathways in obesity-induced insulin resistance. Front Endocrinol (Lausanne). 2012;3:181 Epub 2013/01/15. 10.3389/fendo.2012.00181 23316186 PMC3539134

[2] Tzanavari T, Giannogonas P, Karalis KP. TNF-alpha and obesity. Curr Dir Autoimmun. 2010;11:145–56. Epub 2010/02/23. 10.1159/000289203 . 20173393

[3] Makki K, Froguel P, Wolowczuk I. Adipose tissue in obesity-related inflammation and insulin resistance: cells, cytokines, and chemokines. ISRN Inflamm. 2013;2013:139239 Epub 2014/01/24. 10.1155/2013/139239 24455420 PMC3881510

[4] Poloz Y, Stambolic V. Obesity and cancer, a case for insulin signaling. Cell Death Dis. 2015;6:e2037 Epub 2016/01/01. 10.1038/cddis.2015.381 26720346 PMC4720912

[5] Yamashita AS, Belchior T, Lira FS, Bishop NC, Wessner B, Rosa JC, et al Regulation of Metabolic Disease-Associated Inflammation by Nutrient Sensors. Mediators Inflamm. 2018;2018:8261432 Epub 2018/08/18. 10.1155/2018/8261432 30116154 PMC6079375

[6] Karin M, Cao Y, Greten FR, Li ZW. NF-kappaB in cancer: from innocent bystander to major culprit. Nat Rev Cancer. 2002;2(4):301–10. Epub 2002/05/11. 10.1038/nrc780 . 12001991

[7] Sun SC. Non-canonical NF-kappaB signaling pathway. Cell Res. 2011;21(1):71–85. Epub 2010/12/22. 10.1038/cr.2010.177 21173796 PMC3193406

[8] Malle EK, Zammit NW, Walters SN, Koay YC, Wu J, Tan BM, et al Nuclear factor kappaB-inducing kinase activation as a mechanism of pancreatic beta cell failure in obesity. J Exp Med. 2015;212(8):1239–54. Epub 2015/07/01. 10.1084/jem.20150218 26122662 PMC4516791

[9] Park MH, Hong JT. Roles of NF-kappaB in Cancer and Inflammatory Diseases and Their Therapeutic Approaches. Cells. 2016;5(2). Epub 2016/04/05. 10.3390/cells5020015 27043634 PMC4931664

[10] Liu SF, Malik AB. NF-kappa B activation as a pathological mechanism of septic shock and inflammation. Am J Physiol Lung Cell Mol Physiol. 2006;290(4):L622–L45. Epub 2006/03/15. 10.1152/ajplung.00477.2005 . 16531564

[11] Baker RG, Hayden MS, Ghosh S. NF-kappaB, inflammation, and metabolic disease. Cell Metab. 2011;13(1):11–22. Epub 2011/01/05. 10.1016/j.cmet.2010.12.008 21195345 PMC3040418

[12] Sevimoglu T, Arga KY. The role of protein interaction networks in systems biomedicine. Comput Struct Biotechnol J. 2014;11(18):22–7. Epub 2014/11/08. 10.1016/j.csbj.2014.08.008 25379140 PMC4212283

[13] Kreeger PK, Lauffenburger DA. Cancer systems biology: a network modeling perspective. Carcinogenesis. 2010;31(1):2–8. Epub 2009/10/29. 10.1093/carcin/bgp261 19861649 PMC2802670

[14] Cho DY, Kim YA, Przytycka TM. Chapter 5: Network biology approach to complex diseases. PLoS Comput Biol. 2012;8(12):e1002820 Epub 2013/01/10. 10.1371/journal.pcbi.1002820 23300411 PMC3531284

[15] Deng S, Qi J, Stephen M, Qiu L, Yang H. Network-based identification of reliable bio-markers for cancers. J Theor Biol. 2015;383:20–7. Epub 2015/08/08. 10.1016/j.jtbi.2015.07.026 . 26247140

[16] Albrecht M, Huthmacher C, Tosatto SC, Lengauer T. Decomposing protein networks into domain-domain interactions. Bioinformatics. 2005;21 Suppl 2:ii220–1. Epub 2005/10/06. 10.1093/bioinformatics/bti1135 . 16204107

[17] Zhu X, Gerstein M, Snyder M. Getting connected: analysis and principles of biological networks. Genes Dev. 2007;21(9):1010–24. Epub 2007/05/03. 10.1101/gad.1528707 . 17473168

[18] Harrill AH, Rusyn I. Systems biology and functional genomics approaches for the identification of cellular responses to drug toxicity. Expert Opin Drug Metab Toxicol. 2008;4(11):1379–89. Epub 2008/10/28. 10.1517/17425255.4.11.1379 18950280 PMC2614284

[19] Diaz-Beltran L, Cano C, Wall DP, Esteban FJ. Systems biology as a comparative approach to understand complex gene expression in neurological diseases. Behav Sci (Basel). 2013;3(2):253–72. Epub 2013/06/01. 10.3390/bs3020253 25379238 PMC4217627

[20] Bader S, Kuhner S, Gavin AC. Interaction networks for systems biology. FEBS Lett. 2008;582(8):1220–4. Epub 2008/02/20. 10.1016/j.febslet.2008.02.015 . 18282471

[21] Grigoriev A. A relationship between gene expression and protein interactions on the proteome scale: analysis of the bacteriophage T7 and the yeast Saccharomyces cerevisiae. Nucleic Acids Res. 2001;29(17):3513–9. Epub 2001/08/28. 11522820 10.1093/nar/29.17.3513 PMC55876

[22] Schlicker A, Lengauer T, Albrecht M. Improving disease gene prioritization using the semantic similarity of Gene Ontology terms. Bioinformatics. 2010;26(18):i561–7. Epub 2010/09/09. 10.1093/bioinformatics/btq384 20823322 PMC2935448

[23] Zhang L, Zhang J, Yang G, Wu D, Jiang L, Wen Z, et al Investigating the concordance of Gene Ontology terms reveals the intra- and inter-platform reproducibility of enrichment analysis. BMC Bioinformatics. 2013;14:143 Epub 2013/05/01. 10.1186/1471-2105-14-143 23627640 PMC3644270

[24] Vanunu O, Magger O, Ruppin E, Shlomi T, Sharan R. Associating genes and protein complexes with disease via network propagation. PLoS Comput Biol. 2010;6(1):e1000641 Epub 2010/01/22. 10.1371/journal.pcbi.1000641 20090828 PMC2797085

[25] Assenov Y, Ramirez F, Schelhorn SE, Lengauer T, Albrecht M. Computing topological parameters of biological networks. Bioinformatics. 2008;24(2):282–4. Epub 2007/11/17. 10.1093/bioinformatics/btm554 . 18006545

[26] Barrett T, Edgar R. Gene expression omnibus: microarray data storage, submission, retrieval, and analysis. Methods Enzymol. 2006;411:352–69. Epub 2006/08/31. 10.1016/S0076-6879(06)11019-8 16939800 PMC1619900

[27] Carvalho BS, Irizarry RA. A framework for oligonucleotide microarray preprocessing. Bioinformatics. 2010;26(19):2363–7. Epub 2010/08/07. 10.1093/bioinformatics/btq431 20688976 PMC2944196

[28] Smyth GK. limma: Linear Models for Microarray Data. In: Gentleman R, Carey VJ, Huber W, Irizarry RA, Dudoit S, editors. Bioinformatics and Computational Biology Solutions Using R and Bioconductor. New York, NY: Springer New York; 2005 p. 397–420.

[29] Irizarry RA, Hobbs B, Collin F, Beazer-Barclay YD, Antonellis KJ, Scherf U, et al Exploration, normalization, and summaries of high density oligonucleotide array probe level data. Biostatistics. 2003;4(2):249–64. Epub 2003/08/20. 10.1093/biostatistics/4.2.249 . 12925520

[30] Martin A, Ochagavia ME, Rabasa LC, Miranda J, Fernandez-de-Cossio J, Bringas R. BisoGenet: a new tool for gene network building, visualization and analysis. BMC Bioinformatics. 2010;11:91 Epub 2010/02/19. 10.1186/1471-2105-11-91 20163717 PMC3098113

[31] Albert R. Scale-free networks in cell biology. J Cell Sci. 2005;118(Pt 21):4947–57. Epub 2005/10/29. 10.1242/jcs.02714 . 16254242

[32] Barabasi AL, Gulbahce N, Loscalzo J. Network medicine: a network-based approach to human disease. Nat Rev Genet. 2011;12(1):56–68. Epub 2010/12/18. 10.1038/nrg2918 21164525 PMC3140052

[33] Shannon P, Markiel A, Ozier O, Baliga NS, Wang JT, Ramage D, et al Cytoscape: a software environment for integrated models of biomolecular interaction networks. Genome Res. 2003;13(11):2498–504. Epub 2003/11/05. 10.1101/gr.1239303 14597658 PMC403769

[34] Mesbah-Uddin M, Elango R, Banaganapalli B, Shaik NA, Al-Abbasi FA. In-silico analysis of inflammatory bowel disease (IBD) GWAS loci to novel connections. PLoS One. 2015;10(3):e0119420 Epub 2015/03/19. 10.1371/journal.pone.0119420 25786114 PMC4364731

[35] Csermely P, Korcsmaros T, Kiss HJ, London G, Nussinov R. Structure and dynamics of molecular networks: a novel paradigm of drug discovery: a comprehensive review. Pharmacol Ther. 2013;138(3):333–408. Epub 2013/02/07. 10.1016/j.pharmthera.2013.01.016 23384594 PMC3647006

[36] Xu J, Li Y. Discovering disease-genes by topological features in human protein-protein interaction network. Bioinformatics. 2006;22(22):2800–5. Epub 2006/09/07. 10.1093/bioinformatics/btl467 . 16954137

[37] Rakshit H, Rathi N, Roy D. Construction and analysis of the protein-protein interaction networks based on gene expression profiles of Parkinson's disease. PLoS One. 2014;9(8):e103047 Epub 2014/08/30. 10.1371/journal.pone.0103047 25170921 PMC4149362

[38] Lahiri C, Pawar S, Sabarinathan R, Ashraf MI, Chand Y, Chakravortty D. Interactome analyses of Salmonella pathogenicity islands reveal SicA indispensable for virulence. J Theor Biol. 2014;363:188–97. Epub 2014/08/17. 10.1016/j.jtbi.2014.08.013 . 25128737

[39] Yoon J, Blumer A, Lee K. An algorithm for modularity analysis of directed and weighted biological networks based on edge-betweenness centrality. Bioinformatics. 2006;22(24):3106–8. Epub 2006/10/25. 10.1093/bioinformatics/btl533 . 17060356

[40] Freeman LC, Borgatti SP, White DR. Centrality in valued graphs: A measure of betweenness based on network flow. Social Networks. 1991;13(2):141–54. 10.1016/0378-8733(91)90017-N.

[41] Wang JZ, Du Z, Payattakool R, Yu PS, Chen CF. A new method to measure the semantic similarity of GO terms. Bioinformatics. 2007;23(10):1274–81. Epub 2007/03/09. 10.1093/bioinformatics/btm087 . 17344234

[42] Yu G, Li F, Qin Y, Bo X, Wu Y, Wang S. GOSemSim: an R package for measuring semantic similarity among GO terms and gene products. Bioinformatics. 2010;26(7):976–8. Epub 2010/02/25. 10.1093/bioinformatics/btq064 . 20179076

[43] Azuaje FJ, Zhang L, Devaux Y, Wagner DR. Drug-target network in myocardial infarction reveals multiple side effects of unrelated drugs. Sci Rep. 2011;1:52 Epub 2012/02/23. 10.1038/srep00052 22355571 PMC3216539

[44] Banaganapalli B, Rashidi O, Saadah OI, Wang J, Khan IA, Al-Aama JY, et al Comprehensive Computational Analysis of GWAS Loci Identifies CCR2 as a Candidate Gene for Celiac Disease Pathogenesis. J Cell Biochem. 2017;118(8):2193–207. Epub 2017/01/07. 10.1002/jcb.25864 . 28059456

[45] Chen J, Bardes EE, Aronow BJ, Jegga AG. ToppGene Suite for gene list enrichment analysis and candidate gene prioritization. Nucleic Acids Res. 2009;37(Web Server issue):W305–11. Epub 2009/05/26. 10.1093/nar/gkp427 19465376 PMC2703978

[46] Greene CS, Krishnan A, Wong AK, Ricciotti E, Zelaya RA, Himmelstein DS, et al Understanding multicellular function and disease with human tissue-specific networks. Nat Genet. 2015;47(6):569–76. Epub 2015/04/29. 10.1038/ng.3259 25915600 PMC4828725

[47] Khosravi P, Gazestani VH, Asgari Y, Law B, Sadeghi M, Goliaei B. Network-based approach reveals Y chromosome influences prostate cancer susceptibility. Comput Biol Med. 2014;54:24–31. Epub 2014/09/10. 10.1016/j.compbiomed.2014.08.020 . 25199846

[48] Anglani R, Creanza TM, Liuzzi VC, Piepoli A, Panza A, Andriulli A, et al Loss of connectivity in cancer co-expression networks. PLoS One. 2014;9(1):e87075 Epub 2014/02/04. 10.1371/journal.pone.0087075 24489837 PMC3904972

[49] Hsu CL, Juan HF, Huang HC. Functional Analysis and Characterization of Differential Coexpression Networks. Sci Rep. 2015;5:13295 Epub 2015/08/19. 10.1038/srep13295 26282208 PMC4539605

[50] Matsuzaka T, Shimano H. Elovl6: a new player in fatty acid metabolism and insulin sensitivity. J Mol Med (Berl). 2009;87(4):379–84. Epub 2009/03/05. 10.1007/s00109-009-0449-0 . 19259639

[51] Matsuzaka T, Shimano H, Yahagi N, Kato T, Atsumi A, Yamamoto T, et al Crucial role of a long-chain fatty acid elongase, Elovl6, in obesity-induced insulin resistance. Nat Med. 2007;13(10):1193–202. Epub 2007/10/02. 10.1038/nm1662 . 17906635

[52] Tan CY, Virtue S, Bidault G, Dale M, Hagen R, Griffin JL, et al Brown Adipose Tissue Thermogenic Capacity Is Regulated by Elovl6. Cell Rep. 2015;13(10):2039–47. Epub 2015/12/03. 10.1016/j.celrep.2015.11.004 26628376 PMC4688035

[53] Pahl HL. Activators and target genes of Rel/NF-kappaB transcription factors. Oncogene. 1999;18(49):6853–66. Epub 1999/12/22. 10.1038/sj.onc.1203239 . 10602461

[54] Elias I, Franckhauser S, Bosch F. New insights into adipose tissue VEGF-A actions in the control of obesity and insulin resistance. Adipocyte. 2013;2(2):109–12. Epub 2013/06/28. 10.4161/adip.22880 23805408 PMC3661112

[55] Yu GI, Jun SE, Shin DH. Associations of VCAM-1 gene polymorphisms with obesity and inflammation markers. Inflamm Res. 2017;66(3):217–25. Epub 2016/11/18. 10.1007/s00011-016-1006-2 . 27853845

[56] Wang M. The role of glucocorticoid action in the pathophysiology of the Metabolic Syndrome. Nutr Metab (Lond). 2005;2(1):3 Epub 2005/02/04. 10.1186/1743-7075-2-3 15689240 PMC548667

[57] Asensio C, Muzzin P, Rohner-Jeanrenaud F. Role of glucocorticoids in the physiopathology of excessive fat deposition and insulin resistance. Int J Obes Relat Metab Disord. 2004;28 Suppl 4:S45–52. Epub 2004/12/14. 10.1038/sj.ijo.0802856 . 15592486

[58] Huang X, Liu G, Guo J, Su Z. The PI3K/AKT pathway in obesity and type 2 diabetes. Int J Biol Sci. 2018;14(11):1483–96. Epub 2018/09/29. 10.7150/ijbs.27173 30263000 PMC6158718

[59] Kahn SE, Hull RL, Utzschneider KM. Mechanisms linking obesity to insulin resistance and type 2 diabetes. Nature. 2006;444(7121):840–6. Epub 2006/12/15. 10.1038/nature05482 . 17167471

[60] Lemoine AY, Ledoux S, Larger E. Adipose tissue angiogenesis in obesity. Thromb Haemost. 2013;110(4):661–8. Epub 2013/04/19. 10.1160/TH13-01-0073 . 23595655

[61] Lijnen HR. Angiogenesis and obesity. Cardiovasc Res. 2008;78(2):286–93. Epub 2007/11/17. 10.1093/cvr/cvm007 . 18006485

[62] Regazzetti C, Peraldi P, Gremeaux T, Najem-Lendom R, Ben-Sahra I, Cormont M, et al Hypoxia decreases insulin signaling pathways in adipocytes. Diabetes. 2009;58(1):95–103. Epub 2008/11/06. 10.2337/db08-0457 18984735 PMC2606898

[63] Hodson L, Humphreys SM, Karpe F, Frayn KN. Metabolic signatures of human adipose tissue hypoxia in obesity. Diabetes. 2013;62(5):1417–25. Epub 2013/01/01. 10.2337/db12-1032 23274888 PMC3636615

[64] Furukawa S, Fujita T, Shimabukuro M, Iwaki M, Yamada Y, Nakajima Y, et al Increased oxidative stress in obesity and its impact on metabolic syndrome. J Clin Invest. 2004;114(12):1752–61. Epub 2004/12/16. 10.1172/JCI21625 15599400 PMC535065

[65] Vitseva OI, Tanriverdi K, Tchkonia TT, Kirkland JL, McDonnell ME, Apovian CM, et al Inducible Toll-like receptor and NF-kappaB regulatory pathway expression in human adipose tissue. Obesity (Silver Spring). 2008;16(5):932–7. Epub 2008/02/23. 10.1038/oby.2008.25 18292749 PMC3264059

