# Supplementary material for: Identification of key regulatory genes connected to NF-κB family of proteins in visceral adipose tissues using gene expression and weighted protein interaction network
Source: PLoS One. 2019 Apr 23;14(4):e0214337. doi: 10.1371/journal.pone.0214337 (PMC6478283; doi:10.1371/journal.pone.0214337)
Supplement: S2 Table — (PDF) [file pone.0214337.s002.pdf]

S2 Table. Details of the NF-κB family of proteins

Gene Symbol	Gene Name
NFKB1	Nuclear factor kappa B subunit 1
NFKB2	Nuclear factor kappa B subunit 2
REL	REL proto-oncogene, NF-kB subunit
RELA	RELA proto-oncogene, NF-kB subunit
RELB	RELB proto-oncogene, NF-kB subunit
