# Supplementary material for: Identification of key regulatory genes connected to NF-κB family of proteins in visceral adipose tissues using gene expression and weighted protein interaction network
Source: PLoS One. 2019 Apr 23;14(4):e0214337. doi: 10.1371/journal.pone.0214337 (PMC6478283; doi:10.1371/journal.pone.0214337)
Supplement: S3 Table — (PDF) [file pone.0214337.s003.pdf]

S3 Table: The list of samples and their characteristics used in the research analysis.

Sample	Tissue	BMI	Age	Height (cm)	Weight (kg)
GSM2349936	Visceral Adipose Tissue	23	18	160	58.3
GSM2349937	Visceral Adipose Tissue	19	16	155	45.1
GSM2349938	Visceral Adipose Tissue	25	19	162.5	68.3
GSM2349939	Visceral Adipose Tissue	56	17	178	176
GSM2349940	Visceral Adipose Tissue	22	13	157	53.4
GSM2349941	Visceral Adipose Tissue	22	11	162	58.8
GSM2349942	Visceral Adipose Tissue	24	17	173	72.1
GSM2349943	Visceral Adipose Tissue	46.9	17	165.7	128.7
GSM2349944	Visceral Adipose Tissue	19.6	16	157	48.2
GSM2349945	Visceral Adipose Tissue	39.8	14	163	105.7
GSM2349946	Visceral Adipose Tissue	37.8	15	159.4	96.1
GSM2349947	Visceral Adipose Tissue	21.7	16	160.5	54.8
GSM2349948	Visceral Adipose Tissue	15.9	12	150.75	36.1
GSM2349949	Visceral Adipose Tissue	30.5	14	162	79.7
GSM2349950	Visceral Adipose Tissue	62.5	17	157	154.1
GSM2349951	Visceral Adipose Tissue	39.5	17	151.5	90.6
GSM2349952	Visceral Adipose Tissue	23.4	15	168	66.2
GSM2349953	Visceral Adipose Tissue	55.4	21	162	145.4
GSM2349954	Visceral Adipose Tissue	56	14	158	140.4
GSM2349955	Visceral Adipose Tissue	17.8	14	153	41.6

GSM2349956	Visceral Adipose Tissue	41.7	17	159	105.5
GSM2349957	Visceral Adipose Tissue	62	17	161	160.8
GSM2349958	Visceral Adipose Tissue	24	14	165	65.4
GSM2349959	Visceral Adipose Tissue	24.6	17	170	71.2
GSM2349960	Visceral Adipose Tissue	21.8	16	162.6	57.6
GSM2349961	Visceral Adipose Tissue	49.1	15	174	148.5
GSM2349962	Visceral Adipose Tissue	46.4	16	147	100.3
GSM2349963	Visceral Adipose Tissue	36.3	19	171	106
GSM2349964	Visceral Adipose Tissue	45.5	18	165.2	127.5
GSM2349965	Visceral Adipose Tissue	20	12	155	47.4
