# Supplementary material for: Identification of key regulatory genes connected to NF-κB family of proteins in visceral adipose tissues using gene expression and weighted protein interaction network
Source: PLoS One. 2019 Apr 23;14(4):e0214337. doi: 10.1371/journal.pone.0214337 (PMC6478283; doi:10.1371/journal.pone.0214337)
Supplement: S4 Table — (PDF) [file pone.0214337.s004.pdf]

S4 Table: List of inflammatory inducers of NF-κB proteins

Symbol	Name
TNFRSF8	TNF receptor superfamily member 8
CST1	Cystatin SN
CST2	Cystatin SA
EPHX2	Epoxide hydrolase 2
IL1A	Interleukin 1 alpha
IL1B	Interleukin 1 beta
IL2	Interleukin 2
IL4	Interleukin 4
IL12A	Interleukin 12A
IL12B	Interleukin 12B
IL15	Interleukin 15
IL17A	Interleukin 17A
IL18	Interleukin 18
LIF	LIF, interleukin 6 family cytokine
LTA	Lymphotoxin alpha
PTX3	Pentraxin 3
S100B	S100 calcium binding protein B
THPO	Thrombopoietin
TNF	Tumor necrosis factor
TNFSF14	TNF superfamily member 14
TNFSF12	TNF superfamily member 12
IL32	Interleukin 32
AIMP1	Aminoacyl tRNA synthetase complex interacting multifunctional protein 1
TANK	TRAF family member associated NFKB activator
NAMPT	Nicotinamide phosphoribosyltransferase
IL17B	Interleukin 17B
IL21	Interleukin 21
IL33	Interleukin 33
