# Supplementary material for: Identification of key regulatory genes connected to NF-κB family of proteins in visceral adipose tissues using gene expression and weighted protein interaction network
Source: PLoS One. 2019 Apr 23;14(4):e0214337. doi: 10.1371/journal.pone.0214337 (PMC6478283; doi:10.1371/journal.pone.0214337)
Supplement: S5 Table — (PDF) [file pone.0214337.s005.pdf]

S5 Table: DPCC for Normal vs Obsese Genes

Gene-1	Gene-2	Normal	Obese	D_{PCC}	D_{PCC} (ABS)
TRIAP1	MAN2B2	-0.84490234	0.79061824	1.63552058	1.63552058
DCAF8	NFATC2IP	-0.8199923	0.7862996	1.6062919	1.6062919
P4HA1	PFDN4	0.8007077	-0.79464555	-1.59535325	1.59535325
XRCC5	EPHX2	-0.7465685	0.79317296	1.53974146	1.53974146
PCNA	RUFY3	-0.74981827	0.7793764	1.52919467	1.52919467
PSMB2	SPG20	-0.7259946	0.8019689	1.5279635	1.5279635
LRP8	THBS3	-0.8037855	0.71571183	1.51949733	1.51949733
MCM3	TOP2A	0.7113794	-0.7908391	-1.5022185	1.5022185
SP3	RCC2	-0.7283448	0.7502496	1.4785944	1.4785944
CCT3	MAN2B2	-0.75529975	0.708207	1.46350675	1.46350675
HSPA1L	HSPA1A	-0.7127358	0.7443941	1.4571299	1.4571299
MFAP3	EPHX2	-0.678268	0.77611774	1.45438574	1.45438574
DKC1	SP3	-0.6472255	0.80593705	1.45316255	1.45316255
MFAP3	BRAP	-0.7850248	0.66768545	1.45271025	1.45271025
XRCC5	RBM17	-0.6855559	0.765678	1.4512339	1.4512339
TUSC3	ABCA1	-0.7636935	0.68653065	1.45022415	1.45022415
STAT3	FOS	0.7200754	-0.7234661	-1.4435415	1.4435415
HPS6	ETNPPL	0.8087067	-0.63480866	-1.44351536	1.44351536
STX8	MRPL47	-0.7576042	0.68233097	1.43993517	1.43993517
CDK1	B3GNT2	0.7389807	-0.69810575	-1.43708645	1.43708645
HIVEP3	QSOX1	0.7733365	-0.66007346	-1.43340996	1.43340996
THBS3	SVEP1	0.8463368	-0.5855048	-1.4318416	1.4318416
CD47	CTNNA1	-0.8061776	0.62431586	1.43049346	1.43049346
ZNF384	ZNF704	0.79648143	-0.6303119	-1.42679333	1.42679333
EPS15	MRPS22	-0.63913375	0.7862456	1.42537935	1.42537935
TEX10	PELP1	0.7758664	-0.6404441	-1.4163105	1.4163105
XRCC5	RUFY3	-0.67382026	0.74105644	1.4148767	1.4148767
NEDD4L	PRICKLE1	0.7761754	-0.6375003	-1.4136757	1.4136757
MCM3	HLA-C	-0.60021347	0.81271887	1.41293234	1.41293234
MRPL17	POC1A	0.8826445	-0.52615	-1.4087945	1.4087945
AGPAT5	MAN2B2	-0.6860501	0.7226621	1.4087122	1.4087122
ATP1A2	SOX4	0.7191575	-0.6887498	-1.4079073	1.4079073
PI3	PDIA4	0.6507124	-0.7532877	-1.4040001	1.4040001
PI3	ADAMTS9	0.6032839	-0.7922514	-1.3955353	1.3955353
KARS	RMDN1	-0.71330583	0.68134165	1.39464748	1.39464748
SOX4	SFRP1	0.8183535	-0.5759782	-1.3943317	1.3943317
EPHX2	PSMB3	-0.8338243	0.5598863	1.3937106	1.3937106
ESRRG	TBC1D9B	-0.80233085	0.58972096	1.39205181	1.39205181
COL4A1	FZR1	0.7556257	-0.6345547	-1.3901804	1.3901804
EFCAB14	LPCAT1	-0.59437317	0.79569143	1.3900646	1.3900646
NDN	SOX4	0.7040219	-0.68534976	-1.38937166	1.38937166
EPHA1	MRPS22	0.6473207	-0.74086857	-1.38818927	1.38818927
KIRREL	LCMT2	-0.6944882	0.69140863	1.38589683	1.38589683
TOP2A	BIK	0.8647102	-0.52093965	-1.38564985	1.38564985
SP3	DNMT1	-0.56702673	0.817593	1.38461973	1.38461973
EFCAB14	WDR46	-0.59951544	0.78226036	1.3817758	1.3817758
ESRRG	RPRD1A	-0.6117029	0.7674302	1.3791331	1.3791331
CSTF3	HLA-G	-0.59477663	0.783978	1.37875463	1.37875463
EPS15	RACGAP1	-0.6537358	0.72451884	1.37825464	1.37825464
COCH	PPP1R1B	0.6980992	-0.68010455	-1.37820375	1.37820375
CTNNA1	TPBG	-0.77409065	0.60245144	1.37654209	1.37654209
GTF2I	GALNT2	-0.7416724	0.6347024	1.3763748	1.3763748
NEGR1	DDX28	0.73135704	-0.6444404	-1.37579744	1.37579744

ZNF608	C1orf131	-0.46701208	0.9074724	1.37448448	1.37448448
HLF	PRICKLE1	0.76242656	-0.61179984	-1.3742264	1.3742264
SERPINA1	COL5A1	-0.50202477	0.8716276	1.37365237	1.37365237
SCML2	PER2	-0.7892402	0.5836983	1.3729385	1.3729385
TMEM223	HLA-C	-0.7572834	0.61365175	1.37093515	1.37093515
EIF4G3	KPNA4	0.74106145	-0.62982756	-1.37088901	1.37088901
KALRN	NUDCD2	-0.73665214	0.6327213	1.36937344	1.36937344
CCT3	KCNRG	0.6988981	-0.67004406	-1.36894216	1.36894216
FHOD1	EXOSC8	-0.6887996	0.67712444	1.36592404	1.36592404
MLH1	PDE7A	0.75778586	-0.6068467	-1.36463256	1.36463256
POLN	CEP112	-0.7310638	0.6333874	1.3644512	1.3644512
RAB35	COG6	0.640026	-0.7240482	-1.3640742	1.3640742
QSOX1	TOB2	0.77924407	-0.5846613	-1.36390537	1.36390537
FIP1L1	XXYLT1	-0.7358085	0.6272164	1.3630249	1.3630249
TRIT1	MFAP3	-0.5800675	0.78246534	1.36253284	1.36253284
PTTG1IP	TOP3A	-0.7134585	0.6479284	1.3613869	1.3613869
PDE4DIP	DPAGT1	-0.6922102	0.6686902	1.3609004	1.3609004
NOL9	ASB1	-0.55212194	0.8078885	1.36001044	1.36001044
TRIM27	ABCA1	-0.64169246	0.7181909	1.35988336	1.35988336
ADRA1B	LAP3	0.5426458	-0.81597626	-1.35862206	1.35862206
MCM6	KALRN	-0.61027664	0.74819267	1.35846931	1.35846931
PDZD2	STX6	-0.61206543	0.74599236	1.35805779	1.35805779
HDAC1	RBM17	-0.6978421	0.6587166	1.3565587	1.3565587
WDTC1	SLC25A22	-0.76698816	0.58927554	1.3562637	1.3562637
CNNM2	BST2	0.82219315	-0.5334293	-1.35562245	1.35562245
KIRREL	NIPA2	-0.63683945	0.71779954	1.35463899	1.35463899
MCM3	ZBTB10	-0.60310096	0.7494847	1.35258566	1.35258566
LMO3	SOX4	0.6061915	-0.7387266	-1.3449181	1.3449181
OPRK1	GPR34	-0.69782233	0.6470283	1.34485063	1.34485063
ESRRG	ALAD	-0.52817047	0.81636673	1.3445372	1.3445372
LRP8	BMP1	-0.5808839	0.76257086	1.34345476	1.34345476
LCMT2	PCMTD1	-0.5500099	0.79282504	1.34283494	1.34283494
EZH2	CDK1	0.89854753	-0.4441706	-1.34271813	1.34271813
MCM3	RBM17	-0.6941539	0.64797425	1.34212815	1.34212815
NEGR1	MSRB3	-0.8506514	0.4910484	1.3416998	1.3416998
MMP14	CADM1	0.76475376	-0.57371956	-1.33847332	1.33847332
NOVA1	ATP5G1	-0.8101079	0.5280324	1.3381403	1.3381403
SOX4	DTL	-0.5858822	0.7506319	1.3365141	1.3365141
RDH11	PFDN4	0.7590253	-0.5772304	-1.3362557	1.3362557
PARK2	FTL	-0.56185335	0.77378285	1.3356362	1.3356362
MSR1	CCNI	0.5157306	-0.81955284	-1.33528344	1.33528344
CSTF3	NEGR1	0.63993984	-0.6943373	-1.33427714	1.33427714
ITSN1	ESRRG	-0.66610426	0.66731447	1.33341873	1.33341873
CDC7	CDK10	-0.71748704	0.61478335	1.33227039	1.33227039
QKI	C1orf131	-0.58601725	0.74562836	1.33164561	1.33164561
THRB	MFAP3	-0.5918367	0.73952407	1.33136077	1.33136077
NFE2L1	DPAGT1	-0.7248667	0.6051535	1.3300202	1.3300202
PDE4DIP	SLIRP	-0.5743951	0.7516256	1.3260207	1.3260207
RRBP1	FOS	0.73889977	-0.5868063	-1.32570607	1.32570607
KARS	OSR2	-0.7589206	0.56658095	1.32550155	1.32550155
RBM17	PHF5A	-0.6138776	0.71145266	1.32533026	1.32533026
FOXN3	MRPL47	-0.72816193	0.59584767	1.3240096	1.3240096
CCND2	PPP2R4	0.78921294	-0.5342362	-1.32344914	1.32344914
EIF4G3	MGEA5	-0.56705284	0.75601536	1.3230682	1.3230682
ARHGEF12	UBE2B	-0.610231	0.7122044	1.3224354	1.3224354

SAP18	SIRPA	0.9085426	-0.41319418	-1.32173678	1.32173678
ZNF608	PNPT1	-0.53114	0.79026884	1.32140884	1.32140884
BRD8	TM4SF18	-0.45721927	0.86286503	1.3200843	1.3200843
EIF4E3	CERS5	0.8238425	-0.4959678	-1.3198103	1.3198103
IDS	AGPAT5	-0.53074205	0.7887671	1.31950915	1.31950915
SPCS3	CCDC90B	-0.5817221	0.7374461	1.3191682	1.3191682
ARHGEF2	RCC2	-0.88251185	0.43579084	1.31830269	1.31830269
PCNA	ZBTB10	-0.6408693	0.67680013	1.31766943	1.31766943
GALNT2	ADGRA2	-0.5844702	0.7323404	1.3168106	1.3168106
MFAP3	PDZRN3	-0.5995868	0.7171017	1.3166885	1.3166885
HDGF	COL5A1	-0.71222186	0.60277355	1.31499541	1.31499541
CADM1	KIAA1715	-0.68141055	0.63299024	1.31440079	1.31440079
TOP2A	PNPT1	0.65077364	-0.6601712	-1.31094484	1.31094484
MCM3	TM4SF18	-0.47840026	0.8325208	1.31092106	1.31092106
CUX1	CTNNA1	-0.80673116	0.5041051	1.31083626	1.31083626
INO80D	DLC1	-0.7321852	0.57778245	1.30996765	1.30996765
GTF2I	STX6	-0.7897	0.52001107	1.30971107	1.30971107
EPHA1	KIAA0391	0.57188904	-0.73669785	-1.30858689	1.30858689
MAP4K4	ZFP36	0.6681097	-0.64027995	-1.30838965	1.30838965
DNAJC15	CDKN3	-0.89037764	0.4160168	1.30639444	1.30639444
PCNA	TOP2A	0.816714	-0.48899704	-1.30571104	1.30571104
SGCB	MRPS22	-0.62789446	0.67756397	1.30545843	1.30545843
CDK10	RELA	-0.627355	0.67696977	1.30432477	1.30432477
ESRRG	MRPL19	-0.58941305	0.7143872	1.30380025	1.30380025
NEDD4L	IL17RA	-0.6791939	0.62367463	1.30286853	1.30286853
B2M	DHX30	-0.6630218	0.63950795	1.30252975	1.30252975
LDLRAD4	CNOT11	-0.6005252	0.7011989	1.3017241	1.3017241
SNRPB2	RBM17	-0.53370345	0.76769567	1.30139912	1.30139912
CTNNA1	PCDHB5	-0.6348016	0.6657191	1.3005207	1.3005207
MCM3	RGS5	-0.76207066	0.5379213	1.29999196	1.29999196
PTGER3	COL1A1	0.6447596	-0.6545425	-1.2993021	1.2993021
COL15A1	CECR5	-0.5350179	0.7624805	1.2974984	1.2974984
KIF11	SOBP	-0.82528615	0.47181284	1.29709899	1.29709899
ELF4	SAMD4A	0.7990669	-0.49798268	-1.29704958	1.29704958
CPNE3	SOX4	0.6887324	-0.60812926	-1.29686166	1.29686166
NAALADL2	GGCT	-0.6228409	0.67354214	1.29638304	1.29638304
TUSC3	MTMR4	-0.41833493	0.87791765	1.29625258	1.29625258
RACGAP1	KCNRG	0.6094859	-0.68615985	-1.29564575	1.29564575
VPS16	MFSD12	-0.49231207	0.8033327	1.29564477	1.29564477
SPAG16	CNOT11	-0.6496571	0.6456986	1.2953557	1.2953557
ADAM22	ARHGEF37	0.7522212	-0.5429671	-1.2951883	1.2951883
COPZ1	EPHX2	-0.48643735	0.8078363	1.29427365	1.29427365
CDK6	IL1A	-0.6573744	0.635505	1.2928794	1.2928794
ESRRG	PARVA	-0.6211608	0.67109597	1.29225677	1.29225677
EIF4G3	ARPP19	0.7480565	-0.5440928	-1.2921493	1.2921493
IPO11	ABCA1	-0.6168594	0.6731664	1.2900258	1.2900258
ANKRD28	LCLAT1	-0.5706625	0.71929157	1.28995407	1.28995407
COL3A1	PHF8	-0.49609336	0.793753	1.28984636	1.28984636
CRYZL1	TP53I3	0.6626646	-0.6270307	-1.2896953	1.2896953
NVL	COL6A3	-0.61434007	0.67510545	1.28944552	1.28944552
PDZRN3	DPAGT1	-0.7556063	0.53298956	1.28859586	1.28859586
GNAQ	CNOT11	-0.55279833	0.73550224	1.28830057	1.28830057
SAP18	TNFRSF25	0.69594306	-0.59230566	-1.28824872	1.28824872
CDCA7	KALRN	-0.55688703	0.73099923	1.28788626	1.28788626
TNFAIP2	HGF	0.71663713	-0.5712255	-1.28786263	1.28786263

ESRRG	PDE4DIP	-0.63184583	0.65505725	1.28690308	1.28690308
QKI	CNOT11	-0.55218977	0.73432446	1.28651423	1.28651423
HDAC11	DKC1	-0.7272024	0.55897725	1.28617965	1.28617965
SMYD3	MDN1	-0.7415718	0.5442571	1.2858289	1.2858289
MCM3	THRB	-0.71150345	0.5740923	1.28559575	1.28559575
TRIAP1	SH3RF1	-0.48181644	0.80352235	1.28533879	1.28533879
LAMA4	POLDIP3	-0.68610704	0.59920406	1.2853111	1.2853111
SOX4	BMPER	0.49909237	-0.78522354	-1.28431591	1.28431591
STAT5B	SOX4	0.54572093	-0.73821187	-1.2839328	1.2839328
SRD5A1	ADAMTS2	-0.510949	0.7729102	1.2838592	1.2838592
KALRN	TMEM97	-0.6447231	0.6385976	1.2833207	1.2833207
COL9A1	MEST	0.774731	-0.5073037	-1.2820347	1.2820347
CDK10	TEAD4	-0.6133582	0.6686476	1.2820058	1.2820058
COL3A1	LAMA1	-0.65799004	0.62341124	1.28140128	1.28140128
ACPT	KLHL34	-0.51420414	0.7670853	1.28128944	1.28128944
XRCC5	SIRPA	-0.5966819	0.6845096	1.2811915	1.2811915
HLA-C	PUS1	-0.5816759	0.69945097	1.28112687	1.28112687
NIPA2	PDE7A	0.5098701	-0.7702751	-1.2801452	1.2801452
ZBTB10	HDAC1	-0.6360605	0.64366376	1.27972426	1.27972426
KIRREL	LCLAT1	-0.44309118	0.83655965	1.27965083	1.27965083
SRD5A1	COL6A3	-0.6223125	0.65725946	1.27957196	1.27957196
UBC	TEC	0.75500816	-0.5243374	-1.27934556	1.27934556
PSMB2	RNF150	-0.9076755	0.37138423	1.27905973	1.27905973
CSF3R	ZDHHC14	-0.6145394	0.6642676	1.278807	1.278807
RBM17	SLIRP	-0.5577912	0.7207314	1.2785226	1.2785226
MCM6	TM4SF18	-0.6228355	0.6550845	1.27792	1.27792
ESRRG	SORBS1	-0.54498845	0.73174226	1.27673071	1.27673071
CSNK2A1	QKI	-0.47369668	0.80256504	1.27626172	1.27626172
HDGF	SOX4	-0.6647953	0.611414	1.2762093	1.2762093
XPO5	SP3	-0.43465114	0.8414475	1.27609864	1.27609864
NIPA2	MAN2B2	-0.52879214	0.74604195	1.27483409	1.27483409
LAMA1	THBS3	-0.6169418	0.65742236	1.27436416	1.27436416
KARS	VAV2	0.7770853	-0.4971271	-1.2742124	1.2742124
PPA1	TCEA3	-0.842074	0.43186954	1.27394354	1.27394354
TDRKH	KCTD20	-0.7611879	0.51246566	1.27365356	1.27365356
LTA	ATP5SL	0.61790764	-0.6557371	-1.27364474	1.27364474
MRPL47	ESD	-0.65567225	0.6177055	1.27337775	1.27337775
KSR1	DHCR7	-0.7011429	0.5712737	1.2724166	1.2724166
ZNF608	NIPA2	-0.4183757	0.8539921	1.2723678	1.2723678
ACVR2A	OLMALINC	-0.61134523	0.66063213	1.27197736	1.27197736
BRAP	NT5DC1	-0.51194835	0.75973016	1.27167851	1.27167851
FOS	ARPP21	-0.6748435	0.5968049	1.2716484	1.2716484
MOB3B	SNRPC	-0.49947715	0.7718912	1.27136835	1.27136835
AIDA	C10orf54	0.66780835	-0.60333085	-1.2711392	1.2711392
SH3D19	PRICKLE1	0.588061	-0.68275344	-1.27081444	1.27081444
RCC2	CPXM1	-0.48165482	0.78911847	1.27077329	1.27077329
ESRRG	EHBP1	-0.66181755	0.6088334	1.27065095	1.27065095
CLCN3	ZSCAN20	-0.66053903	0.60970634	1.27024537	1.27024537
STK10	EGR1	0.8542187	-0.41553533	-1.26975403	1.26975403
PCDH17	TOP3A	-0.60044414	0.668883	1.26932714	1.26932714
MRPS14	HPS6	-0.5825855	0.6865051	1.2690906	1.2690906
ZHX2	ZNF202	-0.4670785	0.8016713	1.2687498	1.2687498
CASP8	GART	0.57553095	-0.69290364	-1.26843459	1.26843459
ESRRG	KANK1	-0.4917046	0.77668875	1.26839335	1.26839335
VCAM1	PPIL2	-0.5320185	0.7362928	1.2683113	1.2683113

APC	POLR2G	-0.47394142	0.7939028	1.26784422	1.26784422
CSTF3	HLA-B	-0.40256575	0.86526024	1.26782599	1.26782599
PPIL2	TBC1D1	-0.7138947	0.55367076	1.26756546	1.26756546
RSPO3	SLC25A29	0.54650295	-0.72099197	-1.26749492	1.26749492
CYCS	QSOX1	0.7508814	-0.5158037	-1.2666851	1.2666851
MFAP3	NFIA	-0.6850633	0.5813792	1.2664425	1.2664425
MMD	SOX4	0.5056489	-0.76073194	-1.26638084	1.26638084
TGM2	TYW5	0.7522273	-0.51390797	-1.26613527	1.26613527
APC	NUTF2	-0.61029077	0.65565896	1.26594973	1.26594973
PPIL2	AMOT	0.71436757	-0.5507488	-1.26511637	1.26511637
KPNB1	DCAF7	-0.4774065	0.7871937	1.2646002	1.2646002
MCM6	RUFY3	-0.7507518	0.5137244	1.2644762	1.2644762
DKC1	TOP2A	0.7207499	-0.5431717	-1.2639216	1.2639216
MOCS2	INPP5F	-0.67144376	0.5921187	1.26356246	1.26356246
CASP8	CSTF3	0.39117992	-0.87193155	-1.26311147	1.26311147
SPCS3	SP3	-0.42879662	0.8339435	1.26274012	1.26274012
RACGAP1	NFE2L1	-0.6629	0.599812	1.262712	1.262712
ITGB6	MYOF	0.6040414	-0.658583	-1.2626244	1.2626244
MCM4	BST2	0.57062817	-0.69127506	-1.26190323	1.26190323
DNAJC15	MRPL17	-0.8364428	0.42526132	1.26170412	1.26170412
MFAP3	SSPN	-0.5191965	0.7422256	1.2614221	1.2614221
NFIA	AMMECR1	0.66065186	-0.600358	-1.26100986	1.26100986
EFCAB14	EFHD2	-0.5642069	0.6961921	1.260399	1.260399
PTGER3	PRICKLE1	0.56714195	-0.6925308	-1.25967275	1.25967275
MFAP3	GEMIN4	-0.6569424	0.6026585	1.2596009	1.2596009
RAE1	DCAF8	-0.418044	0.8405329	1.2585769	1.2585769
SFN	SNU13	0.64818513	-0.6103026	-1.25848773	1.25848773
ESRRG	THRB	-0.5717415	0.6863906	1.2581321	1.2581321
PAPPA	CTNNA1	-0.5816931	0.67631376	1.25800686	1.25800686
RAB27A	LAGE3	0.5993815	-0.65711904	-1.25650054	1.25650054
ESRRG	CALM1	-0.48427963	0.7721957	1.25647533	1.25647533
CREB3L4	ATG4B	-0.49268857	0.76356953	1.2562581	1.2562581
STAT3	ARNTL	-0.43507928	0.82089096	1.25597024	1.25597024
CUX1	COL15A1	-0.6346019	0.62125856	1.25586046	1.25586046
GYS1	ZSCAN20	-0.55446225	0.701254	1.25571625	1.25571625
TK1	DDAH2	-0.7263438	0.52926314	1.25560694	1.25560694
LOC10099669:	DNAJC9	0.52931434	-0.7259336	-1.25524794	1.25524794
MUC1	CTNNA1	-0.85999155	0.39518526	1.25517681	1.25517681
TUSC3	ARHGEF2	-0.6531047	0.6008362	1.2539409	1.2539409
COL3A1	PRMT8	-0.83920074	0.41447073	1.25367147	1.25367147
SOAT1	STIM2	-0.4096158	0.8435954	1.2532112	1.2532112
ARPC5L	FAXDC2	-0.8226248	0.43036345	1.25298825	1.25298825
RAB11A	RAB40B	-0.6919919	0.56070274	1.25269464	1.25269464
ESRRG	CYBRD1	-0.58438814	0.6679339	1.25232204	1.25232204
EBP	ACAT2	0.7905813	-0.4616097	-1.252191	1.252191
ACVR2A	TRIT1	-0.59917027	0.65285087	1.25202114	1.25202114
GNAL	NAB2	0.4851049	-0.7668596	-1.2519645	1.2519645
RUFY3	SRPRB	-0.59400475	0.6566459	1.25065065	1.25065065
RGS5	HDAC1	-0.7012007	0.5490282	1.2502289	1.2502289
CTGF	BGN	0.9142953	-0.33578956	-1.25008486	1.25008486
GMFG	MDN1	-0.83810407	0.41161686	1.24972093	1.24972093
TCEB3	TCF3	0.5512225	-0.69814813	-1.24937063	1.24937063
CDK6	LIPF	-0.5555869	0.69353265	1.24911955	1.24911955
COLGALT1	USP3	-0.6202603	0.6279459	1.2482062	1.2482062
XRCC5	DHRS13	0.75274116	-0.4953637	-1.24810486	1.24810486

ZBTB7A	ZBTB8B	0.6577854	-0.59010416	-1.24788956	1.24788956
HLA-C	MFSD12	-0.60259694	0.6445654	1.24716234	1.24716234
KLF9	PRR14L	-0.6454763	0.6014702	1.2469465	1.2469465
SEPT11	IRF1	0.67388785	-0.5719302	-1.24581805	1.24581805
COPZ1	AP3S2	-0.7320422	0.5135248	1.245567	1.245567
SRPRB	MZF1	-0.5279685	0.7175736	1.2455421	1.2455421
SDC2	THBS3	0.7265176	-0.5186326	-1.2451502	1.2451502
QSOX1	KIF20A	-0.4270813	0.8177988	1.2448801	1.2448801
GSTP1	RACGAP1	-0.66346496	0.58140224	1.2448672	1.2448672
DTL	MRPL47	0.74591464	-0.49857715	-1.24449179	1.24449179
KCNK5	TOP3A	-0.6909144	0.553532	1.2444464	1.2444464
PDIA5	ARMC12	-0.77292997	0.4712695	1.24419947	1.24419947
RCC2	MAN2B2	-0.7497514	0.49442387	1.24417527	1.24417527
KANSL2	PDE7A	0.6435454	-0.59974563	-1.24329103	1.24329103
NFIA	TRIAP1	-0.65981907	0.5833804	1.24319947	1.24319947
ESRRG	SGCB	-0.5657875	0.6772725	1.24306	1.24306
SET	APOE	-0.5876586	0.65531695	1.24297555	1.24297555
PCNA	EPHA1	0.56205016	-0.6807435	-1.24279366	1.24279366
PTGER3	NAB2	0.57025623	-0.67234534	-1.24260157	1.24260157
NGFR	TIMM23	0.6069994	-0.63517964	-1.24217904	1.24217904
CSNK2A1	CD48	0.6353478	-0.6065509	-1.2418987	1.2418987
ESRRG	DLG1	-0.56595933	0.67561066	1.24156999	1.24156999
TBL1X	DCAF8	-0.5542635	0.6867638	1.2410273	1.2410273
EPS15	PSMB3	-0.70325464	0.5374246	1.24067924	1.24067924
MFAP3	TNFRSF21	-0.60977143	0.63054264	1.24031407	1.24031407
ZDHHC14	SDPR	0.62823665	-0.61187524	-1.24011189	1.24011189
LDLRAD4	TIMELESS	-0.6573043	0.5827976	1.2401019	1.2401019
KIF5B	LZTS1	0.73037106	-0.5088117	-1.23918276	1.23918276
NAALADL2	MSRB1	-0.56262267	0.6737894	1.23641207	1.23641207
CYR61	WISP1	0.6109724	-0.6254056	-1.236378	1.236378
MTFP1	SLC25A5	0.7654799	-0.47073284	-1.23621274	1.23621274
PDHB	ELK1	0.64272463	-0.59270114	-1.23542577	1.23542577
COPZ1	QKI	-0.63264346	0.60231376	1.23495722	1.23495722
ZHX2	NIPA2	-0.33735028	0.8975115	1.23486178	1.23486178
DIEXF	USP9X	-0.41137776	0.82324433	1.23462209	1.23462209
CTNNA1	ADAMTS3	-0.68176335	0.5528184	1.23458175	1.23458175
CCT3	DCAF7	-0.5380505	0.69643104	1.23448154	1.23448154
RBM17	CSNK2A1	-0.44787282	0.7865706	1.23444342	1.23444342
SSPN	STX6	-0.69966686	0.5341431	1.23380996	1.23380996
MUC1	SORL1	0.5787616	-0.6543253	-1.2330869	1.2330869
DCAF8	WDR46	-0.6283682	0.6044745	1.2328427	1.2328427
DPT	ARHGEF12	-0.8579405	0.37457585	1.23251635	1.23251635
CSPG4	SUSD1	-0.64370626	0.58862317	1.23232943	1.23232943
LZTS1	TNFAIP2	-0.52054703	0.7116318	1.23217883	1.23217883
MLH1	SPEF2	0.37149334	-0.86042136	-1.2319147	1.2319147
MRPL17	CPEB1	-0.8274026	0.40441838	1.23182098	1.23182098
PFDN4	CREB3L4	0.4406408	-0.79113483	-1.23177563	1.23177563
FOXM1	NDFIP2	0.7235189	-0.5082417	-1.2317606	1.2317606
NEGR1	PHF8	0.6168795	-0.6148601	-1.2317396	1.2317396
C1QTNF6	MRPL47	0.6093656	-0.62122524	-1.23059084	1.23059084
KCNRG	SLC25A13	0.46572876	-0.7648463	-1.23057506	1.23057506
PCNA	TMEM163	-0.42255417	0.8078609	1.23041507	1.23041507
SAP30L	XPO5	-0.63205475	0.59834296	1.23039771	1.23039771
WWTR1	AMOT	-0.59752774	0.63270485	1.23023259	1.23023259
CAST	XRCC5	-0.4013713	0.828773	1.2301443	1.2301443

DPT	THBS3	0.5290055	-0.7009276	-1.2299331	1.2299331
ESRRG	SSFA2	-0.58733183	0.64258385	1.22991568	1.22991568
CTGF	WISP1	0.68132305	-0.5482444	-1.22956745	1.22956745
BID	SSPN	-0.7223754	0.50648147	1.22885687	1.22885687
FANCI	FANCB	0.6176202	-0.6111999	-1.2288201	1.2288201
ACVR2A	MPV17L	-0.5101527	0.7182307	1.2283834	1.2283834
GFRA4	NFASC	0.8000866	-0.42806047	-1.22814707	1.22814707
MFAP3	PTPN21	-0.49757022	0.72960913	1.22717935	1.22717935
C1QBP	CHI3L2	0.6982884	-0.5285839	-1.2268723	1.2268723
ZBTB20	SOX4	0.6579753	-0.568618	-1.2265933	1.2265933
ZFP36L1	KIAA1033	0.82980144	-0.39626747	-1.22606891	1.22606891
ARHGEF12	PCIF1	0.56313974	-0.66209906	-1.2252388	1.2252388
NTRK2	GNG12	-0.81191343	0.41314188	1.22505531	1.22505531
ESRRG	RTN4	-0.5156167	0.709066	1.2246827	1.2246827
MPHOSPH6	TSPAN9	0.61509746	-0.60945016	-1.22454762	1.22454762
XPO1	PPP1CA	-0.39536154	0.8291399	1.22450144	1.22450144
DCN	NMNAT3	0.8556903	-0.36873174	-1.22442204	1.22442204
PPIL2	MIR503	-0.7858424	0.4385641	1.2244065	1.2244065
MAPKAP1	EIF4G3	0.63442063	-0.5898415	-1.22426213	1.22426213
SSBP2	PEX5	-0.55529904	0.668944	1.22424304	1.22424304
MCM6	EPHA1	0.58498955	-0.6390379	-1.22402745	1.22402745
XRCC5	THRB	-0.7427294	0.48075923	1.22348863	1.22348863
FOXJ2	LPIN1	0.3793529	-0.8439967	-1.2233496	1.2233496
KALRN	ENO3	-0.60300493	0.62026596	1.22327089	1.22327089
CSNK2A1	KCNQ4	-0.6339098	0.5891574	1.2230672	1.2230672
ITGB6	TNC	0.66467714	-0.5583196	-1.22299674	1.22299674
ESRRG	PRRC2A	0.6343329	-0.588512	-1.2228449	1.2228449
DNMT1	PCMTD1	-0.71268237	0.5099577	1.22264007	1.22264007
MED15	RACGAP1	-0.6363869	0.5859455	1.2223324	1.2223324
PRICKLE1	CD164	0.7149994	-0.50563186	-1.22063126	1.22063126
RUFY3	ZWILCH	-0.7970466	0.42337856	1.22042516	1.22042516
CLIP1	EIF4G3	0.6190065	-0.6013059	-1.2203124	1.2203124
ESRRG	PDZD2	-0.5786031	0.64139843	1.22000153	1.22000153
ALDH5A1	SLC2A5	0.56889266	-0.6504569	-1.21934956	1.21934956
TP53BP1	PRR14L	-0.6700975	0.5489637	1.2190612	1.2190612
MUC1	KCND3	0.5494348	-0.669583	-1.2190178	1.2190178
XRCC5	CPNE3	-0.647098	0.5718199	1.2189179	1.2189179
FAM96A	RBM17	-0.7590593	0.45921287	1.21827217	1.21827217
KANK1	SOX4	0.504389	-0.7138055	-1.2181945	1.2181945
BMP15	ZSCAN20	0.5589186	-0.65923357	-1.21815217	1.21815217
XRCC5	PDE4DIP	-0.73589355	0.4822489	1.21814245	1.21814245
ANKRD28	ZWILCH	-0.6711186	0.54566497	1.21678357	1.21678357
ZDHHC14	HGF	0.70396596	-0.5125947	-1.21656066	1.21656066
GET4	TCF3	0.88628805	-0.32980064	-1.21608869	1.21608869
PLA2G2A	ESRRG	0.42887336	-0.78685474	-1.2157281	1.2157281
ATP6V1G1	C10orf54	-0.61456835	0.60099983	1.21556818	1.21556818
ZNF608	KIAA0391	-0.46286684	0.75268555	1.21555239	1.21555239
MCM3	PTTG1	0.8143693	-0.400565	-1.2149343	1.2149343
NUDCD2	HLA-C	-0.7280905	0.48678458	1.21487508	1.21487508
DLG1	EIF4G3	0.7213695	-0.49307704	-1.21444654	1.21444654
UBC	STX6	-0.47045258	0.7437598	1.21421238	1.21421238
APC	PSMB3	-0.51573616	0.6981604	1.21389656	1.21389656
SHMT1	PGF	-0.73984903	0.473753	1.21360203	1.21360203
WTAP	UCK2	0.8057297	-0.40731123	-1.21304093	1.21304093
NVL	HLA-C	-0.65263045	0.56034464	1.21297509	1.21297509

HLF	ZDHHC14	0.73020333	-0.48276445	-1.21296778	1.21296778
FOXN3	PI3	-0.8559093	0.3567367	1.212646	1.212646
MVP	FOS	0.53318423	-0.67943096	-1.21261519	1.21261519
BID	RNASE4	-0.67543596	0.53686446	1.21230042	1.21230042
STX6	SLC43A2	0.74688107	-0.46477836	-1.21165943	1.21165943
EPS15	NPC1	-0.4847582	0.7267547	1.2115129	1.2115129
UBC	MCM3	-0.5083812	0.7026548	1.211036	1.211036
DKC1	RBM17	-0.56339586	0.6475767	1.21097256	1.21097256
LDLRAD4	UBE2B	0.8111707	-0.39975074	-1.21092144	1.21092144
TMEM254	SNRNP48	-0.8301726	0.37991786	1.21009046	1.21009046
LPCAT1	AUP1	-0.43902025	0.7709513	1.20997155	1.20997155
CA12	DSG2	-0.6232619	0.585738	1.2089999	1.2089999
QSOX1	PDZRN3	0.5621109	-0.64637685	-1.20848775	1.20848775
RELB	SLC25A5	0.46140906	-0.74706733	-1.20847639	1.20847639
CTGF	ARPP21	-0.7496509	0.45866063	1.20831153	1.20831153
SERPINA1	PI3	0.6342295	-0.5737411	-1.2079706	1.2079706
ESRRG	PHF10	-0.5886924	0.6190434	1.2077358	1.2077358
MED31	RRN3	-0.5567875	0.65089005	1.20767755	1.20767755
SGCB	RACGAP1	-0.7941288	0.41353214	1.20766094	1.20766094
DNMT1	RAB27A	0.8259695	-0.38159367	-1.20756317	1.20756317
BID	ZHX3	-0.6931899	0.5142995	1.2074894	1.2074894
CAP2	MRPS22	-0.6373016	0.5699375	1.2072391	1.2072391
MYOC	LY75	0.6175837	-0.5896173	-1.207201	1.207201
MAPK10	MOCOS	-0.64155585	0.5656176	1.20717345	1.20717345
NPM1	PPA1	0.7003791	-0.50677365	-1.20715275	1.20715275
EGR1	IGFBP4	0.48819414	-0.7187546	-1.20694874	1.20694874
UBE2B	RTN4	0.8929009	-0.31329998	-1.20620088	1.20620088
PCNA	HLA-C	-0.54737633	0.6588227	1.20619903	1.20619903
PGRMC2	SOX4	0.37783256	-0.82830137	-1.20613393	1.20613393
ZNF148	HDAC1	-0.5784274	0.6275175	1.2059449	1.2059449
GMPS	CSTF3	0.79270095	-0.41226947	-1.20497042	1.20497042
PGF	IRF2BP2	0.70994025	-0.4949839	-1.20492415	1.20492415
CSF3R	FNDC1	-0.6978643	0.50656456	1.20442886	1.20442886
HDAC11	YARS	-0.65049446	0.5537837	1.20427816	1.20427816
GALNT2	CST1	0.46672082	-0.73749727	-1.20421809	1.20421809
IGF1	AP3M1	-0.7526485	0.45096508	1.20361358	1.20361358
ZDHHC14	SRI	0.6806784	-0.5218747	-1.2025531	1.2025531
APC	MED31	-0.5342431	0.66795975	1.20220285	1.20220285
BYSL	HDAC1	0.7240958	-0.47808442	-1.20218022	1.20218022
TBL2	TM4SF18	-0.56094426	0.6408546	1.20179886	1.20179886
CCDC3	POPDC3	-0.43916762	0.7625941	1.20176172	1.20176172
DHX30	HLA-C	-0.34993175	0.8517271	1.20165885	1.20165885
TRA2B	CHD1L	0.37040603	-0.83092296	-1.20132899	1.20132899
SF3B3	ZNF467	-0.4807722	0.7202178	1.20099	1.20099
BID	AMIGO1	-0.5875342	0.61321664	1.20075084	1.20075084
SLC7A1	INIP	0.54728913	-0.6533572	-1.20064633	1.20064633
RMI1	SESN1	-0.43570518	0.76488334	1.20058852	1.20058852
ACVR2A	ORC5	-0.50421166	0.6960588	1.20027046	1.20027046
SSPN	SOX4	0.4918691	-0.7081314	-1.2000005	1.2000005
ZDHHC14	CRTAP	0.48250887	-0.71682394	-1.19933281	1.19933281
HLA-G	FAM216A	-0.49025768	0.70902824	1.19928592	1.19928592
EPS15	C3orf17	-0.35723168	0.8416689	1.19890058	1.19890058
XRCC5	TM9SF3	-0.49753264	0.7011743	1.19870694	1.19870694
EMC6	HOXA7	-0.6081232	0.59016865	1.19829185	1.19829185
ARNTL	MLXIP	-0.6965945	0.5013171	1.1979116	1.1979116

CSF3R	BMP1	-0.58471745	0.61303264	1.19775009	1.19775009
KARS	PDZRN3	-0.52446103	0.6730614	1.19752243	1.19752243
KLF12	SRPRB	0.37137428	-0.82602894	-1.19740322	1.19740322
LOC145783	PPIL2	-0.54318017	0.65399486	1.19717503	1.19717503
WWTR1	SLC39A6	-0.3537459	0.8429494	1.1966953	1.1966953
PALLD	SLC39A6	-0.51257986	0.68365586	1.19623572	1.19623572
KARS	PDE4DIP	-0.591933	0.6040287	1.1959617	1.1959617
LIPF	VAMP3	-0.54262584	0.65315574	1.19578158	1.19578158
SH3PXD2A	TAB2	-0.74847454	0.44730538	1.19577992	1.19577992
ANKRD28	MTHFD1L	-0.39404166	0.801708	1.19574966	1.19574966
ESRRG	AGFG1	-0.6163158	0.5793373	1.1956531	1.1956531
SNCG	MCM3	0.4127377	-0.78263354	-1.19537124	1.19537124
RBMS3	SOX4	0.57174957	-0.6236116	-1.19536117	1.19536117
XPO1	POSTN	-0.5100051	0.68519276	1.19519786	1.19519786
ESRRG	SESTD1	-0.5691449	0.626018	1.1951629	1.1951629
HLF	MRPS22	-0.5320048	0.6631159	1.1951207	1.1951207
ADAM10	PFDN4	0.5455552	-0.6489095	-1.1944647	1.1944647
DCN	LTBP4	0.9358703	-0.25856522	-1.19443552	1.19443552
KARS	NFIA	-0.5050513	0.68935597	1.19440727	1.19440727
MCM3	SPAG5	0.73816586	-0.4560622	-1.19422806	1.19422806
ADGRA2	MMAB	-0.39438456	0.7996576	1.19404216	1.19404216
ZDHHC14	SH3D19	0.5728191	-0.62102103	-1.19384013	1.19384013
IGF2R	NUDT21	-0.4874406	0.70636874	1.19380934	1.19380934
XRCC5	MOB3B	-0.54961085	0.6441632	1.19377405	1.19377405
MCM4	MCM3	0.86193496	-0.33182037	-1.19375533	1.19375533
ESRRG	MMD	-0.51475394	0.6789423	1.19369624	1.19369624
SLC39A6	PHF8	0.4601628	-0.7332699	-1.1934327	1.1934327
MTHFD1L	ABCA1	-0.6510945	0.5420013	1.1930958	1.1930958
DDX6	DDX54	-0.61319894	0.57970726	1.1929062	1.1929062
NEGR1	SEMA4B	0.5695496	-0.6230023	-1.1925519	1.1925519
HSP90AA1	C1QTNF6	0.51867145	-0.6738759	-1.19254735	1.19254735
RAB8B	TSPAN9	0.8186535	-0.3737489	-1.1924024	1.1924024
POPDC3	FEZ2	-0.53356165	0.6587838	1.19234545	1.19234545
BID	BCL2L1	0.6780408	-0.51420134	-1.19224214	1.19224214
PCNA	QKI	-0.6723786	0.51972324	1.19210184	1.19210184
KALRN	PNPT1	-0.33943245	0.8525851	1.19201755	1.19201755
ZFP36L1	ABCA1	0.41938925	-0.7720076	-1.19139685	1.19139685
UNC5C	FOXO3	-0.46749675	0.72376174	1.19125849	1.19125849
EIF6	DNAJC15	-0.46461016	0.72606474	1.1906749	1.1906749
VLDLR	HSD17B6	0.8667801	-0.32350552	-1.19028562	1.19028562
RSPO3	C10orf90	-0.8119126	0.37814882	1.19006142	1.19006142
COL5A1	FCN1	-0.34237522	0.8476598	1.19003502	1.19003502
HDAC1	IL2	0.6584248	-0.5314071	-1.1898319	1.1898319
DDB2	DPH7	-0.6073336	0.5823325	1.1896661	1.1896661
PALLD	NEGR1	-0.5843992	0.6051836	1.1895828	1.1895828
PTPN2	CTNNA1	-0.63019276	0.55935156	1.18954432	1.18954432
TOP2A	EXOSC8	0.60555494	-0.5838029	-1.18935784	1.18935784
RSPO3	MZF1	0.62120795	-0.5679554	-1.18916335	1.18916335
COLGALT1	DCAF8	-0.3894305	0.79959	1.1890205	1.1890205
PDIA5	MZF1	-0.3056723	0.88283706	1.18850936	1.18850936
SS18L2	DPP3	0.83933985	-0.3491486	-1.18848845	1.18848845
MGAT5	ADGRV1	-0.73265886	0.4553949	1.18805376	1.18805376
LOC145783	PDE7A	0.6139575	-0.5739444	-1.1879019	1.1879019
TNC	FAT3	-0.3740908	0.81351674	1.18760754	1.18760754
PSMB2	CRTAP	-0.43968195	0.74788505	1.187567	1.187567

GAPDH	RBM17	-0.79458725	0.3927499	1.18733715	1.18733715
DHRS12	MRPL47	-0.73969173	0.4476082	1.18729993	1.18729993
SFN	GGCT	0.58096105	-0.6060224	-1.18698345	1.18698345
RAB35	AHI1	0.6014385	-0.58553165	-1.18697015	1.18697015
ZBTB44	SPEF2	-0.5267331	0.66023093	1.18696403	1.18696403
TRIM27	MZF1	-0.41366524	0.77324176	1.186907	1.186907
OPRK1	FOXO3	-0.52247095	0.6642956	1.18676655	1.18676655
NEGR1	MXRA7	-0.6327778	0.5537938	1.1865716	1.1865716
SSB	LRP8	0.5887686	-0.5974073	-1.1861759	1.1861759
LAMA1	STK36	-0.48783937	0.69827884	1.18611821	1.18611821
SFN	DKC1	0.7683763	-0.41756693	-1.18594323	1.18594323
SERBP1	SPG20	-0.57173276	0.61413366	1.18586642	1.18586642
PAX2	UNC5C	0.557459	-0.6281025	-1.1855615	1.1855615
NOL10	SPEF2	0.37817174	-0.8073772	-1.18554894	1.18554894
GALNT2	EBF1	-0.6575077	0.5279767	1.1854844	1.1854844
PDE4DIP	MFAP3	-0.42271295	0.7626399	1.18535285	1.18535285
GTF2I	SOX4	0.71291053	-0.47237834	-1.18528887	1.18528887
TUFM	HLA-C	-0.5745392	0.6098776	1.1844168	1.1844168
TYRO3	CBLN4	0.68476135	-0.4995956	-1.18435695	1.18435695
MUTYH	SLAMF1	0.5651028	-0.6191551	-1.1842579	1.1842579
PPIL2	STIM2	-0.43738225	0.7465167	1.18389895	1.18389895
LARP6	SOX4	0.5251476	-0.6587213	-1.1838689	1.1838689
ARNTL	OSMR	-0.5711806	0.6124866	1.1836672	1.1836672
PI3	JDP2	-0.6969975	0.48662752	1.18362502	1.18362502
PSMA4	TCEA3	-0.72485185	0.45848715	1.183339	1.183339
TEC	PTPN21	0.62572354	-0.5575818	-1.18330534	1.18330534
TRIAP1	PCED1B	-0.65734273	0.5259372	1.18327993	1.18327993
DCUN1D5	KLF12	0.31545037	-0.86744386	-1.18289423	1.18289423
SCML2	PER3	-0.60513276	0.5776438	1.18277656	1.18277656
MCM3	ZBTB20	-0.8913736	0.29104275	1.18241635	1.18241635
EFEMP2	NID2	0.61518186	-0.56723356	-1.18241542	1.18241542
PTDSS1	INF2	0.5415107	-0.64086986	-1.18238056	1.18238056
GSTM3	URI1	-0.6261838	0.5561902	1.182374	1.182374
SCRN1	BMP1	0.7231019	-0.45920885	-1.18231075	1.18231075
AGTRAP	ENTHD2	-0.3687012	0.81358516	1.18228636	1.18228636
ZNF608	NAT10	-0.4279866	0.7542589	1.1822455	1.1822455
SNTB2	THBS3	0.85635376	-0.32570976	-1.18206352	1.18206352
ITGB6	OGFOD2	-0.7726727	0.40902522	1.18169792	1.18169792
AGPAT5	CSPG5	-0.40926233	0.7721068	1.18136913	1.18136913
VCAM1	STX7	0.5451238	-0.6361771	-1.1813009	1.1813009
ACVR2A	RAB13	0.42153862	-0.7596195	-1.18115812	1.18115812
MANF	EPHX2	-0.7676973	0.41341725	1.18111455	1.18111455
EZH2	EBP	0.56592566	-0.61484826	-1.18077392	1.18077392
ACACA	FAM43A	-0.5872639	0.59336823	1.18063213	1.18063213
TRIO	ARHGEF37	-0.75964236	0.42085308	1.18049544	1.18049544
SFN	SERBP1	0.71890205	-0.46149254	-1.18039459	1.18039459
DDX20	SUFU	-0.5386305	0.6417277	1.1803582	1.1803582
TRMT13	AGPAT5	-0.53315717	0.6471424	1.18029957	1.18029957
TOP2A	TUSC3	0.56902194	-0.61104226	-1.1800642	1.1800642
SFN	PSMB3	0.6704302	-0.50959593	-1.18002613	1.18002613
TAOK3	GALNT2	-0.6665475	0.5133153	1.1798628	1.1798628
ESRRG	LDLRAD4	-0.5257557	0.65377116	1.17952686	1.17952686
SF3B3	SLC25A29	-0.5854143	0.59371686	1.17913116	1.17913116
ABI2	NOL8	0.79753774	-0.38143715	-1.17897489	1.17897489
KANSL1	FOXK2	-0.6709261	0.50779504	1.17872114	1.17872114

MFAP3	ADPGK	-0.4005727	0.7781077	1.1786804	1.1786804
KALRN	SOBP	0.51905924	-0.65922225	-1.17828149	1.17828149
KIRREL	MTHFD1L	-0.37114424	0.8070723	1.17821654	1.17821654
PJA2	MALT1	-0.8521082	0.3259926	1.1781008	1.1781008
ITPA	BECN1	0.6645461	-0.51349026	-1.17803636	1.17803636
MAPK10	PRICKLE1	0.78494626	-0.39296487	-1.17791113	1.17791113
UBE2B	NDN	0.7792534	-0.3983822	-1.1776356	1.1776356
C12orf76	PGS1	0.45741364	-0.7200913	-1.17750494	1.17750494
SSBP2	SH3PXD2A	-0.6422345	0.53472066	1.17695516	1.17695516
RUVBL2	MCM4	0.8200741	-0.3568373	-1.1769114	1.1769114
SRPRB	SP3	-0.4636295	0.7131338	1.1767633	1.1767633
COL15A1	SORL1	-0.49436283	0.6823311	1.17669393	1.17669393
EBF1	SLIRP	-0.6768384	0.49922705	1.17606545	1.17606545
APC	TRIM27	-0.56901246	0.6068926	1.17590506	1.17590506
CBX4	ARHGEF2	-0.3969752	0.77885234	1.17582754	1.17582754
POC1A	IDNK	-0.73857385	0.4369997	1.17557355	1.17557355
AK2	SMOX	0.4398321	-0.73568743	-1.17551953	1.17551953
SNRPC	VAMP4	-0.61024857	0.5652271	1.17547567	1.17547567
C4A	VANGL1	-0.54589427	0.629502	1.17539627	1.17539627
MCM6	QKI	-0.7469317	0.4281812	1.1751129	1.1751129
DIAPH1	SOX4	-0.622739	0.5522068	1.1749458	1.1749458
HPS6	ATP6V0E2	-0.74057734	0.43431586	1.1748932	1.1748932
XPO1	MLXIP	-0.4714365	0.7031318	1.1745683	1.1745683
PDZRN3	MRPS22	-0.463729	0.71073925	1.17446825	1.17446825
MED14	HLA-C	-0.46427476	0.7101785	1.17445326	1.17445326
TRAF3IP1	GALNT2	-0.56073445	0.61346555	1.1742	1.1742
UBC	PCNA	-0.5420241	0.6319789	1.174003	1.174003
ACLY	DUSP1	-0.6097661	0.5639476	1.1737137	1.1737137
QKI	PNPT1	-0.6164963	0.55719644	1.17369274	1.17369274
GNAL	CERS5	0.70459515	-0.46894807	-1.17354322	1.17354322
XPO1	AMMECR1	0.42881215	-0.74465036	-1.17346251	1.17346251
PFDN4	USP3	0.60700786	-0.56634647	-1.17335433	1.17335433
ZNF608	MFSD12	-0.668559	0.50416416	1.17272316	1.17272316
PPA1	DHRS12	-0.48667112	0.68595815	1.17262927	1.17262927
TRIM25	LARS2	0.7458449	-0.4267514	-1.1725963	1.1725963
PDIA4	BCL2L1	0.41521782	-0.7573309	-1.17254872	1.17254872
DCAF8	SF3B3	-0.72542244	0.44709456	1.172517	1.172517
FCF1	ZSCAN20	0.5641629	-0.60831195	-1.17247485	1.17247485
TMEM163	ATP6V0E2	0.81684154	-0.35548884	-1.17233038	1.17233038
EPHX2	NOL10	-0.43456358	0.73747164	1.17203522	1.17203522
STAT3	JUNB	0.62601656	-0.5454114	-1.17142796	1.17142796
ATP1B3	SLC24A4	-0.77379423	0.39748442	1.17127865	1.17127865
ADRA1B	GMFG	0.47398096	-0.6970364	-1.17101736	1.17101736
MOCOS	TCF3	0.6137914	-0.55696535	-1.17075675	1.17075675
MCM3	CHD2	-0.6058033	0.5646926	1.1704959	1.1704959
MAP1B	CD40	0.49613935	-0.67409074	-1.17023009	1.17023009
KALRN	ZWILCH	-0.46938023	0.7008182	1.17019843	1.17019843
KLF12	NIPA2	0.31777748	-0.8519454	-1.16972288	1.16972288
PELP1	PHF8	0.6664695	-0.5032513	-1.1697208	1.1697208
GAPDH	FAT3	-0.6191597	0.55053127	1.16969097	1.16969097
GNAQ	RACGAP1	-0.53943247	0.63008803	1.1695205	1.1695205
MFAP3	AACS	-0.6338949	0.53551376	1.16940866	1.16940866
XPO1	IPO11	-0.6258964	0.54343957	1.16933597	1.16933597
KIRREL	NOL8	-0.37321374	0.79609835	1.16931209	1.16931209
KARS	LMNB2	0.79333794	-0.37594557	-1.16928351	1.16928351

ADA	ENTHD2	-0.65718454	0.51197	1.16915454	1.16915454
KSR1	CCT3	-0.55880284	0.6099007	1.16870354	1.16870354
ENOSF1	VPS16	-0.54106086	0.6274384	1.16849926	1.16849926
IGF2R	ACTN3	0.3440902	-0.82413274	-1.16822294	1.16822294
RUFY3	FANCI	-0.72921365	0.4386647	1.16787835	1.16787835
IPO11	RNF130	-0.5468497	0.62102044	1.16787014	1.16787014
MFAP3	CCDC90B	-0.57435095	0.59333926	1.16769021	1.16769021
SFN	RACGAP1	0.6797043	-0.48782387	-1.16752817	1.16752817
AK2	FOXJ2	0.45430356	-0.7130226	-1.16732616	1.16732616
HCRTR2	PDE7A	-0.5402106	0.62684065	1.16705125	1.16705125
FANCA	CDK1	0.7449918	-0.42201433	-1.16700613	1.16700613
APC	MAP4K4	-0.6490401	0.51782197	1.16686207	1.16686207
EFCAB14	GET4	-0.37335375	0.79350424	1.16685799	1.16685799
WRAP53	PRR11	0.8110003	-0.3557268	-1.1667271	1.1667271
USP4	ZNF276	-0.7989136	0.3675607	1.1664743	1.1664743
THRB	PSMA4	-0.72149396	0.44486308	1.16635704	1.16635704
PCNA	CDKN3	0.8788936	-0.28739098	-1.16628458	1.16628458
CASP8	DIEXF	0.5350598	-0.6312001	-1.1662599	1.1662599
EPS15	TXNL1	-0.37063757	0.7955057	1.16614327	1.16614327
PFDN4	EXOSC8	0.5361857	-0.6293977	-1.1655834	1.1655834
BZW2	TEFM	-0.6958247	0.4695608	1.1653855	1.1653855
CDK6	CDK10	0.37102935	-0.79431385	-1.1653432	1.1653432
MFAP3	TRMT13	-0.69963247	0.46569648	1.16532895	1.16532895
EIF4E3	MRPL47	-0.43915656	0.7259841	1.16514066	1.16514066
ACVR2A	WDR27	0.73194844	-0.43315393	-1.16510237	1.16510237
CSF3R	CERS5	-0.72489387	0.44004363	1.1649375	1.1649375
MGAT5	KALRN	0.42668894	-0.73813635	-1.16482529	1.16482529
NAALADL2	FOPNL	-0.54714906	0.617451	1.16460006	1.16460006
QDPR	QSOX1	0.6888171	-0.4756734	-1.1644905	1.1644905
CALD1	COL3A1	0.7369608	-0.42747268	-1.16443348	1.16443348
SAA4	PALMD	-0.5643909	0.59980404	1.16419494	1.16419494
CAB39L	YME1L1	0.5409567	-0.623102	-1.1640587	1.1640587
FOXJ2	PDE7A	-0.51072663	0.65251195	1.16323858	1.16323858
MFAP3	PPM1A	-0.6083808	0.5548258	1.1632066	1.1632066
NEGR1	CEP112	-0.5783376	0.5848478	1.1631854	1.1631854
SPIRE2	LCMT2	0.5783275	-0.58478874	-1.16311624	1.16311624
KLF12	MED31	0.4543897	-0.70844424	-1.16283394	1.16283394
CAST	RBM45	-0.34418926	0.8180549	1.16224416	1.16224416
ITGB1	COL6A3	0.51354903	-0.64867896	-1.16222799	1.16222799
MCM5	MCM4	0.73123837	-0.43095213	-1.1621905	1.1621905
APC	PTDSS1	-0.5093927	0.6523878	1.1617805	1.1617805
FAM96A	TSPAN9	0.5425861	-0.6191251	-1.1617112	1.1617112
PPA1	S100A16	0.60200536	-0.5594825	-1.16148786	1.16148786
GMPS	CDCA7	0.917208	-0.24426004	-1.16146804	1.16146804
ZNF385D	LBP	0.7586787	-0.40264794	-1.16132664	1.16132664
HDAC11	FGFR1OP2	-0.63075256	0.53035235	1.16110491	1.16110491
C10orf54	KANK4	0.740663	-0.42036074	-1.16102374	1.16102374
DDB2	ARPC1B	-0.35592726	0.80492336	1.16085062	1.16085062
ADAMTS2	ENOSF1	-0.5664575	0.59430796	1.16076546	1.16076546
ALDH3A2	MFAP3	-0.64425015	0.5164965	1.16074665	1.16074665
USP9X	MALT1	-0.80719745	0.35338166	1.16057911	1.16057911
EPS15	MRPL47	-0.79857576	0.3619188	1.16049456	1.16049456
CSTF3	TOP2A	0.6826116	-0.47755593	-1.16016753	1.16016753
APOE	SPINK1	0.68262583	-0.47749218	-1.16011801	1.16011801
PDZRN3	CBLN2	0.40470254	-0.75532603	-1.16002857	1.16002857

GEMIN4	ZNF202	-0.4898532	0.6701468	1.16	1.16
EIF4G3	URB2	0.84688807	-0.31303042	-1.15991849	1.15991849
RAE1	NXF1	-0.3099789	0.84992075	1.15989965	1.15989965
PCDH9	SLC25A29	0.52988946	-0.6300068	-1.15989626	1.15989626
ESRRG	APOD	-0.6827962	0.47703657	1.15983277	1.15983277
POSTN	NUDCD2	-0.4658458	0.6939619	1.1598077	1.1598077
MCM3	OPTN	-0.511448	0.648262	1.15971	1.15971
PPP1R1B	CDK5	-0.68484825	0.4748244	1.15967265	1.15967265
ARHGEF12	EPDR1	-0.72103983	0.43822515	1.15926498	1.15926498
ESRRG	MLC1	0.71634066	-0.4426236	-1.15896426	1.15896426
QSOX1	RTN3	0.5793108	-0.57962424	-1.15893504	1.15893504
SET	RANGAP1	0.6226386	-0.5360075	-1.1586461	1.1586461
GNAQ	AGPAT5	-0.46876568	0.68987596	1.15864164	1.15864164
CCT3	PFDN4	0.41293752	-0.7455363	-1.15847382	1.15847382
MFAP3	BMP15	0.29338932	-0.86495835	-1.15834767	1.15834767
PCNA	KIF11	0.86782664	-0.29046902	-1.15829566	1.15829566
PPP1CA	EGR1	0.46060142	-0.69741565	-1.15801707	1.15801707
KCNAB1	TIMELESS	-0.74853075	0.4092195	1.15775025	1.15775025
CSNK2A1	MAP4	-0.59042674	0.5670053	1.15743204	1.15743204
PCNA	ITCH	-0.64811224	0.50884974	1.15696198	1.15696198
TRIAP1	DHRS12	-0.7613323	0.3954438	1.1567761	1.1567761
CFH	LEPR	0.55297995	-0.6037897	-1.15676965	1.15676965
ARNTL	C12orf49	-0.43920115	0.7172291	1.15643025	1.15643025
ENO3	CST2	0.5254184	-0.6307058	-1.1561242	1.1561242
PPCDC	ZC3H3	-0.31904677	0.83617926	1.15522603	1.15522603
SRGAP1	TM4SF18	-0.47496065	0.6801488	1.15510945	1.15510945
MMS19	RAB35	-0.7690072	0.38565513	1.15466233	1.15466233
ALAD	GALNT2	-0.48295513	0.67143	1.15438513	1.15438513
SRSF1	NXF1	-0.57557195	0.5786942	1.15426615	1.15426615
EIF4A1	CASP5	0.4744811	-0.67943	-1.1539111	1.1539111
CAP2	NAB2	0.55788994	-0.595908	-1.15379794	1.15379794
GSTP1	OLMALINC	-0.57692623	0.5763334	1.15325963	1.15325963
STXBP6	OSBPL7	0.47326636	-0.67992336	-1.15318972	1.15318972
MPPED2	TIMELESS	-0.4121234	0.74092376	1.15304716	1.15304716
ESRRG	MID2	-0.45287892	0.7001661	1.15304502	1.15304502
TOP2A	HDAC1	0.72864985	-0.424231	-1.15288085	1.15288085
RNF25	PHF8	-0.6656287	0.48663554	1.15226424	1.15226424
ADAMTS9	FAT3	-0.615138	0.5369323	1.1520703	1.1520703
COPZ1	KATNAL1	-0.566457	0.58545107	1.15190807	1.15190807
NAP1L1	SERBP1	-0.44640467	0.7054672	1.15187187	1.15187187
GSTP1	ZWINT	-0.5672679	0.58431834	1.15158624	1.15158624
IPO11	GALNS	-0.50724787	0.64428645	1.15153432	1.15153432
LDLRAD4	GET4	-0.57808375	0.5731146	1.15119835	1.15119835
MED31	SLC27A3	-0.3807501	0.7702062	1.1509563	1.1509563
PARK2	PPA1	-0.41746423	0.732859	1.15032323	1.15032323
RCC2	HLA-B	-0.53295356	0.61733496	1.15028852	1.15028852
SOX4	DPP3	-0.68549836	0.4647121	1.15021046	1.15021046
KCNAB1	COL1A1	0.5467718	-0.60329264	-1.15006444	1.15006444
XPO5	PFDN4	0.54854584	-0.60141784	-1.14996368	1.14996368
USP4	RAB13	0.75192463	-0.397662	-1.14958663	1.14958663
APOE	RMI1	-0.66333586	0.4862168	1.14955266	1.14955266
ALDH4A1	DFNB31	0.4930366	-0.6560589	-1.1490955	1.1490955
XRCC5	RGS5	-0.6820114	0.4670268	1.1490382	1.1490382
SRSF1	RBM17	-0.55379677	0.59519684	1.14899361	1.14899361
HDAC11	BID	-0.73573256	0.4132407	1.14897326	1.14897326

RNF24	EGR1	0.6370687	-0.5118646	-1.1489333	1.1489333
GSTM4	ZNF358	0.59605324	-0.5525411	-1.14859434	1.14859434
NEGR1	HELLS	0.58985794	-0.55873114	-1.14858908	1.14858908
RDH11	ARHGEF2	-0.5976784	0.5508427	1.1485211	1.1485211
EIF5	ETNPPL	0.5874218	-0.56108534	-1.14850714	1.14850714
AMIGO1	CBLN2	0.6346142	-0.51389277	-1.14850697	1.14850697
GRB14	QSOX1	0.4746355	-0.6737962	-1.1484317	1.1484317
XXYLT1	B4GALT6	-0.50606215	0.642363	1.14842515	1.14842515
EPHX2	ZNF202	-0.50562286	0.64274395	1.14836681	1.14836681
SERBP1	EPHA1	0.65204304	-0.4961538	-1.14819684	1.14819684
PDE4DIP	QSOX1	0.57057023	-0.5774797	-1.14804993	1.14804993
PCDH9	SOX4	0.5171338	-0.6307498	-1.1478836	1.1478836
RBM17	SS18L2	-0.7839526	0.36388013	1.14783273	1.14783273
MMD	PRICKLE1	0.43207446	-0.71557486	-1.14764932	1.14764932
STXBP6	XPNPEP1	0.44023138	-0.70739335	-1.14762473	1.14762473
MCM3	HLA-B	-0.35771003	0.78977287	1.1474829	1.1474829
ZBTB10	SOBP	0.7882373	-0.35916406	-1.14740136	1.14740136
B2M	MED14	-0.4739867	0.6733753	1.147362	1.147362
SAP30L	STAT3	-0.48826304	0.6587151	1.14697814	1.14697814
STX8	RACGAP1	-0.72435915	0.4225021	1.14686125	1.14686125
SNRPB2	PDE7A	0.6246586	-0.5220099	-1.1466685	1.1466685
SLC39A1	FOS	0.6978758	-0.44874978	-1.14662558	1.14662558
UNC5C	IL15RA	-0.39761215	0.74876374	1.14637589	1.14637589
KIF13A	GSTM5	0.78473055	-0.36142126	-1.14615181	1.14615181
EPS15	GAPDH	-0.73043734	0.4155373	1.14597464	1.14597464
TMEM144	MED15	-0.6635963	0.48223728	1.14583358	1.14583358
CEP68	TMEM229B	-0.5993074	0.5464487	1.1457561	1.1457561
ZNF467	DNAJC9	-0.557622	0.58764917	1.14527117	1.14527117
COL3A1	SLC25A13	-0.5375969	0.60761285	1.14520975	1.14520975
ARPC5L	CSRP2BP	-0.65855473	0.48565644	1.14421117	1.14421117
LOC145783	KIAA0232	-0.5342275	0.60994065	1.14416815	1.14416815
NFIA	PSMA4	-0.6866289	0.4572912	1.1439201	1.1439201
GAPDH	NNMT	0.65054226	-0.49321216	-1.14375442	1.14375442
PCNA	EBF1	-0.90139437	0.24223669	1.14363106	1.14363106
CCT6A	PFDN4	0.5090917	-0.63450265	-1.14359435	1.14359435
RARA	ARNTL	-0.6977274	0.445814	1.1435414	1.1435414
RGS5	C1QTNF3	0.6481938	-0.49470606	-1.14289986	1.14289986
AUP1	TEAD4	-0.4214954	0.72132355	1.14281895	1.14281895
LMO3	PRUNE2	0.4290495	-0.7137661	-1.1428156	1.1428156
IFNAR2	CUX1	-0.3026688	0.83997107	1.14263987	1.14263987
LAMA1	FAT3	-0.7019609	0.44064045	1.14260135	1.14260135
AKAP10	POU2F2	0.29777786	-0.8446985	-1.14247636	1.14247636
ARNTL	PHLDB2	0.5701411	-0.57225686	-1.14239796	1.14239796
LMO3	DPAGT1	-0.8320947	0.31029812	1.14239282	1.14239282
ITGB5	PDLIM4	0.46718037	-0.6751796	-1.14235997	1.14235997
EPS15	EIF2B2	-0.4028363	0.73950815	1.14234445	1.14234445
LDLRAD4	MFAP3	-0.3023387	0.8399222	1.1422609	1.1422609
IP6K2	IRF9	-0.4166994	0.7255336	1.142233	1.142233
HSD17B1	HSD11B1	-0.5437215	0.59811795	1.14183945	1.14183945
ADAM10	CTNNA1	-0.52761173	0.6141549	1.14176663	1.14176663
SLC7A2	COPRS	0.47990996	-0.6618161	-1.14172606	1.14172606
GSTM1	GSTM5	0.8144388	-0.32727993	-1.14171873	1.14171873
PARK2	PTPN4	-0.5985422	0.54295677	1.14149897	1.14149897
CXCL2	STAT3	0.6000699	-0.5413514	-1.1414213	1.1414213
AIDA	VPS16	0.6559119	-0.48493427	-1.14084617	1.14084617

FAT3	DDX39A	-0.6319506	0.5087327	1.1406833	1.1406833
PIK3CA	COL5A1	0.53357166	-0.6070694	-1.14064106	1.14064106
QKI	DIEXF	-0.55084234	0.58976936	1.1406117	1.1406117
YARS	MCM4	0.69372547	-0.44680056	-1.14052603	1.14052603
RERGL	NUDCD2	-0.6100757	0.53041106	1.14048676	1.14048676
COPZ1	TM4SF18	-0.39079294	0.74965733	1.14045027	1.14045027
AIDA	CERS5	0.47936916	-0.6609588	-1.14032796	1.14032796
CYP2F1	CXCL1	-0.82462996	0.3156184	1.14024836	1.14024836
CLIP1	GPC1	0.7214304	-0.4187191	-1.1401495	1.1401495
PRUNE2	MADD	-0.5536811	0.5864534	1.1401345	1.1401345
URI1	FBXO9	0.7017003	-0.43823206	-1.13993236	1.13993236
GNAL	ARPP21	-0.5579378	0.5816815	1.1396193	1.1396193
GPC1	PINK1	0.54215986	-0.59729266	-1.13945252	1.13945252
ZNF385D	ZNF148	-0.52181053	0.6176165	1.13942703	1.13942703
HDAC11	SPCS3	-0.7052256	0.4339452	1.1391708	1.1391708
SNRPB2	SNTB1	-0.6307162	0.508367	1.1390832	1.1390832
UBC	HDAC1	-0.634058	0.5047849	1.1388429	1.1388429
ZDHHC14	PGRMC2	0.49820885	-0.6403781	-1.13858695	1.13858695
ASB1	DNAJC9	-0.3834596	0.7550266	1.1384862	1.1384862
NFATC2IP	BMP1	-0.4686302	0.6697845	1.1384147	1.1384147
TRIT1	KALRN	-0.3465579	0.79155225	1.13811015	1.13811015
CASP6	ZNF385D	-0.74790895	0.38950187	1.13741082	1.13741082
DFNB31	TRNP1	-0.416827	0.72051907	1.13734607	1.13734607
CYBRD1	AGPAT5	-0.59178406	0.5455309	1.13731496	1.13731496
KARS	AHNAK	-0.78242654	0.3547428	1.13716934	1.13716934
CTNNA1	TRO	-0.65859157	0.47834104	1.13693261	1.13693261
DBI	PRUNE2	0.6851332	-0.45150426	-1.13663746	1.13663746
EPS15	CCT3	-0.65270823	0.48376718	1.13647541	1.13647541
PDE4DIP	COPZ1	-0.6099899	0.52645797	1.13644787	1.13644787
KDR	ZSCAN20	-0.65952885	0.47688952	1.13641837	1.13641837
DKC1	IGF2R	-0.7505311	0.38575688	1.13628798	1.13628798
PDE9A	LAGE3	-0.7517738	0.38443324	1.13620704	1.13620704
UCK2	XXYLT1	0.81681436	-0.31936178	-1.13617614	1.13617614
STX7	SIRPA	0.47444534	-0.66164446	-1.1360898	1.1360898
KANK1	C1orf131	-0.5872476	0.54881376	1.13606136	1.13606136
ZBTB10	PNPT1	-0.48682344	0.6491506	1.13597404	1.13597404
SET	RRN3	-0.47598445	0.6598715	1.13585595	1.13585595
SMYD3	FASTKD1	-0.47754773	0.6578913	1.13543903	1.13543903
MAPK10	DDX28	-0.41599435	0.71929973	1.13529408	1.13529408
USP9X	STX6	-0.46326324	0.67200565	1.13526889	1.13526889
SFN	RPS2	0.6195587	-0.51567465	-1.13523335	1.13523335
DCUN1D5	UNC5C	-0.5443721	0.5907842	1.1351563	1.1351563
FTL	RSPO3	-0.6523495	0.4827626	1.1351121	1.1351121
XRCC5	ST13	-0.5314729	0.602935	1.1344079	1.1344079
CEACAM1	ARPP19	-0.24685712	0.88734627	1.13420339	1.13420339
PSMA4	EBF1	-0.7309841	0.40315396	1.13413806	1.13413806
C10orf11	MRPL47	-0.46728003	0.6667803	1.13406033	1.13406033
ZWINT	KATNAL1	-0.69113505	0.4427822	1.13391725	1.13391725
RUFY3	RMI1	-0.80906034	0.3246371	1.13369744	1.13369744
ANAPC5	KIRREL	-0.26167244	0.87176037	1.13343281	1.13343281
ZBTB10	MSRB1	-0.69867396	0.43466866	1.13334262	1.13334262
MFAP3	SMOX	0.29790977	-0.83538514	-1.13329491	1.13329491
NAB2	OXR1	0.6971282	-0.4361048	-1.133233	1.133233
COMTD1	METTL3	-0.7782576	0.3549531	1.1332107	1.1332107
CYBRD1	PBXIP1	0.61696756	-0.51593876	-1.13290632	1.13290632

SSB	ATF6B	0.6079152	-0.52494735	-1.13286255	1.13286255
DDX6	MOCOS	-0.75473726	0.37804753	1.13278479	1.13278479
NOVA1	SLAMF1	-0.7421208	0.3905548	1.1326756	1.1326756
FOXN3	BID	-0.7600835	0.3725654	1.1326489	1.1326489
APC	TRMT1	-0.51154655	0.6210714	1.13261795	1.13261795
SLC26A7	ZNF467	0.5796991	-0.5528763	-1.1325754	1.1325754
GSTM3	SPAG16	0.7052215	-0.42716068	-1.13238218	1.13238218
VCAM1	SEPT11	0.52393156	-0.6083982	-1.13232976	1.13232976
LZTS1	ADPGK	-0.5322816	0.60000056	1.13228216	1.13228216
SF3B3	SOX4	-0.5731519	0.55906194	1.13221384	1.13221384
HINFP	ZNF704	0.55269563	-0.57949	-1.13218563	1.13218563
XRCC5	SPEF2	0.33801693	-0.7940226	-1.13203953	1.13203953
KLF12	C3orf17	0.24444143	-0.88751316	-1.13195459	1.13195459
USP4	PIK3IP1	-0.6369051	0.49503773	1.13194283	1.13194283
KDR	MUTYH	-0.51235974	0.6193598	1.13171954	1.13171954
UNC5C	EIF4E3	-0.46565974	0.6659232	1.13158294	1.13158294
SRSF1	TMEM163	-0.65805143	0.4734692	1.13152063	1.13152063
CCT6A	INIP	0.6225192	-0.5089921	-1.1315113	1.1315113
NAP1L1	PEX5	-0.49201137	0.63946354	1.13147491	1.13147491
S100A8	TM9SF3	-0.8613403	0.2699708	1.1313111	1.1313111
PCNA	TK1	0.8228824	-0.30838484	-1.13126724	1.13126724
FGFR1OP	GRM5	0.33630943	-0.7948636	-1.13117303	1.13117303
PDZRN3	PRICKLE1	0.6236975	-0.50745404	-1.13115154	1.13115154
NDN	PIR	-0.37358317	0.7574358	1.13101897	1.13101897
NOD1	PHF8	-0.3360022	0.7949799	1.1309821	1.1309821
ENSA	LMBRD2	-0.63105834	0.49982527	1.13088361	1.13088361
WNK1	CADM1	-0.49021128	0.64046866	1.13067994	1.13067994
SRPRB	COL6A3	-0.5655853	0.56490093	1.13048623	1.13048623
ATP5G1	SLC25A22	0.74318594	-0.38724834	-1.13043428	1.13043428
PRKRIR	FZD10	0.5740327	-0.5562316	-1.1302643	1.1302643
CSRNP1	ADAMTSL3	-0.53188175	0.5983693	1.13025105	1.13025105
CFLAR	LCMT2	-0.38975763	0.74031174	1.13006937	1.13006937
TPST2	TUFM	-0.35598665	0.7740523	1.13003895	1.13003895
USP9X	TOP3A	-0.74056876	0.38931173	1.12988049	1.12988049
SPTBN1	SLC25A29	0.2643557	-0.8654018	-1.1297575	1.1297575
RNF141	MRPS22	-0.3952176	0.73452985	1.12974745	1.12974745
GNAQ	DPAGT1	-0.42268896	0.70694435	1.12963331	1.12963331
TGM2	KCNJ14	0.44685513	-0.68271226	-1.12956739	1.12956739
URI1	EIF2B2	0.47938132	-0.65010625	-1.12948757	1.12948757
ARHGEF12	ZHX2	-0.8713973	0.25804472	1.12944202	1.12944202
TRIT1	MED15	-0.7668978	0.36247414	1.12937194	1.12937194
TMOD3	LAMA1	0.7644256	-0.36480615	-1.12923175	1.12923175
SH3RF1	PRICKLE1	0.7206998	-0.4083675	-1.1290673	1.1290673
TAOK3	C11orf31	-0.5723055	0.55674285	1.12904835	1.12904835
ESRRG	PTTG1	0.58200264	-0.5458637	-1.12786634	1.12786634
UNC5C	EYA2	-0.39259565	0.7351749	1.12777055	1.12777055
ZDHHC14	SPTBN1	0.36601388	-0.7617223	-1.12773618	1.12773618
USP4	PHF8	-0.46274564	0.6643198	1.12706544	1.12706544
COMMD3	ALS2	-0.65942615	0.46762642	1.12705257	1.12705257
KLF12	PLIN2	0.46273768	-0.66431105	-1.12704873	1.12704873
PPP1CA	ARNTL	-0.4130256	0.7139383	1.1269639	1.1269639
ADAMTS2	CEP112	0.6400124	-0.48662952	-1.12664192	1.12664192
XRCC5	MYOM1	-0.8924479	0.23383601	1.12628391	1.12628391
SMC4	FOXM1	0.8803084	-0.24588135	-1.12618975	1.12618975
XRCC5	RAB27A	0.66764253	-0.45821756	-1.12586009	1.12586009

MVP	EGR1	0.42920968	-0.6966332	-1.12584288	1.12584288
TRIO	SERPINE2	-0.6653005	0.460458	1.1257585	1.1257585
SAP30L	PTDSS1	-0.6952489	0.430162	1.1254109	1.1254109
USB1	P4HTM	-0.55986303	0.56542504	1.12528807	1.12528807
TUFM	CFLAR	-0.3440239	0.7812446	1.1252685	1.1252685
MAP4K4	KIF5B	0.9213858	-0.20386091	-1.12524671	1.12524671
TEFM	PHF8	-0.2520188	0.8730968	1.1251156	1.1251156
CDK6	NEGR1	-0.53276855	0.5922269	1.12499545	1.12499545
KIF11	TDRKH	0.566977	-0.55791175	-1.12488875	1.12488875
MOCS2	COL6A3	0.67372596	-0.45105097	-1.12477693	1.12477693
UBC	DPAGT1	-0.44108742	0.6836111	1.12469852	1.12469852
MCM6	ADGRA2	-0.6794518	0.44438645	1.12383825	1.12383825
PPP3CB	CNOT11	-0.47362438	0.65018284	1.12380722	1.12380722
PLOD3	P4HTM	-0.4091011	0.7145684	1.1236695	1.1236695
SRSF1	NID2	-0.4202769	0.7032825	1.1235594	1.1235594
GATAD1	MAD2L1BP	0.37162483	-0.7516285	-1.12325333	1.12325333
NUB1	PER3	-0.568129	0.55496514	1.12309414	1.12309414
GSTP1	RMI1	-0.46952736	0.65353674	1.1230641	1.1230641
XPO1	ZWILCH	-0.3493971	0.7736436	1.1230407	1.1230407
EZH2	TOP2A	0.9200974	-0.20291199	-1.12300939	1.12300939
ZNF106	MYL12A	-0.667963	0.45492303	1.12288603	1.12288603
POSTN	PPIL2	-0.4572314	0.6655928	1.1228242	1.1228242
SGCB	STX6	-0.61368513	0.5091123	1.12279743	1.12279743
USP4	NXF1	-0.6068722	0.5157963	1.1226685	1.1226685
GTF2I	NIPA2	-0.5681642	0.5544942	1.1226584	1.1226584
NFIB	CDCA7	-0.38508135	0.7375575	1.12263885	1.12263885
RAB4A	MALT1	-0.6640736	0.45848304	1.12255664	1.12255664
PCNA	WBP4	-0.4142487	0.70819056	1.12243926	1.12243926
IPO11	PDE7A	0.53529584	-0.58708745	-1.12238329	1.12238329
GNPTAB	TIRAP	-0.48869073	0.63363916	1.12232989	1.12232989
TNC	LOX	0.66996866	-0.45230088	-1.12226954	1.12226954
TRO	DPP6	0.6505287	-0.4717273	-1.122256	1.122256
CASP6	CEACAM1	-0.6031079	0.5189782	1.1220861	1.1220861
GEM	DDAH2	0.84802586	-0.27388868	-1.12191454	1.12191454
TRIM25	DCAF8	-0.2875623	0.83428013	1.12184243	1.12184243
SRPRB	MRPS14	-0.31723398	0.804539	1.12177298	1.12177298
MAP4K4	MYL12A	0.23769654	-0.88372785	-1.12142439	1.12142439
LAMA1	COL6A3	-0.5657195	0.5556284	1.1213479	1.1213479
SEC62	SOX4	0.4286253	-0.69246584	-1.12109114	1.12109114
CTNNA1	PALMD	0.33246347	-0.7886147	-1.12107817	1.12107817
APC	ZWINT	-0.28098065	0.8400697	1.12105035	1.12105035
KIF5B	MUTYH	0.38235688	-0.738514	-1.12087088	1.12087088
RELB	C1QBP	0.4056167	-0.71522164	-1.12083834	1.12083834
KATNAL1	CNOT11	-0.43312544	0.68765	1.12077544	1.12077544
IL2	SERPINH1	0.5195437	-0.60098195	-1.12052565	1.12052565
BGN	CYR61	0.66449624	-0.4559034	-1.12039964	1.12039964
MITF	GPR155	-0.5610565	0.5593304	1.1203869	1.1203869
NFE2L1	MICALL1	-0.36308724	0.7570519	1.12013914	1.12013914
WWC1	LMOD3	-0.6587433	0.46138453	1.12012783	1.12012783
LARP6	TRO	0.40539902	-0.7143764	-1.11977542	1.11977542
SLC26A7	CBLN2	0.58593476	-0.5337107	-1.11964546	1.11964546
SAP30L	MTHFD1L	-0.53122556	0.58840007	1.11962563	1.11962563
LAMA1	COL5A1	-0.40478745	0.7146711	1.11945855	1.11945855
SNRPB2	C8orf34	-0.74791545	0.37143376	1.11934921	1.11934921
PCBP2	DDX56	0.57266414	-0.54666054	-1.11932468	1.11932468

OPRK1	NEDD4L	-0.6309453	0.48837182	1.11931712	1.11931712
TIAL1	ZBTB44	0.49006957	-0.6291051	-1.11917467	1.11917467
AKAP10	NOL10	-0.45154223	0.6675367	1.11907893	1.11907893
RALBP1	CLASP2	0.42435616	-0.69464976	-1.11900592	1.11900592
MFAP3	RTN1	-0.37426382	0.7446095	1.11887332	1.11887332
TOP2A	DNMT1	0.6709932	-0.44785297	-1.11884617	1.11884617
KARS	ALAD	-0.52936184	0.58945954	1.11882138	1.11882138
NFIA	FARSA	-0.6645243	0.45410666	1.11863096	1.11863096
ABCG1	TEX10	-0.74455565	0.37405452	1.11861017	1.11861017
NUDCD2	FAM104B	0.66358286	-0.45487377	-1.11845663	1.11845663
XRCC5	CD48	0.71854156	-0.39971268	-1.11825424	1.11825424
ABCG1	NAT10	-0.56166935	0.556433	1.11810235	1.11810235
GTF2I	ATP5G1	-0.6610548	0.45701832	1.11807312	1.11807312
MAPK10	PDHB	-0.36461788	0.7530745	1.11769238	1.11769238
CWF19L2	XXYLT1	-0.75522155	0.3624661	1.11768765	1.11768765
TEAD4	FOS	0.6803981	-0.43715927	-1.11755737	1.11755737
CCT6A	PPA1	0.82732904	-0.2900238	-1.11735284	1.11735284
BMP15	TRMT1	0.28049842	-0.8368442	-1.11734262	1.11734262
FOXN3	BMP1	0.38640952	-0.730733	-1.11714252	1.11714252
SIRT7	PELP1	0.31591538	-0.8010819	-1.11699728	1.11699728
RBCK1	KCNRG	0.65722334	-0.45961252	-1.11683586	1.11683586
AK2	ZHX3	-0.3959509	0.72081524	1.11676614	1.11676614
NTRK2	SSB	-0.6127758	0.5039728	1.1167486	1.1167486
KATNAL1	NELFCD	-0.5515645	0.56514525	1.11670975	1.11670975
ABI2	HTATSF1	0.43066204	-0.68601036	-1.1166724	1.1166724
POPDC3	OPTN	-0.61364657	0.5026849	1.11633147	1.11633147
SNRPB2	RGS22	-0.5559868	0.5601941	1.1161809	1.1161809
SERBP1	TOP2A	0.8048916	-0.31118912	-1.11608072	1.11608072
KIAA0101	COL1A1	-0.52437705	0.5913987	1.11577575	1.11577575
ZSCAN20	VSIG1	0.54380625	-0.57167643	-1.11548268	1.11548268
YME1L1	AK9	0.47880617	-0.63666534	-1.11547151	1.11547151
CXCR4	COL15A1	-0.59120715	0.5242562	1.11546335	1.11546335
SPCS3	ANKRD28	-0.354542	0.76068467	1.11522667	1.11522667
LIG3	SNX29	-0.6329619	0.4821287	1.1150906	1.1150906
SH3D19	LMBRD2	-0.53932196	0.57565933	1.11498129	1.11498129
RGS5	MUTYH	-0.504653	0.6102097	1.1148627	1.1148627
RUFY3	NDFIP2	-0.6085199	0.5061176	1.1146375	1.1146375
NEGR1	NDFIP2	0.85021955	-0.26427695	-1.1144965	1.1144965
MDM1	LMOD3	0.67617935	-0.4382559	-1.11443525	1.11443525
APC	XPO5	-0.5323083	0.5820152	1.1143235	1.1143235
TBL1XR1	MLST8	0.44174793	-0.67253107	-1.114279	1.114279
C4A	MFSD12	-0.63765174	0.47636136	1.1140131	1.1140131
ANKRD35	TANC2	0.44981924	-0.66402555	-1.11384479	1.11384479
RNF130	NT5DC1	-0.44506335	0.66873145	1.1137948	1.1137948
VPS16	CEP112	0.51152223	-0.60205567	-1.1135779	1.1135779
ESRRG	PVR	0.7541373	-0.3593888	-1.1135261	1.1135261
PRR14L	PHF8	-0.5912207	0.5222675	1.1134882	1.1134882
KANK1	PRICKLE1	0.52613926	-0.5873488	-1.11348806	1.11348806
SOX4	FAM129B	-0.7335648	0.3798223	1.1133871	1.1133871
UNC5C	ADGRV1	-0.6359052	0.47721922	1.11312442	1.11312442
COL9A1	ITGA8	-0.46693596	0.64613444	1.1130704	1.1130704
FOXN3	PSMA4	-0.6483671	0.4646596	1.1130267	1.1130267
C12orf4	STAMBPL1	-0.2499736	0.86295563	1.11292923	1.11292923
UNC5C	C11orf63	-0.51551235	0.5972312	1.11274355	1.11274355
EPS15	SOX4	0.58418655	-0.52847075	-1.1126573	1.1126573

IDS	BID	-0.6878863	0.4246857	1.112572	1.112572
MSR1	ENOSF1	-0.6495065	0.46299192	1.11249842	1.11249842
FMO2	CTNNA1	0.37373853	-0.7387309	-1.11246943	1.11246943
CASP6	MALT1	-0.6552626	0.4567788	1.1120414	1.1120414
PDGFC	LMBRD2	-0.49771747	0.61420333	1.1119208	1.1119208
LAMA1	CNTNAP1	-0.3957782	0.7160822	1.1118604	1.1118604
ARHGEF12	SSPN	-0.91196537	0.19987498	1.11184035	1.11184035
RPL27A	RPN2	0.5089794	-0.60281783	-1.11179723	1.11179723
IP6K2	NEGR1	0.5524746	-0.559207	-1.1116816	1.1116816
ZBTB20	C1orf131	-0.69002897	0.42159963	1.1116286	1.1116286
COL1A1	ECM2	0.5056936	-0.60564077	-1.11133437	1.11133437
NEGR1	PNPT1	0.58121806	-0.5300154	-1.11123346	1.11123346
DPAGT1	TM9SF3	-0.5024865	0.60847557	1.11096207	1.11096207
RANGAP1	PNPT1	0.58743787	-0.52351886	-1.11095673	1.11095673
GMPS	GSS	0.5788781	-0.53188807	-1.11076617	1.11076617
TRPM3	TPST2	-0.82485974	0.2858924	1.11075214	1.11075214
MAPKAP1	GET4	-0.51234984	0.59840006	1.1107499	1.1107499
SP3	HDAC1	-0.43330374	0.6773109	1.11061464	1.11061464
SCARB2	GIPC1	0.50431967	-0.6062013	-1.11052097	1.11052097
GTF3C2	MTHFSD	0.2629797	-0.84749097	-1.11047067	1.11047067
ESRRG	PID1	-0.69109595	0.41924796	1.11034391	1.11034391
PPP6C	LOC10106017[illegible]	0.77733207	-0.3328423	-1.11017437	1.11017437
KCND2	KIAA0391	-0.6014136	0.50842655	1.10984015	1.10984015
HLF	YME1L1	0.6068485	-0.50285363	-1.10970213	1.10970213
IGF2R	LAGE3	-0.62079984	0.48883292	1.10963276	1.10963276
TRIT1	SMYD3	-0.3451991	0.7642617	1.1094608	1.1094608
SNTB2	PGS1	0.5435248	-0.5658277	-1.1093525	1.1093525
POPDC3	FAM149A	-0.5784731	0.53082037	1.10929347	1.10929347
NIPAL2	GPAT4	-0.48873532	0.6205215	1.10925682	1.10925682
GTF2H1	ADCY1	-0.71176094	0.39716643	1.10892737	1.10892737
NFE2L1	ARPC5L	-0.745861	0.36297947	1.10884047	1.10884047
UBC	SVBP	0.5896746	-0.5191626	-1.1088372	1.1088372
VPS33B	MLST8	-0.4795739	0.62919044	1.10876434	1.10876434
YARS	CST2	0.4269355	-0.68166924	-1.10860474	1.10860474
NOL8	ESRP2	0.58806103	-0.5204917	-1.10855273	1.10855273
PSMB3	RNASE4	-0.54029167	0.56824654	1.10853821	1.10853821
SSB	LEPR	-0.41202232	0.69633913	1.10836145	1.10836145
AIG1	SPIRE2	-0.6336465	0.47457623	1.10822273	1.10822273
SEH1L	WDR13	-0.61325246	0.49483678	1.10808924	1.10808924
BRAP	FAM43A	-0.76897764	0.3391023	1.10807994	1.10807994
MRPS22	NET1	-0.32656702	0.7813626	1.10792962	1.10792962
PTGER3	PBXIP1	0.7035441	-0.40432832	-1.10787242	1.10787242
SP3	ZNF704	0.42617965	-0.68144774	-1.10762739	1.10762739
HDAC11	CCT3	-0.70223165	0.4053549	1.10758655	1.10758655
TDRKH	KIAA0232	-0.8015102	0.30604595	1.10755615	1.10755615
CSNK2A1	THRB	-0.34297064	0.7645322	1.10750284	1.10750284
POLR2D	NEGR1	0.6551444	-0.4520929	-1.1072373	1.1072373
EIF4G3	PTPN21	0.5074395	-0.5996893	-1.1071288	1.1071288
MANF	IL32	0.6997627	-0.40732235	-1.10708505	1.10708505
CBS	SERPINE2	-0.5597397	0.5472537	1.1069934	1.1069934
ESRRG	NPR3	-0.2580772	0.8488113	1.1068885	1.1068885
XRCC5	PPM1A	-0.5800703	0.52670604	1.10677634	1.10677634
RUFY3	TUSC3	-0.43516392	0.67157584	1.10673976	1.10673976
CTNNA1	LRRC16A	-0.58339894	0.5230261	1.10642504	1.10642504
MCM3	CD48	0.6075954	-0.49880737	-1.10640277	1.10640277

SET	KIRREL	-0.7055112	0.40080148	1.10631268	1.10631268
CTNNA1	EFHD1	0.49597326	-0.61012715	-1.10610041	1.10610041
HDAC11	FAM216A	-0.75503457	0.35096955	1.10600412	1.10600412
UTP18	DCAF7	-0.5379704	0.5679213	1.1058917	1.1058917
TUFM	CRY2	-0.6256237	0.48006207	1.10568577	1.10568577
MVP	JUN	0.5060352	-0.5994051	-1.1054403	1.1054403
POLN	RMDN1	-0.57388276	0.53124464	1.1051274	1.1051274
CCT3	SYCP2	-0.7417758	0.36332184	1.10509764	1.10509764
HDAC11	ADSL	-0.5354228	0.5695562	1.104979	1.104979
CLCN4	HIST1H1D	0.55637884	-0.54855186	-1.1049307	1.1049307
DIEXF	TK1	0.5095507	-0.5953017	-1.1048524	1.1048524
DCUN1D5	NDN	-0.4472023	0.65764505	1.10484735	1.10484735
LPGAT1	PRUNE2	0.6342781	-0.47054955	-1.10482765	1.10482765
EIF4E	QSOX1	0.46222296	-0.6419051	-1.10412806	1.10412806
SH3PXD2A	USP4	0.56821656	-0.53590643	-1.10412299	1.10412299
RBPJ	FZD8	0.614526	-0.48959163	-1.10411763	1.10411763
RPL14	MTURN	-0.6323293	0.47165772	1.10398702	1.10398702
ESRRG	WNK1	-0.32525167	0.7786542	1.10390587	1.10390587
CCT3	ABCA1	-0.43195173	0.67189777	1.1038495	1.1038495
CSF3R	QSOX1	-0.6434909	0.46011513	1.10360603	1.10360603
DHRS12	SOX4	0.61314327	-0.49029246	-1.10343573	1.10343573
CPXM1	DDR2	0.5634718	-0.5397569	-1.1032287	1.1032287
GPC1	EGR1	0.60559565	-0.49761304	-1.10320869	1.10320869
SEL1L3	EGR1	0.4690538	-0.63406825	-1.10312205	1.10312205
MFAP3	PDE7A	0.43145177	-0.67163664	-1.10308841	1.10308841
MCM6	UBTF	-0.43380877	0.66925967	1.10306844	1.10306844
COG6	ANTXR2	-0.4602705	0.6427911	1.1030616	1.1030616
VEGFA	POLN	0.37214333	-0.73089737	-1.1030407	1.1030407
SRPRB	NID2	-0.4921347	0.61089694	1.10303164	1.10303164
WWOX	CADM1	-0.532145	0.57086766	1.10301266	1.10301266
COL4A1	SSPN	-0.22379355	0.8791302	1.10292375	1.10292375
INF2	CSNK2A1	0.25405782	-0.84873706	-1.10279488	1.10279488
HDAC11	MCM3	-0.80089515	0.30178115	1.1026763	1.1026763
MCM6	SPEF2	0.33603507	-0.7665464	-1.10258147	1.10258147
PDE4DIP	LAP3	-0.54272485	0.55948573	1.10221058	1.10221058
ESRRG	IL4	0.7409403	-0.3611838	-1.1021241	1.1021241
CHST11	COL5A1	-0.30949667	0.7924941	1.10199077	1.10199077
RBM17	GSTM5	0.8908159	-0.2110684	-1.1018843	1.1018843
PDHB	FAM76A	-0.45346656	0.64823055	1.10169711	1.10169711
ZNF385D	RGS5	-0.52375436	0.5779084	1.10166276	1.10166276
NSMF	MYO19	-0.27359405	0.8279819	1.10157595	1.10157595
TOR1AIP1	GSTM5	0.47559306	-0.62589824	-1.1014913	1.1014913
LOC441204	IKZF1	0.6373879	-0.46403444	-1.10142234	1.10142234
NUDCD2	CLRN1	0.6322629	-0.46882135	-1.10108425	1.10108425
HLF	LMBRD2	-0.75336754	0.3476957	1.10106324	1.10106324
XPO1	CDC7	-0.5545394	0.5464426	1.100982	1.100982
HSPB2	TSPAN9	-0.84016883	0.26078135	1.10095018	1.10095018
UNC5C	FIGF	-0.44786382	0.65277255	1.10063637	1.10063637
SOD2	GABARAPL1	-0.59948707	0.50114375	1.10063082	1.10063082
DUSP3	LOC10050754[illegible]	-0.571831	0.5287802	1.1006112	1.1006112
FASTKD1	ZNF384	-0.35077387	0.74961734	1.10039121	1.10039121
CCNI	PPP3R1	0.62733185	-0.47304848	-1.10038033	1.10038033
MYOC	INTS6	0.5125604	-0.5876353	-1.1001957	1.1001957
ITSN1	LTF	-0.6379661	0.46220395	1.10017005	1.10017005
USP4	USP9X	-0.60097766	0.49917448	1.10015214	1.10015214

PARK2	KCND3	-0.6510445	0.44853646	1.09958096	1.09958096
LOC10099669:	C1orf131	0.29363325	-0.80593115	-1.0995644	1.0995644
MFAP3	RCHY1	0.6326425	-0.46688306	-1.09952556	1.09952556
STX7	STK10	0.51113576	-0.58835524	-1.099491	1.099491
ZBTB44	GPBP1L1	0.4460873	-0.6533478	-1.0994351	1.0994351
SLC26A7	CASP5	-0.7475734	0.3512127	1.0987861	1.0987861
CDK6	COL6A3	0.54913414	-0.549608	-1.09874214	1.09874214
PDZD2	CNOT11	-0.4704207	0.6282298	1.0986505	1.0986505
KANK1	QSOX1	0.4621131	-0.6364802	-1.0985933	1.0985933
DCN	KIF13A	0.84489316	-0.25363216	-1.09852532	1.09852532
KALRN	GEMIN8	-0.60244614	0.49565548	1.09810162	1.09810162
RACGAP1	SNX18	-0.39255902	0.70535016	1.09790918	1.09790918
CRY2	MTHFD1	0.6899053	-0.40795574	-1.09786104	1.09786104
ADAMTS2	DIEXF	-0.470853	0.6267737	1.0976267	1.0976267
EZH2	PRR11	0.88562864	-0.21189785	-1.09752649	1.09752649
CTNNA1	ELF4	-0.32121375	0.7762791	1.09749285	1.09749285
XRCC5	LDLRAD4	-0.4865959	0.6105527	1.0971486	1.0971486
CSF3R	FGF18	-0.63752395	0.45956895	1.0970929	1.0970929
PTPRG	NTSR2	0.42506647	-0.67199826	-1.09706473	1.09706473
MED16	WDR54	-0.52408904	0.57293016	1.0970192	1.0970192
PARPBP	MYO19	0.83737975	-0.2594363	-1.09681605	1.09681605
ATP1A2	PRUNE2	0.521971	-0.57466865	-1.09663965	1.09663965
RNF130	BST2	-0.80648035	0.29008788	1.09656823	1.09656823
GEM	LTBP4	0.7546099	-0.34157214	-1.09618204	1.09618204
RBPJ	BST2	-0.532398	0.56374615	1.09614415	1.09614415
COL27A1	POLN	0.38460052	-0.71151024	-1.09611076	1.09611076
SRD5A1	CFLAR	-0.55929357	0.5366221	1.09591567	1.09591567
SOX4	RCC2	-0.6333788	0.462505	1.0958838	1.0958838
RBM17	DAZAP1	-0.5438388	0.55200905	1.09584785	1.09584785
DKC1	TK1	0.5959912	-0.49982163	-1.09581283	1.09581283
EIF4E3	ZC3HAV1L	-0.74305654	0.3526368	1.09569334	1.09569334
SNAP29	DBI	-0.29147232	0.8037116	1.09518392	1.09518392
WIPI2	DNAJB1	0.72459245	-0.37055448	-1.09514693	1.09514693
SSBP2	PSMA4	-0.52353054	0.57157785	1.09510839	1.09510839
UBC	ASB6	-0.5738544	0.5210189	1.0948733	1.0948733
STAR	UST	-0.7026901	0.39211902	1.09480912	1.09480912
STAT3	JUN	0.5783673	-0.5161511	-1.0945184	1.0945184
CSTF3	POLN	0.39007404	-0.70415616	-1.0942302	1.0942302
RAE1	WDR13	-0.77661127	0.3175505	1.09416177	1.09416177
CTSL	TMEM2	-0.28691518	0.807211	1.09412618	1.09412618
ADRB1	FOS	-0.7004459	0.39361575	1.09406165	1.09406165
ARNTL	ANTXR2	0.6432149	-0.45045993	-1.09367483	1.09367483
PARVA	THBS3	0.6132847	-0.48036277	-1.09364747	1.09364747
TULP4	SYCP1	-0.28450742	0.8090552	1.09356262	1.09356262
SSB	LARP6	-0.30586734	0.78756064	1.09342798	1.09342798
MYD88	SLC25A29	-0.43107006	0.66233677	1.09340683	1.09340683
MFAP3	SPG20	-0.35003844	0.7431982	1.09323664	1.09323664
CA12	CTNNA1	-0.4766376	0.61650926	1.09314686	1.09314686
TAF1C	PHF8	-0.25890416	0.8342368	1.09314096	1.09314096
LARP6	CERS5	0.7644308	-0.3285904	-1.0930212	1.0930212
TCF3	C1QBP	0.6729451	-0.41988254	-1.09282764	1.09282764
COMMD3	VAPB	-0.39712018	0.6956961	1.09281628	1.09281628
RGS5	DDR2	0.83626884	-0.25643924	-1.09270808	1.09270808
CA12	WWTR1	0.48345277	-0.6091954	-1.09264817	1.09264817
PRICKLE1	EIF4E3	0.48361364	-0.6089965	-1.09261014	1.09261014

MAP4K4	ADAMTS1	0.5847378	-0.50772613	-1.09246393	1.09246393
RACGAP1	ARHGEF37	-0.62111074	0.47134575	1.09245649	1.09245649
RSU1	ISYNA1	0.3043943	-0.78771	-1.0921043	1.0921043
PTPN11	UNC5C	-0.6008373	0.4910811	1.0919184	1.0919184
SOX4	ISCU	0.58619326	-0.5056614	-1.09185466	1.09185466
MFAP3	ZBTB10	-0.42119586	0.67061174	1.0918076	1.0918076
MANF	LPCAT2	0.47345734	-0.61791825	-1.09137559	1.09137559
CPED1	SIRPA	0.7060412	-0.38530594	-1.09134714	1.09134714
CBLB	NIPA2	-0.4985286	0.59281737	1.09134597	1.09134597
TOP2A	FDPS	0.36808747	-0.72303575	-1.09112322	1.09112322
APC	COMMD3	-0.47250283	0.61860675	1.09110958	1.09110958
TRIM27	AP3S2	-0.47618186	0.6149186	1.09110046	1.09110046
NUP62	BFSP1	-0.5619732	0.5289272	1.0909004	1.0909004
EIF4G3	PHF8	0.47926238	-0.61163586	-1.09089824	1.09089824
CYR61	FCGBP	0.5908737	-0.49994856	-1.09082226	1.09082226
ITSN1	STX6	-0.7359009	0.35485414	1.09075504	1.09075504
CDC25B	MDN1	-0.39749587	0.6932275	1.09072337	1.09072337
SGCB	QSOX1	0.5305695	-0.5601274	-1.0906969	1.0906969
IFNAR2	HELLS	-0.4181368	0.67231685	1.09045365	1.09045365
ZBTB7A	ZBTB24	0.51536334	-0.5750412	-1.09040454	1.09040454
TUBB	PFDN4	0.48534355	-0.605056	-1.09039955	1.09039955
LDLRAD4	PRICKLE1	0.6908707	-0.39948794	-1.09035864	1.09035864
USP9X	AGPAT5	-0.29262182	0.7975497	1.09017152	1.09017152
GALNT2	PPM1L	-0.44999444	0.6400124	1.09000684	1.09000684
SLC25A22	SLC25A5	0.7261504	-0.36366674	-1.08981714	1.08981714
PPM1A	PSMA4	-0.660219	0.42957717	1.08979617	1.08979617
SPEF2	NIPA2	0.26489782	-0.8248661	-1.08976392	1.08976392
UBE2B	ATP6V1G1	-0.48814714	0.60152346	1.0896706	1.0896706
STAT3	SOCS3	0.6257423	-0.46392202	-1.08966432	1.08966432
URB2	CCDC117	0.59291196	-0.496619	-1.08953096	1.08953096
TCEB3	LCA5	-0.33578095	0.7536885	1.08946945	1.08946945
TBL1XR1	DCAF11	0.729263	-0.3602027	-1.0894657	1.0894657
LMO3	SAA4	-0.56854934	0.5207096	1.08925894	1.08925894
APC	HNRNPC	-0.5265743	0.5624219	1.0889962	1.0889962
MFAP3	LMO3	-0.71190006	0.3769768	1.08887686	1.08887686
AHNAK	LMBRD2	-0.5645177	0.524338	1.0888557	1.0888557
TAOK3	ITGA8	0.7220075	-0.36683294	-1.08884044	1.08884044
SRSF1	WAC	-0.41110066	0.6777352	1.08883586	1.08883586
NEDD4L	SSB	-0.56789535	0.520846	1.08874135	1.08874135
RELA	ABCA1	-0.45602003	0.6326623	1.08868233	1.08868233
SLC7A1	CCDC117	0.5806442	-0.5078374	-1.0884816	1.0884816
TMOD3	RACGAP1	0.51852137	-0.5699433	-1.08846467	1.08846467
FOSB	TMEM163	0.68087983	-0.40756282	-1.08844265	1.08844265
GABBR1	PHF8	0.7700236	-0.318404	-1.0884276	1.0884276
SKP1	MSRB1	-0.67627966	0.41179195	1.08807161	1.08807161
CYBRD1	PSMB3	-0.6525976	0.43540448	1.08800208	1.08800208
UNC5C	CYP2B6	-0.48121583	0.60674864	1.08796447	1.08796447
COL3A1	H2AFY	-0.3795379	0.70836216	1.08790006	1.08790006
SCRN1	TPM4	0.45293704	-0.6347734	-1.08771044	1.08771044
LIG3	SOX4	-0.67627287	0.4114105	1.08768337	1.08768337
KPNA4	TCEA3	-0.53039885	0.5572238	1.08762265	1.08762265
QSOX1	JUND	0.5193662	-0.56821823	-1.08758443	1.08758443
RBPJ	CCM2	-0.5958817	0.49147847	1.08736017	1.08736017
THRB	PSMB3	-0.7436891	0.34356788	1.08725698	1.08725698
KAT6B	MAP4	0.56209	-0.5251118	-1.0872018	1.0872018

CSRP2BP	DNAJC9	-0.50650805	0.5806158	1.08712385	1.08712385
TOP2A	MCM5	0.7187027	-0.36840472	-1.08710742	1.08710742
NDN	MAGED4	0.6874514	-0.3993683	-1.0868197	1.0868197
TRIM2	BCAR1	-0.6255761	0.46121898	1.08679508	1.08679508
SSRP1	MCM4	0.79677445	-0.28996253	-1.08673698	1.08673698
SPAG16	SNRPC	-0.46467236	0.6219489	1.08662126	1.08662126
KIF5B	PRRC2A	0.42077848	-0.6658427	-1.08662118	1.08662118
PDE4DIP	MOCOS	-0.67457616	0.41170415	1.08628031	1.08628031
TM9SF3	NIPA2	-0.23149504	0.85472727	1.08622231	1.08622231
PDZRN3	MRPL47	-0.76207566	0.32395858	1.08603424	1.08603424
IPO11	CHD2	-0.49395874	0.5920686	1.08602734	1.08602734
XPO1	STK10	-0.45359528	0.63242817	1.08602345	1.08602345
APBB2	TOR1AIP1	0.7359421	-0.35006818	-1.08601028	1.08601028
HDAC1	CASP6	-0.6237511	0.46194217	1.08569327	1.08569327
GTF3C1	ARPIN	0.49155882	-0.5941319	-1.08569072	1.08569072
ZNF385D	NFIA	-0.62405753	0.46152747	1.085585	1.085585
RACGAP1	KANK1	-0.6832969	0.40213504	1.08543194	1.08543194
EPB41L4A	HLA-C	0.44018877	-0.6449088	-1.08509757	1.08509757
TUSC3	PRUNE2	-0.7968592	0.28818333	1.08504253	1.08504253
MMS19	FAM63B	0.5622396	-0.5227	-1.0849396	1.0849396
KLF4	SCML2	-0.41872182	0.6661863	1.08490812	1.08490812
DHRS12	TIMM23	-0.56525594	0.51961154	1.08486748	1.08486748
STX8	SERPINA3	-0.56547856	0.5193852	1.08486376	1.08486376
THRB	QSOX1	0.520741	-0.56391996	-1.08466096	1.08466096
AIMP1	PDE7A	0.43965617	-0.64500225	-1.08465842	1.08465842
CPNE3	QSOX1	0.48347405	-0.6010246	-1.08449865	1.08449865
SPPL2A	RIN3	0.3932637	-0.6911485	-1.0844122	1.0844122
MAD2L1BP	CBLB	-0.47125065	0.6129404	1.08419105	1.08419105
MCM6	ADAMTS2	-0.38815334	0.69603246	1.0841858	1.0841858
CASP8	FMO2	-0.5562472	0.52791	1.0841572	1.0841572
PLA2G2A	CEACAM1	0.42439938	-0.65970427	-1.08410365	1.08410365
INTS6	LZTS1	-0.6230984	0.4609288	1.0840272	1.0840272
PMM2	EPHX2	-0.5834163	0.50050664	1.08392294	1.08392294
ARPP21	ADAMTS1	-0.7609822	0.32292363	1.08390583	1.08390583
GSTM1	TXNDC17	-0.75178003	0.3318967	1.08367673	1.08367673
SERPINA1	TSPAN9	0.7320173	-0.3514551	-1.0834724	1.0834724
HIVEP3	CHST15	0.6056567	-0.47775555	-1.08341225	1.08341225
IGF1R	ELOVL5	-0.5569788	0.526405	1.0833838	1.0833838
ZBTB10	NOL9	-0.5248361	0.5585209	1.083357	1.083357
BCL2L1	DNMT1	0.39891997	-0.6843761	-1.08329607	1.08329607
TBC1D9B	UST	0.49365425	-0.58957094	-1.08322519	1.08322519
ITGB1	THBS3	0.47024858	-0.6129429	-1.08319148	1.08319148
GALNT2	KIAA1715	-0.35888886	0.7242846	1.08317346	1.08317346
TRIM2	SORL1	0.6542999	-0.4288558	-1.0831557	1.0831557
KATNAL1	NVL	-0.36987355	0.71326244	1.08313599	1.08313599
CSRP2BP	GET4	-0.6641821	0.4188429	1.083025	1.083025
SUFU	CDK5	-0.36680838	0.71616375	1.08297213	1.08297213
RUFY3	VANGL1	-0.38680127	0.6961557	1.08295697	1.08295697
AGPAT5	LOC10099669	0.36950636	-0.71333957	-1.08284593	1.08284593
PFDN4	PPP1R13L	0.55293137	-0.5298478	-1.08277917	1.08277917
HDAC11	DNAJC9	-0.7757294	0.30704254	1.08277194	1.08277194
ASB9	RAB40B	-0.60713726	0.47551665	1.08265391	1.08265391
SRD5A1	STK10	-0.28515568	0.7974636	1.08261928	1.08261928
CPXM1	EZH2	-0.5001971	0.5822443	1.0824414	1.0824414
ABI2	IFT81	0.582227	-0.50003743	-1.08226443	1.08226443

CAST	ESRRG	-0.71303475	0.36917806	1.08221281	1.08221281
BIK	BCL2	-0.7581294	0.32391524	1.08204464	1.08204464
TACC1	KIAA0391	-0.6769218	0.4049558	1.0818776	1.0818776
HSP90AA1	CDCA7	0.48599392	-0.59571517	-1.08170909	1.08170909
YARS	RNF150	-0.7509226	0.33077267	1.08169527	1.08169527
MKLN1	PDE7A	0.57875013	-0.5027882	-1.08153833	1.08153833
MED15	CREB3L4	-0.6944369	0.38705572	1.08149262	1.08149262
RSPO3	PLEKHG3	0.6452728	-0.43591392	-1.08118672	1.08118672
EPS8	NUDT21	-0.5303873	0.55078363	1.08117093	1.08117093
SGCB	PDHB	-0.4068733	0.6742495	1.0811228	1.0811228
EPM2A	ZNF804A	-0.65477246	0.42629537	1.08106783	1.08106783
CCNA2	RGS5	-0.7045062	0.37650302	1.08100922	1.08100922
VCAM1	AIDA	0.25666496	-0.8242073	-1.08087226	1.08087226
PCBP2	LNX1	0.30501166	-0.7758244	-1.08083606	1.08083606
XPNPEP1	DFNB31	-0.31586328	0.7643926	1.08025588	1.08025588
GNAQ	TEC	0.5640928	-0.51608664	-1.08017944	1.08017944
MPPED2	GSTM3	0.46799046	-0.61190784	-1.0798983	1.0798983
CADM1	RNF180	-0.34066024	0.7392269	1.07988714	1.07988714
PDLIM4	SLC25A29	-0.29000106	0.7897188	1.07971986	1.07971986
BUB1B	IMPDH1	0.40742093	-0.6722945	-1.07971543	1.07971543
HLF	SLC25A29	0.5068038	-0.5728954	-1.0796992	1.0796992
NFKBIE	MIB2	0.6234857	-0.4561796	-1.0796653	1.0796653
CEACAM1	CD164	-0.41444218	0.66521865	1.07966083	1.07966083
MCM6	HLA-B	-0.44576102	0.6338162	1.07957722	1.07957722
ARL1	ARHGEF12	-0.6428289	0.43668255	1.07951145	1.07951145
NUDCD2	SIRPA	-0.76419	0.31516847	1.07935847	1.07935847
PIK3R1	RPL14	-0.3694979	0.7096241	1.079122	1.079122
MCM3	TIAL1	-0.17475535	0.90415627	1.07891162	1.07891162
PPP1CB	TEC	0.4694317	-0.6090517	-1.0784834	1.0784834
TRIM27	CSRP2BP	-0.37207332	0.706193	1.07826632	1.07826632
WRAP53	CHD2	-0.73855925	0.33959028	1.07814953	1.07814953
ARPP21	ETV3	-0.78889364	0.28922898	1.07812262	1.07812262
CELF2	TBC1D9B	-0.6285421	0.44950414	1.07804624	1.07804624
C12orf49	EGR1	0.46467733	-0.6133107	-1.07798803	1.07798803
ESRRG	NFIA	-0.7137432	0.36423326	1.07797646	1.07797646
MED15	RDH11	-0.68108755	0.3968181	1.07790565	1.07790565
ESRRG	MPL	-0.34488168	0.732917	1.07779868	1.07779868
CPNE3	MRPS22	-0.27761218	0.8001605	1.07777268	1.07777268
KALRN	ACTN3	0.49487972	-0.58286613	-1.07774585	1.07774585
COL5A1	SDC2	0.59049535	-0.4870071	-1.07750245	1.07750245
DPT	HSD17B6	0.3889772	-0.688438	-1.0774152	1.0774152
TUSC3	CFLAR	-0.35882825	0.71855557	1.07738382	1.07738382
GSS	PARPBP	0.66556865	-0.41179305	-1.0773617	1.0773617
CPNE3	MMP15	0.6571584	-0.4200858	-1.0772442	1.0772442
SERBP1	ZBTB10	-0.6078574	0.4693091	1.0771665	1.0771665
MRPS22	RMDN1	-0.5238984	0.55324286	1.07714126	1.07714126
CSNK2A1	SPTBN1	-0.6092908	0.4678246	1.0771154	1.0771154
HTT	FAM111A	-0.56689245	0.51018673	1.07707918	1.07707918
EXOG	ZNF500	-0.5426186	0.5344071	1.0770257	1.0770257
MAP4K4	SOX4	-0.34879634	0.7281981	1.07699444	1.07699444
KCNRG	DNAJC9	0.57508415	-0.5019004	-1.07698455	1.07698455
MLST8	DMXL2	-0.5233585	0.5536208	1.0769793	1.0769793
SAP18	QSOX1	0.5107616	-0.5661441	-1.0769057	1.0769057
DYSF	C10orf90	0.6104235	-0.4664077	-1.0768312	1.0768312
YARS	VAMP4	-0.5453731	0.53137356	1.07674666	1.07674666

GNAQ	ZNF385D	-0.49420822	0.58243734	1.07664556	1.07664556
GTF3C1	BRF1	0.4512072	-0.62538743	-1.07659463	1.07659463
ZNF385D	HINFP	-0.55193686	0.5242294	1.07616626	1.07616626
MDN1	HDAC1	-0.5081038	0.5678982	1.076002	1.076002
ZFP36L1	PRR14L	0.7042122	-0.37167665	-1.07588885	1.07588885
FAM216A	ZNF678	-0.49879515	0.57697713	1.07577228	1.07577228
COL27A1	THBS3	-0.34538007	0.73021555	1.07559562	1.07559562
TCEB3	SNX1	-0.43235403	0.6431276	1.07548163	1.07548163
DHRS12	NIPA2	-0.61632407	0.45902973	1.0753538	1.0753538
ZWINT	FANCB	0.72250295	-0.35273087	-1.07523382	1.07523382
MRPL47	MAN2B2	-0.8227664	0.2523793	1.0751457	1.0751457
UBE2D1	CFLAR	-0.39442432	0.68068665	1.07511097	1.07511097
SSBP2	RAB13	-0.75746703	0.3176244	1.07509143	1.07509143
HSP90AA1	H2AFY	0.39460644	-0.68046486	-1.0750713	1.0750713
ACVR2A	TLL2	0.8313492	-0.24354647	-1.07489567	1.07489567
EIF4G3	PHF10	0.5219501	-0.5529355	-1.0748856	1.0748856
SSPN	MRPS22	-0.35967207	0.7151217	1.07479377	1.07479377
TRAPPC3	ZBTB7A	-0.5883453	0.48621237	1.07455767	1.07455767
SET	SAA4	0.6535683	-0.42092815	-1.07449645	1.07449645
PPIL2	NEGR1	0.18870448	-0.8856535	-1.07435798	1.07435798
RBMS3	NEGR1	-0.5718115	0.5023922	1.0742037	1.0742037
SPAG5	PRR11	0.89600533	-0.17797133	-1.07397666	1.07397666
SORBS1	PRICKLE1	0.39259452	-0.68136865	-1.07396317	1.07396317
KANK1	STX6	-0.5675339	0.50640213	1.07393603	1.07393603
ZWINT	DCAF7	-0.59859043	0.4750465	1.07363693	1.07363693
FLNB	ZNF500	-0.6453024	0.42824164	1.07354404	1.07354404
POSTN	HELLS	-0.23205052	0.84146	1.07351052	1.07351052
SNX5	GALNT2	-0.59128505	0.481777	1.07306205	1.07306205
GSS	DCAF8	-0.23436886	0.83849144	1.0728603	1.0728603
IFNAR2	DFNB31	-0.5066317	0.5661708	1.0728025	1.0728025
CHST11	MDN1	-0.7350342	0.3376991	1.0727333	1.0727333
PAPSS1	STX7	0.38646644	-0.68616843	-1.07263487	1.07263487
CALCOCO2	SOX4	0.48176843	-0.59080935	-1.07257778	1.07257778
ARHGEF37	DNAJC9	-0.67550135	0.39695925	1.0724606	1.0724606
MFAP3	ASB1	-0.33339313	0.7390529	1.07244603	1.07244603
ESRRG	TSKU	-0.50412405	0.56826156	1.07238561	1.07238561
UBC	TIMELESS	-0.38052148	0.6916902	1.07221168	1.07221168
AGPAT5	CCDC117	0.3875129	-0.6846923	-1.0722052	1.0722052
COL4A1	ACTN3	0.45797643	-0.6141607	-1.07213713	1.07213713
DNAJC15	C11orf98	-0.5191392	0.55289644	1.07203564	1.07203564
ADAMTS3	ISM1	0.5637015	-0.5081534	-1.0718549	1.0718549
CETP	GRWD1	-0.5960001	0.4758535	1.0718536	1.0718536
PDZRN3	AGPAT5	-0.6798141	0.3920203	1.0718344	1.0718344
PGP	EZH2	0.61186	-0.45984674	-1.07170674	1.07170674
NEGR1	AGTRAP	0.33158484	-0.7400453	-1.07163014	1.07163014
LDLRAD4	C1QTNF3	0.72749805	-0.34408936	-1.07158741	1.07158741
ALAD	PRICKLE1	0.50911564	-0.56226206	-1.0713777	1.0713777
DDX6	TUFM	-0.2598255	0.81133866	1.07116416	1.07116416
CTNNA1	CELSR1	-0.4056062	0.665527	1.0711332	1.0711332
SLC26A7	PRICKLE1	0.55810285	-0.5129919	-1.07109475	1.07109475
LTF	FLRT2	0.66645354	-0.40463325	-1.07108679	1.07108679
FKBP1A	GABBR1	-0.43757498	0.6334294	1.07100438	1.07100438
PUS1	CHD2	-0.42944214	0.6415374	1.07097954	1.07097954
POLR2C	ATPIF1	-0.5652275	0.5054623	1.0706898	1.0706898
XRCC5	WBP4	-0.39283657	0.67784417	1.07068074	1.07068074

MYOM1	GALNT2	-0.7358197	0.33484095	1.07066065	1.07066065
ABCG1	CREB3L4	-0.44388527	0.6267559	1.07064117	1.07064117
TRIM14	DDB2	-0.2566546	0.8139584	1.070613	1.070613
XRCC5	IGF2R	-0.62491566	0.44563824	1.0705539	1.0705539
PDE4DIP	RACGAP1	-0.5779714	0.4925445	1.0705159	1.0705159
PDE7A	SLC39A14	0.51850843	-0.5519056	-1.07041403	1.07041403
ITGB4	SVEP1	-0.7266399	0.34370974	1.07034964	1.07034964
ZBTB10	ADAMTS9	-0.45569625	0.61457556	1.07027181	1.07027181
CAPS2	ACTN3	0.51359653	-0.5566705	-1.07026703	1.07026703
TOP2A	NOP2	0.6007039	-0.4695227	-1.0702266	1.0702266
VCAM1	PINK1	0.55453825	-0.51567	-1.07020825	1.07020825
DCAF8	HPS6	-0.5539695	0.5162357	1.0702052	1.0702052
PRMT5	PCMTD1	-0.6645098	0.4055175	1.0700273	1.0700273
COG6	CLASRP	0.58974123	-0.4802644	-1.07000563	1.07000563
FAT3	EFEMP2	0.54242826	-0.5275369	-1.06996516	1.06996516
AK2	MRPS14	-0.22791012	0.8420352	1.06994532	1.06994532
ENG	RNF130	-0.5492465	0.5204883	1.0697348	1.0697348
ENO3	FHOD1	-0.42943713	0.64006054	1.06949767	1.06949767
SERPINA1	ESRRG	0.53184414	-0.53754646	-1.0693906	1.0693906
ADAMTS2	LPGAT1	0.58270866	-0.48662797	-1.06933663	1.06933663
ARPP21	KLF4	-0.6650582	0.40409097	1.06914917	1.06914917
CWF19L2	SDPR	0.5655303	-0.5035309	-1.0690612	1.0690612
MBD2	PSMB2	-0.51125914	0.55774206	1.0690012	1.0690012
FAM76A	GGCT	-0.7072844	0.3616575	1.0689419	1.0689419
MGAT5	CLASP2	-0.22452912	0.84421384	1.06874296	1.06874296
PSMB3	NAALADL2	-0.66128206	0.40726128	1.06854334	1.06854334
EIF5	KLF12	0.2871113	-0.7812882	-1.0683995	1.0683995
CTNNA1	MTHFD1L	-0.47259927	0.59575146	1.06835073	1.06835073
UBC	TBRG4	-0.6679893	0.40027618	1.06826548	1.06826548
MCM6	USP9X	-0.34620467	0.72205055	1.06825522	1.06825522
MRPS22	SNX18	-0.38691598	0.6812804	1.06819638	1.06819638
EGFL6	HOXB8	0.53581977	-0.532191	-1.06801077	1.06801077
TOR1AIP2	IRF4	-0.72461206	0.3433574	1.06796946	1.06796946
COPZ1	SEC62	-0.52076477	0.54713136	1.06789613	1.06789613
ACVR2A	AACS	-0.5391993	0.5286045	1.0678038	1.0678038
ORMDL1	NFIX	0.4454872	-0.6222913	-1.0677785	1.0677785
SLAMF1	ESRP2	0.7056767	-0.36139327	-1.06706997	1.06706997
TAOK3	ENTPD3	0.5386291	-0.52839655	-1.06702565	1.06702565
COPZ1	PDZD2	-0.5147214	0.55221814	1.06693954	1.06693954
CYR61	IGFBP4	0.47270188	-0.5941771	-1.06687898	1.06687898
SHQ1	COL5A1	-0.36673638	0.70004374	1.06678012	1.06678012
UBC	DHX30	-0.39483097	0.67189497	1.06672594	1.06672594
COPZ1	PDE7A	0.5476037	-0.51909316	-1.06669686	1.06669686
NEDD4L	MRPS22	-0.53117824	0.5353217	1.06649994	1.06649994
QSOX1	PCOLCE2	0.5139709	-0.5524722	-1.0664431	1.0664431
ELAC2	PIK3IP1	-0.37466615	0.69173986	1.06640601	1.06640601
COL5A1	HIST1H2BK	-0.43776783	0.62859696	1.06636479	1.06636479
POLR1C	NETO2	0.82346094	-0.24288219	-1.06634313	1.06634313
PALLD	MAP4	0.6354828	-0.43082902	-1.06631182	1.06631182
SERBP1	NETO2	0.84144926	-0.22464219	-1.06609145	1.06609145
CSTF3	COL15A1	-0.7668683	0.29919556	1.06606386	1.06606386
OPRK1	LDLRAD4	-0.61761147	0.4482097	1.06582117	1.06582117
PAX2	KLF12	-0.28146842	0.7843493	1.06581772	1.06581772
PPP1R14A	SEMA6A	-0.7028855	0.36281905	1.06570455	1.06570455
SERBP1	KLF12	0.39778247	-0.6678063	-1.06558877	1.06558877

NOP56	HDAC11	-0.5439455	0.5216429	1.0655884	1.0655884
RRBP1	KIF5B	0.6361553	-0.42942685	-1.06558215	1.06558215
NDRG2	JUND	0.66230136	-0.40307567	-1.06537703	1.06537703
VWA9	NTNG2	0.417406	-0.6479673	-1.0653733	1.0653733
NTRK2	LTF	-0.53837454	0.52691674	1.06529128	1.06529128
RBM45	MTHFSD	0.39024082	-0.6750295	-1.06527032	1.06527032
MAP1B	RPL14	-0.59971946	0.46552166	1.06524112	1.06524112
PGF	FAT3	-0.5201361	0.5449322	1.0650683	1.0650683
ADAMTS2	LAMA1	-0.5149006	0.5500705	1.0649711	1.0649711
GPHN	LCLAT1	-0.60920286	0.45559156	1.06479442	1.06479442
NTRK2	HSP90AA1	-0.6441401	0.4206457	1.0647858	1.0647858
SMYD3	AACS	-0.59811556	0.46663427	1.06474983	1.06474983
FOXN3	PSMB3	-0.6873873	0.37733114	1.06471844	1.06471844
DDX6	PHF8	-0.34950933	0.71517694	1.06468627	1.06468627
KIF11	NUSAP1	0.95173293	-0.11290682	-1.06463975	1.06463975
CEACAM1	SSFA2	-0.3781209	0.6864549	1.0645758	1.0645758
BUB1B	PSMB2	0.754086	-0.31043255	-1.06451855	1.06451855
RUFY3	PTDSS1	-0.62343454	0.44085333	1.06428787	1.06428787
HSD11B1	DBI	-0.5836648	0.48057094	1.06423574	1.06423574
SPAG5	PHF8	0.47505373	-0.5887844	-1.06383813	1.06383813
ITGB1	COL1A1	0.3337619	-0.7299039	-1.0636658	1.0636658
COL6A3	AK9	0.49269187	-0.57080215	-1.06349402	1.06349402
RUFY3	MPHOSPH6	-0.71511245	0.34836802	1.06348047	1.06348047
GALNT11	GALNT2	-0.7279422	0.33548272	1.06342492	1.06342492
XRCC5	RAB4A	-0.47375187	0.58955485	1.06330672	1.06330672
KIAA0101	MOCOS	0.71442765	-0.34855816	-1.06298581	1.06298581
LMO3	COL1A1	0.16715042	-0.8956476	-1.06279802	1.06279802
MID1	LY75	0.71817267	-0.34461537	-1.06278804	1.06278804
KIF13A	TBRG4	-0.5868999	0.47588605	1.06278595	1.06278595
SS18L2	RRN3	-0.45749113	0.60527056	1.06276169	1.06276169
ESRRG	ITIH5	-0.25507173	0.8076115	1.06268323	1.06268323
NOD1	VARS2	-0.34215784	0.72048336	1.0626412	1.0626412
XRCC5	POU2F2	0.2860276	-0.7765573	-1.0625849	1.0625849
SOX4	EIF4E3	0.31149778	-0.7510355	-1.06253328	1.06253328
ZWINT	SCML2	0.4276383	-0.6348283	-1.0624666	1.0624666
PALLD	FOXJ2	-0.28776047	0.7746882	1.06244867	1.06244867
HNRNPA1	PRRC2A	-0.5642691	0.49814156	1.06241066	1.06241066
CALD1	NETO2	-0.59043515	0.4718964	1.06233155	1.06233155
CEACAM1	PDZD2	-0.34337717	0.71880114	1.06217831	1.06217831
CDK6	MGEA5	-0.48651007	0.5755889	1.06209897	1.06209897
HSP90AA1	HNRNPC	0.4835374	-0.57838184	-1.06191924	1.06191924
STS	ITGA8	0.7637229	-0.298102	-1.0618249	1.0618249
KCND2	GALNT2	-0.52649015	0.53533286	1.06182301	1.06182301
ZFR	CAMK2N1	-0.60228723	0.45934638	1.06163361	1.06163361
SOD2	KLF9	-0.5200706	0.54132676	1.06139736	1.06139736
PPA1	NDN	-0.5993299	0.46205953	1.06138943	1.06138943
TOP2A	COPZ1	0.5398217	-0.52155703	-1.06137873	1.06137873
PPA1	LRP8	0.8353481	-0.22602865	-1.06137675	1.06137675
FLNB	SLC24A4	-0.35252798	0.70883065	1.06135863	1.06135863
KCNK3	PDLIM4	-0.60294104	0.45831183	1.06125287	1.06125287
QSOX1	RMDN1	0.43693984	-0.6242792	-1.06121904	1.06121904
NEDD4L	ARPC5L	-0.71472716	0.34648997	1.06121713	1.06121713
SCARB2	DNAJC9	-0.7732841	0.28791463	1.06119873	1.06119873
EPHA1	RCC2	0.61260694	-0.44853014	-1.06113708	1.06113708
GSTT2	IP6K2	-0.5746559	0.4863165	1.0609724	1.0609724

LPIN1	CDK6	0.36278263	-0.6981239	-1.06090653	1.06090653
BCS1L	SPERT	0.5736686	-0.48694137	-1.06060997	1.06060997
TRAP1	APP	-0.42484367	0.6357621	1.06060577	1.06060577
CAP2	TDRKH	-0.7993832	0.26121444	1.06059764	1.06059764
SERBP1	IRF9	-0.5328789	0.5276755	1.0605544	1.0605544
ZWINT	GDAP2	-0.4487793	0.61167043	1.06044973	1.06044973
MOCOS	SHMT2	0.696562	-0.36385238	-1.06041438	1.06041438
TNC	THBS1	0.48590118	-0.57446855	-1.06036973	1.06036973
DTL	DCAF7	-0.4971641	0.56317335	1.06033745	1.06033745
XPO1	TUSC3	-0.3523818	0.70790935	1.06029115	1.06029115
SUFU	CA8	0.3113392	-0.7487991	-1.0601383	1.0601383
LTA	MPHOSPH6	0.50067705	-0.5594571	-1.06013415	1.06013415
CXCR4	COLGALT1	-0.60381263	0.45630857	1.0601212	1.0601212
PDHB	RNF150	-0.38664997	0.67345774	1.06010771	1.06010771
EPS8	FANCI	-0.6969327	0.36316445	1.06009715	1.06009715
KCND2	CBLN2	0.33125055	-0.7288406	-1.06009115	1.06009115
NXF1	NUP188	-0.30831832	0.75166076	1.05997908	1.05997908
COL5A1	AGTRAP	-0.31809118	0.741767	1.05985818	1.05985818
TYRO3	PRICKLE1	0.34964362	-0.7101503	-1.05979392	1.05979392
DYSF	ENTHD2	-0.5185474	0.54112077	1.05966817	1.05966817
MRPS33	S100A8	-0.4284557	0.6311604	1.0596161	1.0596161
LIMK2	SAFB2	-0.453538	0.6059376	1.0594756	1.0594756
GRWD1	FOXP2	-0.58140016	0.47807494	1.0594751	1.0594751
PCNA	CCDC90B	-0.28364736	0.7757734	1.05942076	1.05942076
SOX4	FAM216A	-0.6074165	0.45187432	1.05929082	1.05929082
PPA1	CA8	-0.5057643	0.5533278	1.0590921	1.0590921
EIF4G3	BRAP	0.48156443	-0.57743734	-1.05900177	1.05900177
CSNK1G2	CDK10	-0.22675765	0.8322353	1.05899295	1.05899295
TOR1AIP1	CWF19L2	0.46786803	-0.5910845	-1.05895253	1.05895253
AKAP12	QSOX1	0.4534167	-0.6055249	-1.0589416	1.0589416
ESRRG	QKI	-0.34581143	0.71304744	1.05885887	1.05885887
STXBP6	MTHFR	0.5075027	-0.55116963	-1.05867233	1.05867233
CD40	ZFAT	0.54467595	-0.5139621	-1.05863805	1.05863805
CYBRD1	GSTM5	0.7474644	-0.3110563	-1.0585207	1.0585207
SRD5A1	RGS5	-0.7076097	0.35085395	1.05846365	1.05846365
CNKSR2	ARHGEF12	-0.700485	0.357958	1.058443	1.058443
MAGI1	IDNK	0.5370241	-0.5213942	-1.0584183	1.0584183
SERBP1	TM9SF3	-0.56466675	0.49374327	1.05841002	1.05841002
RAB35	XXYLT1	0.5911519	-0.46720654	-1.05835844	1.05835844
ZNF385D	MZF1	-0.6569045	0.40145063	1.05835513	1.05835513
PDIA4	LRWD1	0.5395261	-0.51876646	-1.05829256	1.05829256
XRCC5	SCARB2	-0.389368	0.66892433	1.05829233	1.05829233
ESRRG	ARHGEF6	-0.32009378	0.738088	1.05818178	1.05818178
CHPF	HELLS	-0.5650823	0.49297008	1.05805238	1.05805238
LOC145783	CHD1L	-0.4630601	0.5948575	1.0579176	1.0579176
DPT	SSB	-0.3243788	0.73349684	1.05787564	1.05787564
ANXA11	ELOVL5	-0.58424765	0.47362718	1.05787483	1.05787483
PINK1-AS	IRF1	0.50855565	-0.54918295	-1.0577386	1.0577386
CSF3R	POLN	0.77685916	-0.28087813	-1.05773729	1.05773729
ZNF608	ZNF358	0.5461425	-0.51144046	-1.05758296	1.05758296
GNAL	SULF2	0.39457476	-0.6629661	-1.05754086	1.05754086
SOAT1	PNN	-0.2505726	0.8069295	1.0575021	1.0575021
CEP68	PLD5	0.6101613	-0.44726828	-1.05742958	1.05742958
ZNF467	C11orf63	0.5066055	-0.5506789	-1.0572844	1.0572844
KLF12	MGEA5	-0.15438832	0.9028337	1.05722202	1.05722202

MIR3917	NUDT21	0.69483787	-0.3623577	-1.05719557	1.05719557
TRA2B	COLGALT1	0.40071094	-0.65647113	-1.05718207	1.05718207
SMAD7	CADM1	-0.3598246	0.69732666	1.05715126	1.05715126
SOD2	IL32	0.606067	-0.45107317	-1.05714017	1.05714017
SOCS3	TEAD4	0.6524287	-0.40460655	-1.05703525	1.05703525
MAP1B	TRIM14	0.56764406	-0.4892745	-1.05691856	1.05691856
CDK6	CHURC1-FNTB	-0.47587612	0.58088917	1.05676529	1.05676529
CST2	RCC2	0.4302196	-0.6264897	-1.0567093	1.0567093
IPO11	ELOVL6	-0.6548725	0.4017521	1.0566246	1.0566246
MCM6	CBX3	-0.23152962	0.8249951	1.05652472	1.05652472
TXNL1	SFN	0.4996491	-0.5567894	-1.0564385	1.0564385
HTT	OPRK1	-0.63296664	0.42336932	1.05633596	1.05633596
RACGAP1	ARHGEF2	-0.72430295	0.33197173	1.05627468	1.05627468
ARNTL	RRBP1	-0.52673286	0.5294475	1.05618036	1.05618036
CADM1	KLHL5	-0.503979	0.5519424	1.0559214	1.0559214
EIF2B2	SDPR	-0.43458685	0.62129796	1.05588481	1.05588481
SLIRP	MSRB2	-0.53900146	0.5168724	1.05587386	1.05587386
GSTP1	CXCL1	0.5178807	-0.5379426	-1.0558233	1.0558233
S100B	NPC1	0.4037654	-0.65197974	-1.05574514	1.05574514
CSK	DDB2	-0.33645743	0.71927327	1.0557307	1.0557307
NOL8	MAN2B2	-0.3443906	0.7111389	1.0555295	1.0555295
RELB	NUDT21	0.44620797	-0.6092139	-1.05542187	1.05542187
PPP2CB	ESRRG	-0.41394582	0.64143634	1.05538216	1.05538216
CCNI	TUSC5	0.67978764	-0.3755641	-1.05535174	1.05535174
GSTM5	ZHX3	0.73642725	-0.31890857	-1.05533582	1.05533582
ANPEP	CHEK2	-0.20598373	0.84924364	1.05522737	1.05522737
GMPS	RAE1	0.6628849	-0.39232516	-1.05521006	1.05521006
BID	BCL2	-0.6409309	0.41418755	1.05511845	1.05511845
MOB3B	ZDHHC14	0.59123564	-0.46381873	-1.05505437	1.05505437
LTA	CD40	0.601374	-0.45353138	-1.05490538	1.05490538
NAP1L1	BID	-0.6549522	0.39993054	1.05488274	1.05488274
SH3PXD2A	PDLIM4	-0.5195822	0.53528446	1.05486666	1.05486666
COL15A1	BRAP	-0.4160169	0.6387723	1.0547892	1.0547892
MMD	COL5A1	0.65078276	-0.40392917	-1.05471193	1.05471193
BRD8	FAM43A	-0.7272849	0.32736254	1.05464744	1.05464744
PALLD	CLRN1	-0.49729368	0.55705845	1.05435213	1.05435213
UBC	PRR11	-0.614702	0.43961152	1.05431352	1.05431352
RBM10	RELA	-0.35043028	0.70381635	1.05424663	1.05424663
SEC62	NAB2	0.4987085	-0.5555234	-1.0542319	1.0542319
ADAM10	KCNE3	0.36800933	-0.6861812	-1.05419053	1.05419053
GPLD1	ABCG1	0.5192137	-0.53493816	-1.05415186	1.05415186
HNRNPL	SNRPD3	0.34499562	-0.70912737	-1.05412299	1.05412299
SETD7	FZD10	0.27529296	-0.778769	-1.05406196	1.05406196
SOX4	KIAA1033	0.5582844	-0.495518	-1.0538024	1.0538024
LTF	PRR14L	0.43856344	-0.6150978	-1.05366124	1.05366124
ANAPC5	COPRS	0.53447485	-0.5191046	-1.05357945	1.05357945
MLXIP	CNPY2	-0.36125126	0.692223	1.05347426	1.05347426
KCNK3	RPL15	-0.32752368	0.72585464	1.05337832	1.05337832
TCF3	LCMT2	0.60222477	-0.45112443	-1.0533492	1.0533492
ACVR2A	BGN	0.6797847	-0.37340105	-1.05318575	1.05318575
XPO1	APEX2	-0.59609854	0.4567284	1.05282694	1.05282694
CD1D	TMEM44	-0.411387	0.64143	1.052817	1.052817
XRCC5	KANK1	-0.69177175	0.3609746	1.05274635	1.05274635
PTPN11	CADM1	-0.60741264	0.4453332	1.05274584	1.05274584
ARHGEF12	KAT2A	0.53038126	-0.5223104	-1.05269166	1.05269166

BID	ATF6B	0.6683252	-0.38436595	-1.05269115	1.05269115
CADM1	SULF2	0.41755238	-0.6349583	-1.05251068	1.05251068
MCM4	CDC7	0.6092091	-0.44325283	-1.05246193	1.05246193
SH3RF1	TBRG4	-0.42614567	0.6262359	1.05238157	1.05238157
PDE4DIP	PDHB	-0.3073981	0.74493796	1.05233606	1.05233606
SIRPA	EZH2	-0.35275298	0.699506	1.05225898	1.05225898
MED19	MED16	-0.37384227	0.6783539	1.05219617	1.05219617
MMS19	NUP188	-0.5617481	0.4904292	1.0521773	1.0521773
EPS15	SNRPB2	-0.41180673	0.6403269	1.05213363	1.05213363
SORL1	SHANK2	0.5011979	-0.5509091	-1.052107	1.052107
CADM1	NMT2	-0.38732263	0.6647593	1.05208193	1.05208193
PDIA4	CLSTN1	-0.21075134	0.8411412	1.05189254	1.05189254
KIF5B	DYSF	0.60151047	-0.45036313	-1.0518736	1.0518736
CALD1	GABBR1	-0.45572054	0.5961504	1.05187094	1.05187094
SOBP	NEGR1	-0.44301862	0.608837	1.05185562	1.05185562
PCNA	HLA-B	-0.36643443	0.6853435	1.05177793	1.05177793
CNTNAP1	EGR1	0.24515824	-0.8065911	-1.05174934	1.05174934
TK1	EZH2	0.7490872	-0.30250186	-1.05158906	1.05158906
SOD3	MRPL14	-0.6772582	0.37421054	1.05146874	1.05146874
RAB35	RAB11A	0.7514887	-0.29974625	-1.05123495	1.05123495
PIAS2	PSMA4	-0.41506597	0.636161	1.05122697	1.05122697
KLF12	ADARB1	0.34229413	-0.7087858	-1.05107993	1.05107993
NXF1	NUP62	-0.4826116	0.5684447	1.0510563	1.0510563
TNFRSF21	BST2	-0.69831544	0.35246345	1.05077889	1.05077889
TRIM23	SEC61A1	0.46398365	-0.5867762	-1.05075985	1.05075985
SYCP2	AGPAT5	-0.4788812	0.57174605	1.05062725	1.05062725
ADA	LUC7L3	-0.4742921	0.5760813	1.0503734	1.0503734
ESRRG	GRB14	-0.27712205	0.77323735	1.0503594	1.0503594
BCL2	PRICKLE1	0.2665354	-0.78372824	-1.05026364	1.05026364
ASS1	GTF2I	-0.30180913	0.7484409	1.05025003	1.05025003
KARS	CSDE1	-0.5289772	0.5211677	1.0501449	1.0501449
CCT3	ADGRA2	-0.538114	0.5119274	1.0500414	1.0500414
MCM6	PCED1B	-0.6652462	0.38476378	1.05000998	1.05000998
PGRMC2	QSOX1	0.39224347	-0.6577087	-1.04995217	1.04995217
PAPSS1	SSB	0.55446607	-0.49533793	-1.049804	1.049804
MRPS14	PHF5A	-0.43329734	0.6164956	1.04979294	1.04979294
ZFP36L1	ACACA	0.27449283	-0.7751763	-1.04966913	1.04966913
TBC1D9B	PRICKLE1	0.47176006	-0.5776979	-1.04945796	1.04945796
GNAL	PDHB	-0.4774985	0.57179093	1.04928943	1.04928943
MFAP3	LMOD3	0.5492044	-0.49990308	-1.04910748	1.04910748
COL9A1	CNTNAP1	0.52074915	-0.5281838	-1.04893295	1.04893295
ZNF202	MZF1	-0.3873601	0.66122246	1.04858256	1.04858256
FDPS	IL17A	0.29252443	-0.756044	-1.04856843	1.04856843
SRPRB	NDN	-0.60432875	0.44418642	1.04851517	1.04851517
ENOSF1	C19orf25	-0.7200807	0.32843304	1.04851374	1.04851374
CHD2	JDP2	0.33857828	-0.7098905	-1.04846878	1.04846878
MFAP3	VLDLR	-0.34317157	0.7052399	1.04841147	1.04841147
COL15A1	PALLD	0.44554472	-0.60267806	-1.04822278	1.04822278
MFAP3	AHI1	0.7251813	-0.32302037	-1.04820167	1.04820167
DCUN1D5	ZNF608	-0.23773271	0.81041855	1.04815126	1.04815126
WARS	CD48	0.49212626	-0.5558259	-1.04795216	1.04795216
CEACAM1	CCDC90B	-0.72654366	0.32124338	1.04778704	1.04778704
VLDLR	THBS3	0.84219384	-0.20549887	-1.04769271	1.04769271
SGCB	GSTM5	0.65492535	-0.39276636	-1.04769171	1.04769171
KCND2	TIMM23	-0.4389272	0.60861665	1.04754385	1.04754385

KARS	NAALADL2	-0.4804996	0.5668296	1.0473292	1.0473292
FOXJ2	TMEM159	-0.39228848	0.6550155	1.04730398	1.04730398
MFAP3	PCOLCE2	-0.56945455	0.47775447	1.04720902	1.04720902
EIF4G3	GPAT4	-0.6514671	0.395676	1.0471431	1.0471431
KIAA0391	TMEM55A	-0.6127537	0.43436942	1.04712312	1.04712312
STK10	SLC7A2	-0.28649062	0.760574	1.04706462	1.04706462
MFAP3	SPAG16	-0.49868765	0.54835427	1.04704192	1.04704192
HDAC1	GTF2I	-0.7143855	0.33260393	1.04698943	1.04698943
FGFR1OP	KLF8	-0.5015241	0.5452778	1.0468019	1.0468019
PPIB	HNRNPL	0.5447583	-0.50198734	-1.04674564	1.04674564
GNAQ	TRIM2	0.41882876	-0.62769246	-1.04652122	1.04652122
ORMDL1	DPAGT1	-0.15945718	0.8870628	1.04651998	1.04651998
LARP6	CACNB3	0.47321734	-0.5732762	-1.04649354	1.04649354
CAB39	OLMALINC	-0.51176226	0.534677	1.04643926	1.04643926
HSP90AA1	PPIB	0.52669966	-0.5197287	-1.04642836	1.04642836
COPZ1	NMT2	-0.5477581	0.49856973	1.04632783	1.04632783
KPNA4	CELSR1	0.3136188	-0.7326517	-1.0462705	1.0462705
NDFIP2	HSPB8	-0.5175481	0.5286341	1.0461822	1.0461822
TXNIP	SMAD7	-0.48638016	0.55969864	1.0460788	1.0460788
PARK2	QDPR	-0.4740202	0.5719785	1.0459987	1.0459987
DHX30	PARP14	-0.41891614	0.6270429	1.04595904	1.04595904
DIEXF	SLC27A3	-0.28055808	0.7653786	1.04593668	1.04593668
COPZ1	ZNF608	-0.24882361	0.79709685	1.04592046	1.04592046
GNAL	ADAMTS3	0.30518827	-0.7407072	-1.04589547	1.04589547
C14orf169	CHD2	-0.623068	0.4228157	1.0458837	1.0458837
ENG	LMOD3	0.35931674	-0.6864625	-1.04577924	1.04577924
QKI	MOCOS	-0.59548956	0.45025384	1.0457434	1.0457434
KIF5B	CDCA7	0.30175483	-0.74394983	-1.04570466	1.04570466
GRB14	MLST8	0.5475147	-0.49806637	-1.04558107	1.04558107
FAM129B	TIMM23	0.4257001	-0.61983716	-1.04553726	1.04553726
ARPP21	DFNB31	0.5369075	-0.50839543	-1.04530293	1.04530293
GFOD1	STAT3	0.39264286	-0.65258837	-1.04523123	1.04523123
ZMYND11	GNPTAB	-0.67343086	0.37175542	1.04518628	1.04518628
SLC26A7	MMP15	0.7468732	-0.29815334	-1.04502654	1.04502654
KIRREL	AAGAB	-0.47012866	0.5748902	1.04501886	1.04501886
ARPP21	KLHL5	-0.7031152	0.3417744	1.0448896	1.0448896
DCN	MAP6	0.6892586	-0.35560963	-1.04486823	1.04486823
SNRPB2	NFIA	-0.5310772	0.51375294	1.04483014	1.04483014
NAB1	RACGAP1	-0.44849393	0.59631956	1.04481349	1.04481349
PDE4DIP	DCUN1D5	-0.32123178	0.72346514	1.04469692	1.04469692
FAM13A	QSOX1	0.34101415	-0.7036178	-1.04463195	1.04463195
DHX30	LHPP	0.5381742	-0.50640064	-1.04457484	1.04457484
TAOK3	MAPK13	-0.55625266	0.48827466	1.04452732	1.04452732
GNA12	CCDC117	0.53229994	-0.51215833	-1.04445827	1.04445827
TBC1D1	FOS	0.6649155	-0.3793902	-1.0443057	1.0443057
CBX3	DDX39A	-0.2496942	0.7946061	1.0443003	1.0443003
GSTM5	NMNAT3	0.6613754	-0.38283393	-1.04420933	1.04420933
UBC	NFATC2IP	-0.7758697	0.26826587	1.04413557	1.04413557
CCDC3	ZNF804A	-0.52149564	0.52260965	1.04410529	1.04410529
NAP1L1	CD40	-0.6689553	0.37472323	1.04367853	1.04367853
COX7A1	PRICKLE1	0.38136372	-0.6622498	-1.04361352	1.04361352
B4GALT1	EFCAB14	-0.47539866	0.568168	1.04356666	1.04356666
UBE2B	DLC1	-0.40951625	0.6340003	1.04351655	1.04351655
BLOC1S6	ABI2	0.50106245	-0.54237187	-1.04343432	1.04343432
DSG2	LAP3	-0.561092	0.48223433	1.04332633	1.04332633

VEGFA	PFDN4	0.3751019	-0.66815436	-1.04325626	1.04325626
COL1A1	SDC2	0.40228355	-0.6409002	-1.04318375	1.04318375
RBM17	SOX4	0.5145349	-0.5285694	-1.0431043	1.0431043
SPEF2	HDAC1	0.32791173	-0.71518135	-1.04309308	1.04309308
NAP1L1	GGCT	-0.47741047	0.565671	1.04308147	1.04308147
PGRMC2	COL5A1	0.5299159	-0.513119	-1.0430349	1.0430349
XRCC5	DSG2	-0.4345746	0.60840905	1.04298365	1.04298365
KARS	COL15A1	-0.628381	0.41450608	1.04288708	1.04288708
TUSC3	LOC10099669	0.26444727	-0.77832395	-1.04277122	1.04277122
ZHX2	CEP68	0.5992194	-0.44353366	-1.04275306	1.04275306
MFAP3	RPRD1A	-0.3977638	0.6449698	1.0427336	1.0427336
PTPRG	RANGAP1	0.28849638	-0.75415957	-1.04265595	1.04265595
MYOM1	ARPC5L	-0.67755723	0.36501363	1.04257086	1.04257086
CBX3	NOP56	-0.47170082	0.570689	1.04238982	1.04238982
WDR46	FAM111A	-0.34244785	0.6998829	1.04233075	1.04233075
ISCU	HDAC1	-0.69876736	0.34350276	1.04227012	1.04227012
TBL1XR1	ZNF467	0.61419743	-0.42797476	-1.04217219	1.04217219
AGTRAP	SIRPA	-0.28929263	0.75282866	1.04212129	1.04212129
TRIM2	RAB5A	-0.38376266	0.65829146	1.04205412	1.04205412
PUS1	PFDN4	0.4085287	-0.6334686	-1.0419973	1.0419973
NGFR	TMOD2	-0.39564335	0.64618427	1.04182762	1.04182762
NAALADL2	SNRPC	-0.70690274	0.3349186	1.04182134	1.04182134
IL18	HIVEP3	0.5012096	-0.540407	-1.0416166	1.0416166
FGFR2	PRMT8	-0.7113129	0.3302864	1.0415993	1.0415993
GYS1	MCM3	-0.5137019	0.5278243	1.0415262	1.0415262
LNPEP	MEST	-0.48771605	0.553736	1.04145205	1.04145205
TRMT13	MTHFD1L	-0.46659797	0.57477415	1.04137212	1.04137212
TAOK3	AGPAT5	-0.45886242	0.58244586	1.04130828	1.04130828
CSNK2A1	FAM171A1	-0.5112556	0.5300114	1.041267	1.041267
CCT3	TSPAN9	0.5441426	-0.4970458	-1.0411884	1.0411884
POC1A	WRAP53	0.5938008	-0.44731456	-1.04111536	1.04111536
DCUN1D5	HLA-C	-0.44687223	0.5940498	1.04092203	1.04092203
ZFP36L1	WDR27	-0.31012595	0.7307512	1.04087715	1.04087715
SNRPB2	SCARB2	-0.42779824	0.613042	1.04084024	1.04084024
SEPT11	PARP14	0.3131131	-0.7276701	-1.0407832	1.0407832
CAST	ABI2	0.35160077	-0.6891022	-1.04070297	1.04070297
KPNA4	LIF	0.31533054	-0.7251153	-1.04044584	1.04044584
APC	STX17	-0.4837778	0.5566598	1.0404376	1.0404376
UBC	MRPL9	-0.4282755	0.61211324	1.04038874	1.04038874
ARHGEF12	TSPAN9	0.66705835	-0.37325087	-1.04030922	1.04030922
PRICKLE1	LIMCH1	0.29907086	-0.741181	-1.04025186	1.04025186
GLRB	SLC25A29	0.41750827	-0.62270796	-1.04021623	1.04021623
UBC	ZWILCH	-0.45063546	0.5895613	1.04019676	1.04019676
NXF1	AMMECR1	0.39274746	-0.6473658	-1.04011326	1.04011326
NOL10	INIP	0.5997481	-0.44035807	-1.04010617	1.04010617
YARS	THRB	-0.5472102	0.4928512	1.0400614	1.0400614
DCUN1D5	UCK2	0.4070761	-0.6329758	-1.0400519	1.0400519
FHOD1	PHF8	-0.19157404	0.8483885	1.03996254	1.03996254
MLST8	ETV3	0.4143207	-0.62559277	-1.03991347	1.03991347
MAP4	PRICKLE1	0.4514179	-0.5884929	-1.0399108	1.0399108
ATG2A	EGR1	0.49253404	-0.5471933	-1.03972734	1.03972734
FKBP1A	CXCL2	0.6416518	-0.39799577	-1.03964757	1.03964757
ELOVL6	TNS1	0.7976853	-0.24186252	-1.03954782	1.03954782
KLF12	DDX52	0.27579054	-0.7637471	-1.03953764	1.03953764
ENO3	APP	-0.54132617	0.49819472	1.03952089	1.03952089

PDZRN3	STX6	-0.70179147	0.33764938	1.03944085	1.03944085
SSB	IL20RA	-0.5957018	0.44373775	1.03943955	1.03943955
XRCC5	CBX3	-0.46476158	0.57459176	1.03935334	1.03935334
TEX264	C8orf33	-0.3618476	0.6774701	1.0393177	1.0393177
AIMP1	PFDN4	0.6637982	-0.37550443	-1.03930263	1.03930263
LPCAT3	GRM5	0.29389578	-0.745328	-1.03922378	1.03922378
SERBP1	NAB1	-0.492146	0.5470439	1.0391899	1.0391899
KLF9	PCBP2	0.4381724	-0.6008576	-1.03903	1.03903
NEGR1	RNF207	0.3447052	-0.69423765	-1.03894285	1.03894285
KLF12	TIMELESS	0.39968038	-0.6392183	-1.03889868	1.03889868
TOP2A	GART	0.47573778	-0.56289303	-1.03863081	1.03863081
UBC	NUP62	-0.48331717	0.5552413	1.03855847	1.03855847
GEM	ADGRA2	0.84813964	-0.19013816	-1.0382778	1.0382778
PFDN4	ZFAT	-0.30514	0.732819	1.037959	1.037959
PDIA4	PDE7A	0.5145663	-0.52337146	-1.03793776	1.03793776
APC	KANSL2	-0.5523659	0.4855669	1.0379328	1.0379328
ZMYND11	NLN	-0.6781927	0.35972685	1.03791955	1.03791955
MYOC	NAB2	0.44379443	-0.59403217	-1.0378266	1.0378266
CELSR1	RSL1D1	0.5039399	-0.53367823	-1.03761813	1.03761813
SNRPE	LPCAT2	0.2599312	-0.7775508	-1.037482	1.037482
AIDA	ZNF467	0.73784983	-0.2994327	-1.03728253	1.03728253
RPS20	NAB2	0.5995236	-0.43772703	-1.03725063	1.03725063
ZWINT	ZHX2	-0.55164146	0.48557505	1.03721651	1.03721651
KIF5B	COLGALT1	0.6077021	-0.4294876	-1.0371897	1.0371897
PPA1	NPY1R	-0.32389528	0.71316755	1.03706283	1.03706283
GNAQ	FUT11	-0.3656538	0.67132443	1.03697823	1.03697823
FADD	ABCA1	-0.475611	0.56128794	1.03689894	1.03689894
PCNA	TEFM	-0.5488623	0.48797062	1.03683292	1.03683292
CHD1L	MRPL50	-0.69399107	0.34282964	1.03682071	1.03682071
CIAO1	DCAF8	-0.49166837	0.54513663	1.036805	1.036805
ANPEP	NVL	-0.7225654	0.31420976	1.03677516	1.03677516
ATP5G1	ELK1	0.6416416	-0.39504275	-1.03668435	1.03668435
NDN	TRO	0.50662	-0.5299099	-1.0365299	1.0365299
CCT3	NAALADL2	-0.5647555	0.47177148	1.03652698	1.03652698
UCK2	SLC25A29	-0.7492678	0.2872406	1.0365084	1.0365084
ZEB1	CEP68	0.7654562	-0.27102298	-1.03647918	1.03647918
GLRB	POPDC3	-0.46629128	0.570133	1.03642428	1.03642428
NEDD4L	WARS	-0.45386338	0.58227366	1.03613704	1.03613704
CELSR1	SAMD4A	0.5183231	-0.5175415	-1.0358646	1.0358646
CHD2	AIDA	0.48558083	-0.5502739	-1.03585473	1.03585473
QSOX1	EHBP1	0.5051014	-0.53072274	-1.03582414	1.03582414
CCDC90B	TBC1D1	-0.42032644	0.6154796	1.03580604	1.03580604
AQP4	INF2	-0.27603653	0.75969374	1.03573027	1.03573027
RBM17	PRR11	-0.7653841	0.27030256	1.03568666	1.03568666
LMOD3	DNAJC9	0.42174283	-0.6138938	-1.03563663	1.03563663
TEX264	B3GALT6	-0.30114	0.73442364	1.03556364	1.03556364
IGF2R	CALD1	0.60491306	-0.43061996	-1.03553302	1.03553302
PLA2G6	MTHFR	-0.43202198	0.6034918	1.03551378	1.03551378
COG6	EIF4E3	-0.67340034	0.36202094	1.03542128	1.03542128
ENOSF1	RAB3IP	0.502487	-0.53282756	-1.03531456	1.03531456
TDRKH	SIRPA	-0.6608403	0.37436622	1.03520652	1.03520652
CBX3	XPO5	-0.15654816	0.87864614	1.0351943	1.0351943
WDR59	ELP6	0.41266167	-0.6225241	-1.03518577	1.03518577
DDX6	PRMT8	-0.50367117	0.53148115	1.03515232	1.03515232
CYBRD1	EFEMP2	0.8488493	-0.18618727	-1.03503657	1.03503657

KLF12	TIAL1	0.12928444	-0.9057491	-1.03503354	1.03503354
FAM43A	MZF1	-0.47880104	0.55618066	1.0349817	1.0349817
SPAG16	CIAO1	-0.64560777	0.38934416	1.03495193	1.03495193
KMT2E	RTP4	0.62693214	-0.40790692	-1.03483906	1.03483906
HNRNPA1	KSR1	-0.36348936	0.67134404	1.0348334	1.0348334
SGCB	TDRKH	-0.7395721	0.2950623	1.0346344	1.0346344
ECM2	FOPNL	-0.45931375	0.5751916	1.03450535	1.03450535
LRRC16A	LZTS1	-0.5200607	0.51440674	1.03446744	1.03446744
ADGRA2	DDX28	-0.65620947	0.37822583	1.0344353	1.0344353
C10orf11	NETO2	-0.6356633	0.39873466	1.03439796	1.03439796
TSPAN9	CNOT11	0.60545003	-0.42890885	-1.03435888	1.03435888
SPEF2	PUS1	0.27356523	-0.7607524	-1.03431763	1.03431763
COL15A1	PNPT1	-0.5443172	0.48986238	1.03417958	1.03417958
ADAM19	B4GALT6	0.6007335	-0.4332813	-1.0340148	1.0340148
ZWINT	LDLRAD4	-0.7026666	0.3311873	1.0338539	1.0338539
FGF18	EIF4E3	0.55959564	-0.47419903	-1.03379467	1.03379467
CTNNA1	POLR2H	-0.3523461	0.68129164	1.03363774	1.03363774
GABPB1-AS1	RELA	-0.65619236	0.3774069	1.03359926	1.03359926
TRIM27	KANSL1	-0.72186345	0.31165707	1.03352052	1.03352052
MOCS2	SPIRE2	-0.7578467	0.2756572	1.0335039	1.0335039
SET	SOX4	-0.6915944	0.3418464	1.0334408	1.0334408
AFF3	GALNT2	-0.58811027	0.4452732	1.03338347	1.03338347
FAM204A	GTPBP8	-0.59882	0.4344991	1.0333191	1.0333191
ANKRD28	TEAD4	-0.51011187	0.52319604	1.03330791	1.03330791
PDE5A	DCN	0.611574	-0.4217225	-1.0332965	1.0332965
MCM3	CBX3	-0.28231075	0.7509167	1.03322745	1.03322745
BUB1B	PRR11	0.92271215	-0.11049035	-1.0332025	1.0332025
KIF13A	TEC	0.40670556	-0.6264632	-1.03316876	1.03316876
MCM4	RACGAP1	0.82585716	-0.20700018	-1.03285734	1.03285734
APC	C19orf25	-0.35791075	0.6749284	1.03283915	1.03283915
TCF3	SEH1L	0.6875104	-0.34528542	-1.03279582	1.03279582
ASB1	MRPS22	-0.4069531	0.6258296	1.0327827	1.0327827
DCN	KANK1	0.7766999	-0.2558911	-1.032591	1.032591
SRPRB	TCEA3	-0.82368064	0.20877823	1.03245887	1.03245887
UBE2B	AHCYL1	0.8783563	-0.15404168	-1.03239798	1.03239798
LMO3	KCNQ4	-0.3001947	0.7321482	1.0323429	1.0323429
ABI2	SREK1IP1	0.5666971	-0.46564087	-1.03233797	1.03233797
UBC	C1orf131	-0.31514254	0.7171173	1.03225984	1.03225984
IGF1R	MIR503	-0.4806599	0.55153584	1.03219574	1.03219574
MMP14	CRISPLD2	-0.48225474	0.5499379	1.03219264	1.03219264
PPIA	HLA-C	-0.36266574	0.66943187	1.03209761	1.03209761
NFIA	PSMG3	-0.66287035	0.36913937	1.03200972	1.03200972
NFASC	MAGOHB	-0.653534	0.37844336	1.03197736	1.03197736
MCM6	PDE7A	0.49507388	-0.53680456	-1.03187844	1.03187844
MMD	CERS5	0.58359927	-0.44826642	-1.03186569	1.03186569
POLR2D	SPAG5	0.45957318	-0.5722736	-1.03184678	1.03184678
CREBL2	RAB11A	-0.4663718	0.5654246	1.0317964	1.0317964
ADAMTS9	RAB13	0.72674745	-0.30500972	-1.03175717	1.03175717
VCAM1	ARHGEF37	0.6025595	-0.42916957	-1.03172907	1.03172907
RRM1	FARSA	-0.2662156	0.765486	1.0317016	1.0317016
STAT5B	C8orf4	-0.5565181	0.4750577	1.0315758	1.0315758
PALLD	URI1	-0.4685031	0.56289935	1.03140245	1.03140245
ZNF467	ZNF668	-0.32876667	0.70259833	1.031365	1.031365
OLMALINC	SERPINE2	-0.5921834	0.4390673	1.0312507	1.0312507
TSPAN9	TSKU	-0.6240109	0.4072222	1.0312331	1.0312331

ESRRG	FOXN3	-0.5227986	0.5083067	1.0311053	1.0311053
GMPS	SSBP2	-0.21834011	0.81268436	1.03102447	1.03102447
LHPP	PIKFYVE	-0.49753594	0.5332293	1.03076524	1.03076524
DPT	CELSR1	0.3486929	-0.68205345	-1.03074635	1.03074635
KLF12	ADPGK	0.2211915	-0.8095449	-1.0307364	1.0307364
MRPL17	CAB39L	-0.8894879	0.14118452	1.03067242	1.03067242
XRCC5	TMEM229B	0.776413	-0.25419906	-1.03061206	1.03061206
MMS19	JDP2	0.42286512	-0.6076294	-1.03049452	1.03049452
ZNF385D	GFRA4	0.5188879	-0.5114443	-1.0303322	1.0303322
POLR2C	MAN2B2	-0.3239741	0.7063514	1.0303255	1.0303255
KIF5B	ALS2	-0.8687759	0.16146761	1.03024351	1.03024351
UBC	ZWINT	-0.5954513	0.4346751	1.0301264	1.0301264
PALLD	ENG	0.38916737	-0.6409297	-1.03009707	1.03009707
DNMT1	RTN4	-0.6279068	0.40212762	1.03003442	1.03003442
EPS15	MPHOSPH6	-0.72660255	0.30341598	1.03001853	1.03001853
UBE2H	BMP1	0.40749657	-0.6225093	-1.03000587	1.03000587
DAZAP1	MTHFSD	0.2493642	-0.78062314	-1.02998734	1.02998734
ICK	CDK1	-0.7219814	0.3079972	1.0299786	1.0299786
KDR	TRIT1	-0.32605776	0.7039109	1.02996866	1.02996866
TRAF3IP1	MRPL17	-0.8489572	0.18100812	1.02996532	1.02996532
UBC	PNPT1	-0.3008409	0.7290713	1.0299122	1.0299122
INTS6	CRYZL1	0.47843194	-0.5513783	-1.02981024	1.02981024
PIAS2	ARHGEF12	-0.42594686	0.6037454	1.02969226	1.02969226
PTTG1	QSOX1	-0.39310318	0.63647926	1.02958244	1.02958244
ZNF608	NOL8	-0.18272212	0.8468528	1.02957492	1.02957492
UBC	RACGAP1	-0.46169287	0.56786346	1.02955633	1.02955633
COLGALT1	DPH6	0.66833854	-0.3611955	-1.02953404	1.02953404
TRIM23	MITF	-0.6136239	0.41576347	1.02938737	1.02938737
COPB1	AP3M1	-0.6126713	0.41671422	1.02938552	1.02938552
TRIAP1	TLN2	-0.48838192	0.5409843	1.02936622	1.02936622
ALDOB	HELLS	0.48257044	-0.5467494	-1.02931984	1.02931984
TRIM27	FAXDC2	-0.5034771	0.52584046	1.02931756	1.02931756
MRPL17	RNASE4	-0.83940625	0.18989287	1.02929912	1.02929912
UNC5C	RGS22	-0.4054801	0.62379605	1.02927615	1.02927615
PTPRN2	CEBPD	-0.46329662	0.56596243	1.02925905	1.02925905
TNFRSF25	CARD9	-0.6458339	0.383341	1.0291749	1.0291749
RNF150	NIPA2	-0.6643482	0.36482283	1.02917103	1.02917103
PRMT8	SNX18	-0.5463055	0.48281428	1.02911978	1.02911978
ADAM22	NDFIP2	-0.76740295	0.2616655	1.02906845	1.02906845
TCEB3	TMEM55A	-0.47537768	0.5536517	1.02902938	1.02902938
PFKP	SP3	-0.48757517	0.54140615	1.02898132	1.02898132
RRM1	LPIN1	-0.24263	0.7863321	1.0289621	1.0289621
NOD1	CASP2	0.37777826	-0.6510643	-1.02884256	1.02884256
ENG	SOX30	0.296265	-0.73232484	-1.02858984	1.02858984
UBC	CHTF8	-0.52843076	0.50010014	1.0285309	1.0285309
GAPDH	APOL2	0.49661225	-0.5319167	-1.02852895	1.02852895
DNAJB1	ATG2A	0.6785055	-0.35001776	-1.02852326	1.02852326
SNRPB2	EPS8	-0.44844052	0.58005977	1.02850029	1.02850029
COL5A1	C1QTNF6	-0.38592166	0.6423114	1.02823306	1.02823306
SSB	DCAF13	0.7445243	-0.2836994	-1.0282237	1.0282237
UBC	DNMT1	-0.32775608	0.70042276	1.02817884	1.02817884
APC	TMED5	-0.43034574	0.59760207	1.02794781	1.02794781
ARHGEF12	MRPS14	-0.7452	0.28270724	1.02790724	1.02790724
TRIM27	EGR1	0.5319095	-0.4959182	-1.0278277	1.0278277
AP3S2	ITGA8	0.7282328	-0.29944316	-1.02767596	1.02767596

SMYD3	EGR3	0.5972799	-0.43034786	-1.02762776	1.02762776
ITGB6	PDHB	-0.6707922	0.3568106	1.0276028	1.0276028
UBC	SPCS3	-0.4892903	0.53821254	1.02750284	1.02750284
HDAC1	HDAC11	-0.74961543	0.27786276	1.02747819	1.02747819
SSB	VLDLR	-0.5854291	0.44197744	1.02740654	1.02740654
FOXM1	DNAJC9	0.8464383	-0.18082832	-1.02726662	1.02726662
VAMP4	PRICKLE4	-0.4352695	0.5919224	1.0271919	1.0271919
SRGAP1	TEX264	-0.44430655	0.58288395	1.0271905	1.0271905
EFCAB14	PHF8	-0.29353324	0.73354805	1.02708129	1.02708129
SPCS3	PDZRN3	-0.70202744	0.32495576	1.0269832	1.0269832
RAB11A	OPTN	-0.6281144	0.39885807	1.02697247	1.02697247
UBC	ESRRG	-0.5287906	0.49817026	1.02696086	1.02696086
ZWINT	ADAMTS2	-0.4301445	0.59681475	1.02695925	1.02695925
METTL3	HIST1H2BE	-0.39994228	0.6269651	1.02690738	1.02690738
DCUN1D5	THRB	-0.2646342	0.7620404	1.0266746	1.0266746
ADAMTS2	SF3B3	-0.45045102	0.57615924	1.02661026	1.02661026
CHMP3	PDHB	-0.42775026	0.59885186	1.02660212	1.02660212
BMP15	SRGAP1	0.48213637	-0.5443701	-1.02650647	1.02650647
APBB2	ESRP2	-0.83698076	0.18950616	1.02648692	1.02648692
PELP1	NOL9	0.4768944	-0.54959106	-1.02648546	1.02648546
NDN	ZSCAN20	-0.42882723	0.5975785	1.02640573	1.02640573
AMOT	HSPA14	0.30245754	-0.7239107	-1.02636824	1.02636824
ASS1	TULP4	-0.3765541	0.6497265	1.0262806	1.0262806
ADAMTS9	CERS5	-0.29290265	0.73330355	1.0262062	1.0262062
DNAJC15	ARHGEF12	-0.60403	0.42188603	1.02591603	1.02591603
SERPINA1	CLSTN1	-0.25358465	0.7721847	1.02576935	1.02576935
MYL12A	GSTT2	0.41860285	-0.6071105	-1.02571335	1.02571335
SHC1	FOS	0.6320755	-0.39358795	-1.02566345	1.02566345
TRMT61A	ARHGEF12	0.6353267	-0.39033276	-1.02565946	1.02565946
NFATC2IP	GTF2I	-0.8375108	0.18807742	1.02558822	1.02558822
MAPKAPK3	SCML2	0.31488982	-0.71055484	-1.02544466	1.02544466
C10orf11	C11orf98	-0.5099717	0.5154145	1.0253862	1.0253862
NEGR1	METTL17	0.3706643	-0.65464675	-1.02531105	1.02531105
SOX4	C11orf63	0.526317	-0.49899322	-1.02531022	1.02531022
MFAP3	USP9X	-0.35276905	0.67248553	1.02525458	1.02525458
SORBS1	COL5A1	0.4843188	-0.54093426	-1.02525306	1.02525306
SLC37A4	SLC46A3	-0.6315035	0.39373267	1.02523617	1.02523617
PRMT8	FIP1L1	-0.62608415	0.3991349	1.02521905	1.02521905
TNFRSF21	MCTP1	-0.73910713	0.28611016	1.02521729	1.02521729
GEMIN8	SLC10A3	-0.3887627	0.6363357	1.0250984	1.0250984
FLNB	FANCG	-0.6194927	0.40556452	1.02505722	1.02505722
SIRPA	NPY1R	0.69340265	-0.33160493	-1.02500758	1.02500758
NUTF2	ELK1	0.6047881	-0.42019868	-1.02498678	1.02498678
DNAJC9	DNAJC21	-0.67156607	0.35332018	1.02488625	1.02488625
NDEL1	ARMC12	-0.6316308	0.39321792	1.02484872	1.02484872
EHBP1	COG6	-0.8083609	0.21630715	1.02466805	1.02466805
ZNF678	ZNF57	-0.6328187	0.39182907	1.02464777	1.02464777
RUSC1	HIST1H1D	-0.5983412	0.4262306	1.0245718	1.0245718
PDE4DIP	ERP44	-0.42722142	0.5973411	1.02456252	1.02456252
PRMT8	FBN1	-0.75142187	0.27307254	1.02449441	1.02449441
PIKFYVE	ACTN3	-0.4993772	0.5250647	1.0244419	1.0244419
PRR14L	DCUN1D3	-0.7945128	0.22991288	1.02442568	1.02442568
CETP	TYMP	-0.2518611	0.7725562	1.0244173	1.0244173
CCM2	SIRPA	0.6864418	-0.33794615	-1.02438795	1.02438795
DTL	DMXL2	-0.4491099	0.57510203	1.02421193	1.02421193

ALDOB	PCNA	0.49608478	-0.527948	-1.02403278	1.02403278
SYNPO	RTN4RL1	-0.59227926	0.43174714	1.0240264	1.0240264
LARP6	MST1R	0.2261858	-0.7977904	-1.0239762	1.0239762
KCNK5	EGR1	0.42119253	-0.6026887	-1.02388123	1.02388123
DCN	ZNF219	0.62513894	-0.39868784	-1.02382678	1.02382678
TRIT1	ABI2	0.5868255	-0.43696958	-1.02379508	1.02379508
RGS5	PCIF1	-0.55720353	0.46658245	1.02378598	1.02378598
ALAD	YME1L1	0.6822843	-0.34144855	-1.02373285	1.02373285
APRT	NFIA	-0.67783356	0.34561756	1.02345112	1.02345112
NAB2	RTN3	0.31388634	-0.709467	-1.02335334	1.02335334
PPIL2	STX6	-0.35221702	0.6710907	1.02330772	1.02330772
GDF5	FIGF	-0.26984397	0.7534625	1.02330647	1.02330647
SSRP1	PFDN4	0.4182255	-0.60507095	-1.02329645	1.02329645
ARHGEF37	FNBP4	0.41336522	-0.6099282	-1.02329342	1.02329342
UCK2	PNPT1	0.5816153	-0.44155154	-1.02316684	1.02316684
EPS8	HPS6	-0.69223696	0.33090472	1.02314168	1.02314168
PHPT1	NDUFC1	0.41712958	-0.60594803	-1.02307761	1.02307761
AP1G2	AAGAB	-0.4089178	0.614151	1.0230688	1.0230688
INO80D	ADAM19	0.5618741	-0.46114182	-1.02301592	1.02301592
PPIL2	TRIM21	-0.36593443	0.6570427	1.02297713	1.02297713
DPT	NAB2	0.28147966	-0.74148715	-1.02296681	1.02296681
IDS	EIF4G3	0.443661	-0.5793027	-1.0229637	1.0229637
CYR61	PLSCR3	0.62723523	-0.3956817	-1.02291693	1.02291693
KLF12	CCT3	0.21877795	-0.8040697	-1.02284765	1.02284765
C12orf49	PDE7A	0.45849186	-0.56422037	-1.02271223	1.02271223
DCN	CFLAR	0.7275438	-0.2951516	-1.0226954	1.0226954
INTS6	ZNF518B	0.5054756	-0.51721066	-1.02268626	1.02268626
PTGR2	CHD2	0.8484891	-0.17415233	-1.02264143	1.02264143
TARBP2	SOX4	-0.39476502	0.6278764	1.02264142	1.02264142
CSNK2A1	CLASP2	0.17319344	-0.8492957	-1.02248914	1.02248914
FGF18	FAM171A1	0.5571291	-0.46532574	-1.02245484	1.02245484
TBL1XR1	NFIB	0.6898865	-0.3325553	-1.0224418	1.0224418
POLK	RAB35	-0.58040804	0.4419827	1.02239074	1.02239074
WNK1	LOC10106017[illegible]	0.687173	-0.33516017	-1.02233317	1.02233317
COL4A1	VLDLR	-0.2917448	0.7305824	1.0223272	1.0223272
ADAMTS9	TLL2	0.5437577	-0.478506	-1.0222637	1.0222637
GLRB	ZDHHC14	0.26660615	-0.75563323	-1.02223938	1.02223938
SRSF1	MTHFSD	0.23373093	-0.7883633	-1.02209423	1.02209423
HIVEP3	LY75	0.54583085	-0.47623718	-1.02206803	1.02206803
CALR	LPCAT3	0.37176648	-0.65020335	-1.02196983	1.02196983
SNRPB2	DPP3	0.7793292	-0.2426178	-1.021947	1.021947
UTP18	HDAC11	-0.56325465	0.45862553	1.02188018	1.02188018
NVL	YME1L1	-0.39797315	0.62374735	1.0217205	1.0217205
TOP2A	DNAJC9	0.8227552	-0.19892383	-1.02167903	1.02167903
TAOK3	SLIRP	-0.38735926	0.63425756	1.02161682	1.02161682
GALNT11	PRELID1	-0.48817664	0.53319865	1.02137529	1.02137529
CRYZL1	IRF1	0.43683097	-0.5845212	-1.02135217	1.02135217
GDAP2	ZDHHC16	-0.3783108	0.64303756	1.02134836	1.02134836
INO80D	FAM104B	-0.46550158	0.5557695	1.02127108	1.02127108
TPO	CLPB	-0.47697285	0.5442968	1.02126965	1.02126965
GRWD1	WDR86	-0.50986075	0.5113806	1.02124135	1.02124135
SLC22A4	GFOD1	0.60649365	-0.4147337	-1.02122735	1.02122735
DKC1	CDKN3	0.7189434	-0.3022623	-1.0212057	1.0212057
PTPRN2	SLAMF1	-0.6077708	0.41324285	1.02101365	1.02101365
TBL1X	MYL9	-0.58657503	0.43443647	1.0210115	1.0210115

MCM6	LDLRAD4	-0.5184038	0.5024719	1.0208757	1.0208757
MCM8	SPIRE2	0.8246	-0.19624789	-1.02084789	1.02084789
ORC5	CCDC57	-0.52925044	0.49158594	1.02083638	1.02083638
RPS21	NUDCD2	0.37932038	-0.64150405	-1.02082443	1.02082443
SLC25A13	CHPF	-0.49241516	0.52837515	1.02079031	1.02079031
ZBTB44	KRT76	-0.46628693	0.5544887	1.02077563	1.02077563
CDCA7	PFDN4	0.4525375	-0.56822664	-1.02076414	1.02076414
IDH2	ACTN3	0.4062896	-0.61441994	-1.02070954	1.02070954
CCT3	SNX29	-0.4803862	0.5403145	1.0207007	1.0207007
NAB1	DHX30	-0.5271526	0.49345413	1.02060673	1.02060673
MYOC	OLFML2B	0.4769116	-0.54368937	-1.02060097	1.02060097
EFCAB14	ALDH1A1	0.59092927	-0.42965785	-1.02058712	1.02058712
CLEC3B	INPP5F	-0.46156004	0.5590027	1.02056274	1.02056274
ZHX3	PDIA6	-0.54771525	0.47275174	1.02046699	1.02046699
STX6	TLL2	0.43782914	-0.5825822	-1.02041134	1.02041134
ASB6	ASB14	0.27414504	-0.7462184	-1.02036344	1.02036344
ZWINT	PDE4DIP	-0.8203206	0.19987099	1.02019159	1.02019159
COL15A1	LCMT2	-0.5322731	0.48774	1.0200131	1.0200131
EPS15	ESRRG	-0.47418997	0.54574496	1.01993493	1.01993493
ZCCHC24	IRF1	0.39271316	-0.6271717	-1.01988486	1.01988486
NFIA	EMC6	-0.39338788	0.62637	1.01975788	1.01975788
PTPRG	TPBG	-0.41762957	0.6020361	1.01966567	1.01966567
SH3KBP1	PI3	-0.74509525	0.27453977	1.01963502	1.01963502
APC	AGPAT5	-0.46867555	0.5509405	1.01961605	1.01961605
SDC2	GSTM5	0.72626686	-0.29309803	-1.01936489	1.01936489
FGF18	CRYZL1	0.5957361	-0.423605	-1.0193411	1.0193411
UBE2H	PFDN4	-0.4290003	0.590329	1.0193293	1.0193293
ABCA1	PHF8	-0.15681529	0.8624868	1.01930209	1.01930209
COL1A1	BMPER	0.36320028	-0.65603197	-1.01923225	1.01923225
HIVEP3	ZNF467	0.50646406	-0.5126283	-1.01909236	1.01909236
CYTH2	PHLDB2	0.54781055	-0.4710974	-1.01890795	1.01890795
AGPAT5	SLC43A2	0.5513128	-0.46757987	-1.01889267	1.01889267
RAB21	RAB30	0.8183601	-0.20050614	-1.01886624	1.01886624
PARP9	NTNG2	0.2547608	-0.7639992	-1.01876	1.01876
STAC	POLDIP3	0.32839075	-0.6903522	-1.01874295	1.01874295
TUFM	ELK1	0.6273179	-0.39128417	-1.01860207	1.01860207
PRMT1	CBX3	-0.19390346	0.82464266	1.01854612	1.01854612
RBM17	OLA1	-0.48164925	0.5366872	1.01833645	1.01833645
SERPINA1	SERPINA3	0.6813133	-0.33701327	-1.01832657	1.01832657
RSPO3	ZC3H3	0.52901226	-0.48925084	-1.0182631	1.0182631
CD69	SPIN3	-0.42560267	0.5926242	1.01822687	1.01822687
NFIA	BST2	-0.63429135	0.38390917	1.01820052	1.01820052
PDE4DIP	FGFR1OP2	-0.6609982	0.35716468	1.01816288	1.01816288
GNAL	MTURN	-0.526709	0.49145225	1.01816125	1.01816125
PAPPA	SIKE1	0.40446433	-0.61369157	-1.0181559	1.0181559
CEACAM1	PCDH9	-0.30821407	0.709896	1.01811007	1.01811007
CAP2	TBRG4	-0.5453762	0.47270718	1.01808338	1.01808338
IL18BP	MZF1	-0.4698849	0.5481761	1.018061	1.018061
SOX4	MXRA7	0.28715548	-0.7309028	-1.01805828	1.01805828
OLMALINC	SUSD1	-0.35087055	0.66718245	1.018053	1.018053
IDS	SRD5A1	-0.57768935	0.44015127	1.01784062	1.01784062
MAP4	CDK5	-0.43759292	0.5802	1.01779292	1.01779292
CAST	MFAP3	-0.35101223	0.6667126	1.01772483	1.01772483
FUT11	IRF4	-0.7126118	0.30494672	1.01755852	1.01755852
SEPT11	ARPP21	-0.45455053	0.56297314	1.01752367	1.01752367

COL15A1	EPB41L4A	0.40781572	-0.60970277	-1.01751849	1.01751849
DPP3	PHF5A	0.84020734	-0.17728823	-1.01749557	1.01749557
SDPR	ELP6	-0.70581025	0.31167537	1.01748562	1.01748562
AGPAT5	ASB1	-0.25459918	0.7628178	1.01741698	1.01741698
ARPP21	AIMP1	-0.71708494	0.30029	1.01737494	1.01737494
STAT1	MCM5	-0.47085145	0.54647344	1.01732489	1.01732489
ATG2A	JUND	0.6876541	-0.32965648	-1.01731058	1.01731058
FGFR1OP	ARHGEF37	-0.7152923	0.30197272	1.01726502	1.01726502
ANKRD28	ANKRD35	0.555438	-0.4617809	-1.0172189	1.0172189
MRPS14	PDHB	-0.1122128	0.9049839	1.0171967	1.0171967
XRCC5	CSRP2BP	-0.4900771	0.5271104	1.0171875	1.0171875
YWHAG	CCND2	-0.5877914	0.42935923	1.01715063	1.01715063
GEMIN4	RRBP1	-0.59273326	0.42439115	1.01712441	1.01712441
PLCB4	NUDCD2	-0.6758708	0.3412433	1.0171141	1.0171141
RBPJ	RGS3	-0.6044895	0.41260615	1.01709565	1.01709565
ARPP21	PPP1R1B	0.5226021	-0.49444985	-1.01705195	1.01705195
IGF1R	ITGA8	0.55609614	-0.4609524	-1.01704854	1.01704854
GAPDH	BIK	0.6658838	-0.3511205	-1.0170043	1.0170043
MITF	SOX4	0.26411024	-0.75275457	-1.01686481	1.01686481
NOL8	SPEF2	0.13370235	-0.88316005	-1.0168624	1.0168624
FOXN3	FGF18	0.4097972	-0.606977	-1.0167742	1.0167742
RAB5A	ZNF101	0.49935406	-0.5172664	-1.01662046	1.01662046
ENG	BCAR1	0.5847919	-0.4317761	-1.016568	1.016568
STK10	ADPGK	-0.21074618	0.8058027	1.01654888	1.01654888
NOL9	PNPLA4	-0.6630562	0.3534824	1.0165386	1.0165386
DNAJB1	DNAJB2	0.74878323	-0.26775053	-1.01653376	1.01653376
TRMT61A	NPM1	0.5847137	-0.43176496	-1.01647866	1.01647866
BUB1B	SEH1L	0.69831747	-0.31802306	-1.01634053	1.01634053
CTNNA1	NEGR1	0.26825455	-0.7480327	-1.01628725	1.01628725
EZH2	KIF11	0.9297178	-0.0864649	-1.0161827	1.0161827
ZBTB20	STX6	-0.71395874	0.30187958	1.01583832	1.01583832
SLU7	IFT81	0.57286364	-0.4429489	-1.01581254	1.01581254
EIF5	NAALADL2	-0.43546405	0.5802863	1.01575035	1.01575035
PTTG1	KIF11	0.9471319	-0.06858051	-1.01571241	1.01571241
ITGB5	ITGA8	0.7221714	-0.2935384	-1.0157098	1.0157098
PFKP	PLEKHH1	-0.3470002	0.66859305	1.01559325	1.01559325
PPM1A	PSMB3	-0.62532	0.39023435	1.01555435	1.01555435
SCARB2	RPL14	-0.22240694	0.7931292	1.01553614	1.01553614
TRIAP1	S100A16	0.7716256	-0.24383022	-1.01545582	1.01545582
AUP1	STAT3	-0.3896823	0.6257452	1.0154275	1.0154275
LDLRAD4	C3orf17	-0.27362216	0.74169827	1.01532043	1.01532043
HNRNPC	PFDN4	0.50954646	-0.5057586	-1.01530506	1.01530506
TPO	VEGFA	-0.6319347	0.3833653	1.0153	1.0153
MFAP3	SREK1IP1	-0.26243332	0.75278187	1.01521519	1.01521519
GRM5	NEXN	-0.6389045	0.3762686	1.0151731	1.0151731
UBE2B	MAP4	0.56445485	-0.45064545	-1.0151003	1.0151003
ZMYND11	CERS5	0.65670764	-0.3583908	-1.01509844	1.01509844
CSNK2A1	PGRMC2	-0.34943253	0.6656352	1.01506773	1.01506773
MMD	COL6A3	0.72003794	-0.29494998	-1.01498792	1.01498792
UNC5C	ZHX1	-0.38391554	0.6310707	1.01498624	1.01498624
DPT	LTBP4	0.7509451	-0.26400438	-1.01494948	1.01494948
DIEXF	AP3S2	-0.49131978	0.5236195	1.01493928	1.01493928
GART	ZBTB10	-0.35772038	0.6571158	1.01483618	1.01483618
COL1A1	EFEMP2	0.7572437	-0.25757506	-1.01481876	1.01481876
NEDD4L	IL18	0.5598418	-0.4549476	-1.0147894	1.0147894

RSU1	RBM45	-0.50191414	0.51271206	1.0146262	1.0146262
KLF12	DIEXF	0.21240428	-0.8021883	-1.01459258	1.01459258
TBL1X	RGS3	-0.27797696	0.7365691	1.01454606	1.01454606
ZNF426	EFEMP2	0.70492	-0.30955058	-1.01447058	1.01447058
SERBP1	GTF2I	-0.6768561	0.33753642	1.01439252	1.01439252
PCDH17	PRUNE2	-0.38235447	0.6319389	1.01429337	1.01429337
RNF141	PRICKLE1	0.5657229	-0.44844952	-1.01417242	1.01417242
THRB	CPXM1	0.6972968	-0.31681237	-1.01410917	1.01410917
CADM1	EIF4E3	-0.351293	0.66273415	1.01402715	1.01402715
TXNL1	ZNF148	-0.32929957	0.6847198	1.01401937	1.01401937
UBC	CDCA7	-0.6005413	0.41344166	1.01398296	1.01398296
MBNL1	ZDHHC14	0.39781263	-0.61598516	-1.01379779	1.01379779
ADPGK	STIM2	-0.26837116	0.74537337	1.01374453	1.01374453
GTF3C2	WDR13	-0.44955206	0.5641723	1.01372436	1.01372436
HDAC11	RACGAP1	-0.74822325	0.26549965	1.0137229	1.0137229
KIAA0101	SCML2	0.48726758	-0.5264521	-1.01371968	1.01371968
FADS2	SAMD4A	-0.78100336	0.232625	1.01362836	1.01362836
ESRRG	DNAJB2	-0.7207885	0.29283985	1.01362835	1.01362835
SEPT11	TGM2	0.36117458	-0.6524439	-1.01361848	1.01361848
SYNPO2	WDR27	0.71380424	-0.29962647	-1.01343071	1.01343071
MGAT5	SPEF2	-0.38521558	0.6282024	1.01341798	1.01341798
STX8	PI3	-0.7003583	0.31301668	1.01337498	1.01337498
ESRRG	TM9SF3	-0.6734337	0.33994097	1.01337467	1.01337467
SF3B3	TM4SF18	-0.5906618	0.4226918	1.0133536	1.0133536
LDLRAD4	GALNT2	-0.514632	0.4986503	1.0132823	1.0132823
EZH2	ZBTB10	-0.61711895	0.3960668	1.01318575	1.01318575
ZWINT	IDS	-0.74559355	0.26758662	1.01318017	1.01318017
HSD17B6	LOX	0.5539628	-0.45918292	-1.01314572	1.01314572
WARS	LCA5	-0.52122456	0.4918225	1.01304706	1.01304706
KSR1	DDR2	0.32826757	-0.68470216	-1.01296973	1.01296973
XRCC5	MMD	-0.7595924	0.25331447	1.01290687	1.01290687
APC	EIF2B2	-0.53113896	0.4817596	1.01289856	1.01289856
SRPRB	PCED1B	-0.5745016	0.4383527	1.0128543	1.0128543
YARS	KLF12	0.33122638	-0.68156374	-1.01279012	1.01279012
SP3	IKZF1	-0.6090331	0.40366527	1.01269837	1.01269837
INIP	POLR2H	0.35994872	-0.65258366	-1.01253238	1.01253238
RPN2	AUP1	-0.26980424	0.7427143	1.01251854	1.01251854
ITCH	NUDT21	-0.4098869	0.6026237	1.0125106	1.0125106
XPO1	TBC1D1	-0.47593957	0.536549	1.01248857	1.01248857
XRCC5	RNF150	-0.7663775	0.24609463	1.01247213	1.01247213
FGFR1OP	ADAMTS2	-0.24287716	0.7695445	1.01242166	1.01242166
ARPP19	ZNF804A	-0.5362439	0.47613883	1.01238273	1.01238273
CEACAM1	MRPS14	-0.3461353	0.6662169	1.0123522	1.0123522
SMC4	CDKN3	0.6344135	-0.37780628	-1.01221978	1.01221978
EPS15	LAP3	-0.29534665	0.7168355	1.01218215	1.01218215
XRCC5	EPHA1	0.39036962	-0.6217799	-1.01214952	1.01214952
GNAQ	MRPS22	-0.42248216	0.5896349	1.01211706	1.01211706
COL27A1	TMEM44	-0.29941037	0.71270514	1.01211551	1.01211551
COL1A1	LAMA1	-0.49565515	0.5162164	1.01187155	1.01187155
SPIRE1	CNPY2	-0.6239854	0.38781157	1.01179697	1.01179697
BUB1B	STK36	-0.4585298	0.5530535	1.0115833	1.0115833
SIRPA	ATP6V0E2	0.67627406	-0.33519983	-1.01147389	1.01147389
MFAP3	CLK2	-0.38286507	0.6285899	1.01145497	1.01145497
FOXN3	COL6A3	0.64071393	-0.37057492	-1.01128885	1.01128885
SIKE1	ADAMTS9	0.32159525	-0.6896513	-1.01124655	1.01124655

USP4	TYRO3	-0.4974518	0.5137369	1.0111887	1.0111887
KCNAB1	SOX4	0.37379915	-0.6373416	-1.01114075	1.01114075
CBX4	CHCHD7	0.3418739	-0.6692452	-1.0111191	1.0111191
SAP30L	TM2D2	-0.25857148	0.75254625	1.01111773	1.01111773
LDLRAD4	CCT3	-0.5140807	0.49695292	1.01103362	1.01103362
ZNF142	ZNF704	0.3135289	-0.69737774	-1.01090664	1.01090664
ANKRD28	NUP62	-0.5595331	0.45136604	1.01089914	1.01089914
DYSF	STK36	-0.44145682	0.5694143	1.01087112	1.01087112
CDCA7	DCAF7	-0.6054801	0.40531844	1.01079854	1.01079854
EMC6	ADM	-0.22301282	0.7877839	1.01079672	1.01079672
GGCT	OGFR	0.7303114	-0.28046572	-1.01077712	1.01077712
PCCB	CEACAM1	0.53249294	-0.4782593	-1.01075224	1.01075224
VCAM1	ZNF397	0.270888	-0.7398598	-1.0107478	1.0107478
ANKRD28	NUDCD2	-0.53264374	0.47800788	1.01065162	1.01065162
NEFH	SLC24A4	-0.38160095	0.6289831	1.01058405	1.01058405
ITGA8	C6orf106	0.6392115	-0.37132594	-1.01053744	1.01053744
SERPINA1	MRPL47	0.70047975	-0.3100381	-1.01051785	1.01051785
EPS15	AMMECR1	0.46777925	-0.5427329	-1.01051215	1.01051215
STAT3	CLSTN1	-0.20614776	0.80432075	1.01046851	1.01046851
SERBP1	ADGRA2	-0.7642088	0.24621964	1.01042844	1.01042844
KLHL34	C11orf31	0.42322686	-0.5871063	-1.01033316	1.01033316
PPM1A	C10orf54	0.542703	-0.46755177	-1.01025477	1.01025477
ANPEP	SCRN1	0.4413265	-0.56890965	-1.01023615	1.01023615
OXR1	LCMT2	-0.4562652	0.55396676	1.01023196	1.01023196
LBP	DPAGT1	0.81884664	-0.1913326	-1.01017924	1.01017924
DUSP3	CADM1	-0.4576887	0.5524847	1.0101734	1.0101734
KAT6B	LOC10099669:	-0.3607878	0.6493609	1.0101487	1.0101487
PIK3CA	PSMA4	-0.5868149	0.42331916	1.01013406	1.01013406
CAST	SCML2	-0.8043349	0.20574313	1.01007803	1.01007803
MARK1	HPS6	-0.50879985	0.50127786	1.01007771	1.01007771
NFIA	NIPA2	-0.49248338	0.51759255	1.01007593	1.01007593
ALS2	RAB11A	-0.50009054	0.509985	1.01007554	1.01007554
PDE5A	WARS	-0.32110018	0.6889469	1.01004708	1.01004708
CTNNA1	CPXM1	-0.37139064	0.63864774	1.01003838	1.01003838
NIPA2	RGS22	-0.5015964	0.50833696	1.00993336	1.00993336
SNRPB2	LMO3	-0.7394573	0.2701803	1.0096376	1.0096376
NFKB2	MST1R	-0.45549497	0.55406046	1.00955543	1.00955543
KIF5B	IPO9	0.39395198	-0.61545825	-1.00941023	1.00941023
GALNS	SAFB2	-0.54471445	0.46466997	1.00938442	1.00938442
SSBP2	SLC41A2	-0.53732353	0.47201544	1.00933897	1.00933897
MRPS14	AGPAT5	-0.46083724	0.5484789	1.00931614	1.00931614
HIST1H2AD	PHF8	0.6892322	-0.32004553	-1.00927773	1.00927773
ARHGEF12	FAM149A	-0.72355026	0.28562987	1.00918013	1.00918013
TMED3	PPP1R1B	-0.5874202	0.42175734	1.00917754	1.00917754
PI3	PGM2	0.30540434	-0.70357835	-1.00898269	1.00898269
ADSL	ARHGEF37	-0.5732755	0.43569013	1.00896563	1.00896563
EIF4G3	KCMF1	0.648673	-0.36029136	-1.00896436	1.00896436
KIRREL	LRP8	-0.7324652	0.27639434	1.00885954	1.00885954
IP6K2	GPT	0.66114163	-0.3475086	-1.00865023	1.00865023
WDR59	SLIRP	0.32823607	-0.68037677	-1.00861284	1.00861284
ZFR	EFHD1	-0.43768242	0.5709007	1.00858312	1.00858312
LOC145783	MLC1	0.45112735	-0.5573535	-1.00848085	1.00848085
MTHFR	PHF8	-0.3458197	0.66263026	1.00844996	1.00844996
TMEM163	RMDN1	0.67805666	-0.3302696	-1.00832626	1.00832626
EXD2	FAM200B	0.38388127	-0.6244269	-1.00830817	1.00830817

RPRD1A	CADM1	-0.2997755	0.70847136	1.00824686	1.00824686
TULP4	ASB6	-0.46187454	0.5463465	1.00822104	1.00822104
CEPT1	CSK	-0.55428535	0.45389777	1.00818312	1.00818312
ARPC5L	CWF19L2	-0.74136597	0.2667359	1.00810187	1.00810187
COPB1	TEC	-0.38034344	0.62769663	1.00804007	1.00804007
TAF1C	RIC1	-0.36899817	0.6390366	1.00803477	1.00803477
PID1	ARHGEF12	-0.84892863	0.15905389	1.00798252	1.00798252
ITCH	BID	-0.6429665	0.36498407	1.00795057	1.00795057
SSRP1	NAP1L1	-0.51146024	0.49648282	1.00794306	1.00794306
UBE2H	CBX3	0.40497738	-0.6029431	-1.00792048	1.00792048
SERPINA1	CTNNA1	-0.40989202	0.59800124	1.00789326	1.00789326
ESRRG	TMED8	-0.50137216	0.50648504	1.0078572	1.0078572
CCT3	TM9SF3	-0.40454987	0.6031287	1.00767857	1.00767857
KDR	LAMA1	-0.41454324	0.5931262	1.00766944	1.00766944
QKI	MED31	-0.43937474	0.5682688	1.00764354	1.00764354
ZHX1	BID	-0.53336537	0.47427258	1.00763795	1.00763795
COMMD3	TAOK3	-0.4208585	0.5867488	1.0076073	1.0076073
SNTB2	DNAH17	-0.5922858	0.41531157	1.00759737	1.00759737
ZDHHC14	KCMF1	0.48284453	-0.5246896	-1.00753413	1.00753413
SEH1L	WDTC1	-0.8567451	0.15078372	1.00752882	1.00752882
FAM204A	DCUN1D3	0.4952592	-0.51222396	-1.00748316	1.00748316
H2AFY	TRMT13	-0.39642677	0.6109813	1.00740807	1.00740807
RBMS3	RPL27A	-0.75034046	0.2569855	1.00732596	1.00732596
APOE	ALAD	0.70917946	-0.29813874	-1.0073182	1.0073182
BID	NOVA1	-0.6905979	0.3166668	1.0072647	1.0072647
TSPAN9	CWF19L2	-0.49933827	0.5079248	1.00726307	1.00726307
CHD2	MFAP3	-0.70265603	0.30459595	1.00725198	1.00725198
INIP	FGFR1OP2	0.37000334	-0.63704133	-1.00704467	1.00704467
RNF150	MYH6	-0.73966897	0.26733038	1.00699935	1.00699935
DPYSL3	UNC5C	0.43559274	-0.5712768	-1.00686954	1.00686954
SIX1	CADM1	-0.28597906	0.720879	1.00685806	1.00685806
KIAA0141	ZSCAN20	-0.44035012	0.5664092	1.00675932	1.00675932
RMI1	RRAS	-0.7891701	0.217509	1.0066791	1.0066791
KIAA0513	NXPH2	-0.34679607	0.6598593	1.00665537	1.00665537
AMMECR1	ATP6V1E2	-0.2558377	0.7507725	1.0066102	1.0066102
FAM43A	ARMC12	-0.41666132	0.58994085	1.00660217	1.00660217
KCNRG	LONRF2	-0.6177186	0.38886985	1.00658845	1.00658845
ZWINT	ZHX3	-0.71350884	0.29305294	1.00656178	1.00656178
EFCAB14	FAM57A	-0.54524565	0.46129116	1.00653681	1.00653681
RGS5	NELFCD	-0.6403953	0.36605856	1.00645386	1.00645386
KCNK15	SFN	0.7852983	-0.22104558	-1.00634388	1.00634388
MGAT5	EIF4G3	-0.4979053	0.508369	1.0062743	1.0062743
PCOLCE2	BMP1	0.4446724	-0.561531	-1.0062034	1.0062034
GNAQ	DNAJC9	-0.62906575	0.37697747	1.00604322	1.00604322
NKIRAS1	SKA3	-0.7141835	0.29184645	1.00602995	1.00602995
ZDHHC14	HIBADH	0.50618905	-0.4997048	-1.00589385	1.00589385
GSTT1	CIT	-0.5345158	0.47136754	1.00588334	1.00588334
CSNK2A1	SPIRE1	-0.44442415	0.5614517	1.00587585	1.00587585
SORL1	WWC1	0.552252	-0.4535627	-1.0058147	1.0058147
LTBP4	HDAC1	-0.501744	0.5040171	1.0057611	1.0057611
KLF12	MFSD12	0.61167175	-0.39408228	-1.00575403	1.00575403
UBC	DIEXF	-0.36890203	0.63682777	1.0057298	1.0057298
B2M	ASB9	-0.5523001	0.4534073	1.0057074	1.0057074
SRSF1	RGS22	-0.6002394	0.40536937	1.00560877	1.00560877
MYOC	ATP5SL	-0.65629005	0.34931338	1.00560343	1.00560343

UNC5C	KPNA4	-0.4359001	0.5696885	1.0055886	1.0055886
PELP1	MCM5	0.696459	-0.30912778	-1.00558678	1.00558678
IDS	TXNL1	-0.25389835	0.7516758	1.00557415	1.00557415
KLF12	XPO5	0.2296844	-0.7757916	-1.005476	1.005476
CADM1	RHOQ	-0.29809403	0.70735776	1.00545179	1.00545179
SGCB	PRICKLE1	0.5916426	-0.41379273	-1.00543533	1.00543533
CLPB	PDZD2	-0.3321616	0.6732215	1.0053831	1.0053831
PELI2	MED31	-0.4737594	0.53161705	1.00537645	1.00537645
IL21	TYW5	-0.46344778	0.5418733	1.00532108	1.00532108
TLN2	GALNT2	-0.5867796	0.41852158	1.00530118	1.00530118
WTAP	SPEF2	0.34073594	-0.6644853	-1.00522124	1.00522124
NASP	TRANK1	-0.27986506	0.7252715	1.00513656	1.00513656
EIF6	NOP16	0.83045703	-0.17461118	-1.00506821	1.00506821
XPO1	RELA	-0.7321249	0.27289397	1.00501887	1.00501887
HSPH1	KIRREL	-0.66973156	0.33522448	1.00495604	1.00495604
CADM1	PRKRIR	-0.4563288	0.54857373	1.00490253	1.00490253
NMT2	QSOX1	0.3857022	-0.61898696	-1.00468916	1.00468916
LPCAT1	ZNF467	-0.53657573	0.46809462	1.00467035	1.00467035
MAPK13	APP	-0.30505916	0.69958514	1.0046443	1.0046443
CAB39L	CASP5	-0.76819736	0.236425	1.00462236	1.00462236
QKI	PPA1	-0.44588286	0.5585816	1.00446446	1.00446446
BUB1B	BIK	0.8427829	-0.16162047	-1.00440337	1.00440337
CSNK2A1	CALM1	-0.3026342	0.7016945	1.0043287	1.0043287
MRPS12	SPERT	0.33298618	-0.67124724	-1.00423342	1.00423342
KATNAL1	ADAMTS9	-0.47115308	0.5330459	1.00419898	1.00419898
GMPS	CWF19L2	-0.82000023	0.18414102	1.00414125	1.00414125
THRB	SNRPC	-0.6543087	0.34979656	1.00410526	1.00410526
TYRO3	TNIP1	-0.2267862	0.77730703	1.00409323	1.00409323
NOL9	CHD2	-0.43881056	0.565223	1.00403356	1.00403356
BTC	OLFML2B	0.5394886	-0.4644721	-1.0039607	1.0039607
PSMB2	NOP16	0.9449945	-0.05887985	-1.00387435	1.00387435
FAM107B	LOC10272456	0.40801626	-0.5958223	-1.00383856	1.00383856
ADAM10	ABI2	0.55411416	-0.44971368	-1.00382784	1.00382784
GALNT2	HIST1H2BE	0.66363394	-0.34017998	-1.00381392	1.00381392
GALNT2	EHBP1	-0.6425963	0.361155	1.0037513	1.0037513
DPAGT1	ZNF546	-0.3790972	0.62461877	1.00371597	1.00371597
NXF1	MAPK13	-0.30400467	0.69970286	1.00370753	1.00370753
SGCB	NAB2	0.5535122	-0.45001146	-1.00352366	1.00352366
DNAJB1	POU2F2	-0.77632385	0.22719909	1.00352294	1.00352294
OPRK1	C5AR1	-0.47325298	0.53015894	1.00341192	1.00341192
FAM136A	ESRP2	0.62986195	-0.3735094	-1.00337135	1.00337135
IL2	ZNF385D	0.65541464	-0.3478667	-1.00328134	1.00328134
MED15	PPIL2	-0.3942871	0.6088987	1.0031858	1.0031858
MITF	SIRPA	0.514674	-0.4884952	-1.0031692	1.0031692
STXBP6	CPXM1	0.48550695	-0.51752	-1.00302695	1.00302695
MAP4K4	CFAP58	0.5051219	-0.4978924	-1.0030143	1.0030143
TRPM3	PINK1	-0.6972014	0.30581206	1.00301346	1.00301346
ZDHHC14	ITIH5	0.2998306	-0.70308095	-1.00291155	1.00291155
SSB	STXBP6	-0.70656884	0.29627374	1.00284258	1.00284258
UBC	DNAJC9	-0.43428937	0.568463	1.00275237	1.00275237
UROS	SLIRP	0.6573464	-0.34536618	-1.00271258	1.00271258
TOP2A	MFAP3	0.26016608	-0.74246377	-1.00262985	1.00262985
SAA4	SDPR	-0.35062215	0.65193844	1.00256059	1.00256059
PPA1	PCDH9	-0.3388889	0.66367066	1.00255956	1.00255956
GTF3C1	IL18BP	-0.2982767	0.70426214	1.00253884	1.00253884

CADM1	KLHL17	0.3258665	-0.67667055	-1.00253705	1.00253705
KDR	C1QTNF6	-0.40240288	0.60012156	1.00252444	1.00252444
GNAQ	SOX4	0.4536742	-0.5487453	-1.0024195	1.0024195
NAB2	SKA3	-0.7885728	0.21379156	1.00236436	1.00236436
WBP4	ARHGEF12	-0.8427173	0.15959619	1.00231349	1.00231349
WDR46	CHD2	-0.4220999	0.58014524	1.00224514	1.00224514
EFEMP2	THBS3	0.9021697	-0.10006675	-1.00223645	1.00223645
VAV2	MOCOS	0.44394985	-0.5582598	-1.00220965	1.00220965
PTPN21	QSOX1	0.6074309	-0.39475298	-1.00218388	1.00218388
SERP1	HAMP	0.65540236	-0.34672472	-1.00212708	1.00212708
HDAC11	SNRPB2	-0.51637167	0.48575175	1.00212342	1.00212342
KLF12	KIAA1715	0.4699251	-0.5320963	-1.0020214	1.0020214
PALLD	LTBP4	0.3567859	-0.6452331	-1.002019	1.002019
PRPH	BCAT2	-0.20384574	0.798098	1.00194374	1.00194374
LYRM1	C1QBP	-0.54577774	0.45612642	1.00190416	1.00190416
BRF1	ANXA11	0.36556292	-0.6363202	-1.00188312	1.00188312
PALMD	SAMD4A	-0.66530675	0.33652568	1.00183243	1.00183243
DLG1	CADM1	-0.4899295	0.51186776	1.00179726	1.00179726
DNAJC15	KIF13A	0.815106	-0.18657461	-1.00168061	1.00168061
TACC1	GALNT2	-0.39487213	0.6067073	1.00157943	1.00157943
FBN1	THBS3	0.67843425	-0.3230952	-1.00152945	1.00152945
NAB2	AIDA	0.6567966	-0.34470737	-1.00150397	1.00150397
NTRK2	SLC24A4	0.4623444	-0.53914595	-1.00149035	1.00149035
ORC5	ABI2	0.6011006	-0.40033844	-1.00143904	1.00143904
KPNA4	RNF150	-0.14041068	0.86098087	1.00139155	1.00139155
MFAP3	GSTM4	-0.20168181	0.79970014	1.00138195	1.00138195
RAB11A	LHPP	0.48424459	-0.5171241	-1.00136869	1.00136869
DCUN1D5	PCED1B	-0.5107049	0.49064955	1.00135445	1.00135445
BUB1B	FOXM1	0.95445454	-0.04679063	-1.001245166	1.001245166
COPZ1	PDZRN3	-0.5937849	0.40744135	1.00122625	1.00122625
DCAF8	DTL	-0.75220406	0.24901359	1.00121765	1.00121765
SLC25A13	CAPS2	-0.24522358	0.75598305	1.00120663	1.00120663
KIAA0101	RIMS2	0.5761811	-0.42500842	-1.00118952	1.00118952
NAP1L1	CYP46A1	0.6960566	-0.30506384	-1.00112044	1.00112044
ZFAND5	GPR155	-0.4250454	0.57603616	1.00108156	1.00108156
SAP18	NEGR1	-0.40275404	0.59820163	1.00095567	1.00095567
PTTG1	CDC37L1	-0.77453375	0.22637203	1.00090578	1.00090578
LPGAT1	TRIM21	0.33849138	-0.6623357	-1.00082708	1.00082708
TCAIM	DNAH10	0.37313133	-0.62767416	-1.00080549	1.00080549
SERBP1	INIP	0.5518931	-0.44884282	-1.00073592	1.00073592
NFIA	CBLN2	0.38815138	-0.6125572	-1.00070858	1.00070858
GRM5	CHD1L	0.29991952	-0.7007433	-1.00066282	1.00066282
SRSF1	LNX1	-0.84343237	0.15722166	1.00065403	1.00065403
PCNA	USP9X	-0.36636275	0.6342602	1.00062295	1.00062295
LTF	SLPI	0.43467492	-0.565942	-1.00061692	1.00061692
SOBP	LIME1	-0.5359923	0.46459347	1.00058577	1.00058577
GAPDH	NFE2L1	-0.85725415	0.14315648	1.00041063	1.00041063
YARS	AUP1	-0.22810902	0.7722622	1.00037122	1.00037122
XRCC5	NGFR	0.88656735	-0.11379779	-1.00036514	1.00036514
SERPINA1	EPHX2	-0.70197135	0.29831648	1.00028783	1.00028783
PPA1	LYSMD2	0.742158	-0.25811982	-1.00027782	1.00027782
NOL10	TEX264	-0.37767497	0.622566	1.00024097	1.00024097
BACE2	PRUNE2	-0.541043	0.45919323	1.00023623	1.00023623
TCEB3	URI1	0.6499754	-0.35017595	-1.00015135	1.00015135
CALD1	HLA-G	0.637929	-0.36221766	-1.00014666	1.00014666

MED17	TSPAN9	0.35238475	-0.6477422	-1.00012695	1.00012695
PGF	C8orf4	0.67974925	-0.32031363	-1.00006288	1.00006288
PPM1A	BMP1	0.41559735	-0.584458	-1.00005535	1.00005535
DLGAP1	FLNB	-0.36030015	0.6397461	1.00004625	1.00004625
ESRRG	COPS7B	0.6647972	-0.33521503	-1.00001223	1.00001223
