# Supplementary material for: Identification of key regulatory genes connected to NF-κB family of proteins in visceral adipose tissues using gene expression and weighted protein interaction network
Source: PLoS One. 2019 Apr 23;14(4):e0214337. doi: 10.1371/journal.pone.0214337 (PMC6478283; doi:10.1371/journal.pone.0214337)
Supplement: S6 Table — (PDF) [file pone.0214337.s006.pdf]

S6 Table: Sgo annotations based on DPcc in normal vs Obese

Gene-1	Gene-2	Normal	Obese	D_{PCC} (ABS)	S_{GO}
MCM3	TOP2A	0.7113794	-0.7908391	1.5022185	1
XRCC5	RBM17	-0.6855559	0.765678	1.4512339	1
TEX10	PELP1	0.7758664	-0.6404441	1.4163105	1
NEDD4L	PRICKLE1	0.7761754	-0.6375003	1.4136757	1
MCM3	HLA-C	-0.60021347	0.81271887	1.41293234	1
COL4A1	FZR1	0.7556257	-0.6345547	1.3901804	1
TOP2A	BIK	0.8647102	-0.52093965	1.38564985	1
ESRRG	RPRD1A	-0.6117029	0.7674302	1.3791331	1
RAB35	COG6	0.640026	-0.7240482	1.3640742	1
HDAC1	RBM17	-0.6978421	0.6587166	1.3565587	1
MCM3	ZBTB10	-0.60310096	0.7494847	1.35258566	1
LMO3	SOX4	0.6061915	-0.7387266	1.3449181	1
MCM3	RBM17	-0.6941539	0.64797425	1.34212815	1
NEGR1	MSRB3	-0.8506514	0.4910484	1.3416998	1
CSTF3	NEGR1	0.63993984	-0.6943373	1.33427714	1
QKI	C1orf131	-0.58601725	0.74562836	1.33164561	1
PCNA	ZBTB10	-0.6408693	0.67680013	1.31766943	1
GALNT2	ADGRA2	-0.5844702	0.7323404	1.3168106	1
SNRPB2	RBM17	-0.53370345	0.76769567	1.30139912	1
ANKRD28	LCLAT1	-0.5706625	0.71929157	1.28995407	1
MCM3	THRB	-0.71150345	0.5740923	1.28559575	1
TRIAP1	SH3RF1	-0.48181644	0.80352235	1.28533879	1
ZBTB10	HDAC1	-0.6360605	0.64366376	1.27972426	1
RBM17	SLIRP	-0.5577912	0.7207314	1.2785226	1
MOB3B	SNRPC	-0.49947715	0.7718912	1.27136835	1
SH3D19	PRICKLE1	0.588061	-0.68275344	1.27081444	1
PCDH17	TOP3A	-0.60044414	0.668883	1.26932714	1
KPNB1	DCAF7	-0.4774065	0.7871937	1.2646002	1
ITGB6	MYOF	0.6040414	-0.658583	1.2626244	1
RAE1	DCAF8	-0.418044	0.8405329	1.2585769	1
RAB27A	LAGE3	0.5993815	-0.65711904	1.25650054	1
RAB11A	RAB40B	-0.6919919	0.56070274	1.25269464	1
TCEB3	TCF3	0.5512225	-0.69814813	1.24937063	1
NGFR	TIMM23	0.6069994	-0.63517964	1.24217904	1
CSNK2A1	CD48	0.6353478	-0.6065509	1.2418987	1
TBL1X	DCAF8	-0.5542635	0.6867638	1.2410273	1
CYR61	WISP1	0.6109724	-0.6254056	1.236378	1
CCT3	DCAF7	-0.5380505	0.69643104	1.23448154	1
RBM17	CSNK2A1	-0.44787282	0.7865706	1.23444342	1
NEGR1	PHF8	0.6168795	-0.6148601	1.2317396	1
FANCI	FANCB	0.6176202	-0.6111999	1.2288201	1
ITGB6	TNC	0.66467714	-0.5583196	1.22299674	1
MED15	RACGAP1	-0.6363869	0.5859455	1.2223324	1
PRICKLE1	CD164	0.7149994	-0.50563186	1.22063126	1
RUFY3	ZWILCH	-0.7970466	0.42337856	1.22042516	1
FAM96A	RBM17	-0.7590593	0.45921287	1.21827217	1
ANKRD28	ZWILCH	-0.6711186	0.54566497	1.21678357	1
MCM3	PTTG1	0.8143693	-0.400565	1.2149343	1
UBC	MCM3	-0.5083812	0.7026548	1.211036	1
DKC1	RBM17	-0.56339586	0.6475767	1.21097256	1
SLC7A1	INIP	0.54728913	-0.6533572	1.20064633	1
RMI1	SESN1	-0.43570518	0.76488334	1.20058852	1
CSF3R	BMP1	-0.58471745	0.61303264	1.19775009	1

SNCG	MCM3	0.4127377	-0.78263354	1.19537124	1
MCM3	SPAG5	0.73816586	-0.4560622	1.19422806	1
ADGRA2	MMAB	-0.39438456	0.7996576	1.19404216	1
XRCC5	MOB3B	-0.54961085	0.6441632	1.19377405	1
MCM4	MCM3	0.86193496	-0.33182037	1.19375533	1
HSP90AA1	C1QTNF6	0.51867145	-0.6738759	1.19254735	1
PALLD	NEGR1	-0.5843992	0.6051836	1.1895828	1
GAPDH	RBM17	-0.79458725	0.3927499	1.18733715	1
TEC	PTPN21	0.62572354	-0.5575818	1.18330534	1
TRIAP1	PCED1B	-0.65734273	0.5259372	1.18327993	1
AGTRAP	ENTHD2	-0.3687012	0.81358516	1.18228636	1
TRIO	ARHGEF37	-0.75964236	0.42085308	1.18049544	1
MAPK10	PRICKLE1	0.78494626	-0.39296487	1.17791113	1
TRAF3IP1	GALNT2	-0.56073445	0.61346555	1.1742	1
XPO1	AMMECR1	0.42881215	-0.74465036	1.17346251	1
DCAF8	SF3B3	-0.72542244	0.44709456	1.172517	1
MCM3	CHD2	-0.6058033	0.5646926	1.1704959	1
ADA	ENTHD2	-0.65718454	0.51197	1.16915454	1
FANCA	CDK1	0.7449918	-0.42201433	1.16700613	1
EFCAB14	GET4	-0.37335375	0.79350424	1.16685799	1
USP4	ZNF276	-0.7989136	0.3675607	1.1664743	1
ACVR2A	WDR27	0.73194844	-0.43315393	1.16510237	1
CAB39L	YME1L1	0.5409567	-0.623102	1.1640587	1
MCM5	MCM4	0.73123837	-0.43095213	1.1621905	1
FAM96A	TSPAN9	0.5425861	-0.6191251	1.1617112	1
GEMIN4	ZNF202	-0.4898532	0.6701468	1.16	1
MCM3	OPTN	-0.511448	0.648262	1.15971	1
ARNTL	C12orf49	-0.43920115	0.7172291	1.15643025	1
MPPED2	TIMELESS	-0.4121234	0.74092376	1.15304716	1
APOE	RMI1	-0.66333586	0.4862168	1.14955266	1
SRSF1	RBM17	-0.55379677	0.59519684	1.14899361	1
NEGR1	HELLS	0.58985794	-0.55873114	1.14858908	1
MCM3	HLA-B	-0.35771003	0.78977287	1.1474829	1
ITGB5	PDLIM4	0.46718037	-0.6751796	1.14235997	1
PARK2	PTPN4	-0.5985422	0.54295677	1.14149897	1
SNRPB2	SNTB1	-0.6307162	0.508367	1.1390832	1
CTNNA1	TRO	-0.65859157	0.47834104	1.13693261	1
PDE9A	LAGE3	-0.7517738	0.38443324	1.13620704	1
ZBTB10	PNPT1	-0.48682344	0.6491506	1.13597404	1
RUFY3	RMI1	-0.80906034	0.3246371	1.13369744	1
COMTD1	METTL3	-0.7782576	0.3549531	1.1332107	1
CCT6A	INIP	0.6225192	-0.5089921	1.1315113	1
CSRNP1	ADAMTSL3	-0.53188175	0.5983693	1.13025105	1
SH3RF1	PRICKLE1	0.7206998	-0.4083675	1.1290673	1
COMMD3	ALS2	-0.65942615	0.46762642	1.12705257	1
CDK6	NEGR1	-0.53276855	0.5922269	1.12499545	1
MCM6	ADGRA2	-0.6794518	0.44438645	1.12383825	1
NUB1	PER3	-0.568129	0.55496514	1.12309414	1
GSTP1	RMI1	-0.46952736	0.65353674	1.1230641	1
XPO1	ZWILCH	-0.3493971	0.7736436	1.1230407	1
TRIM25	DCAF8	-0.2875623	0.83428013	1.12184243	1
MAP4K4	MYL12A	0.23769654	-0.88372785	1.12142439	1
LARP6	TRO	0.40539902	-0.7143764	1.11977542	1
XRCC5	CD48	0.71854156	-0.39971268	1.11825424	1
KATNAL1	NELFCD	-0.5515645	0.56514525	1.11670975	1
NEGR1	NDFIP2	0.85021955	-0.26427695	1.1144965	1

KANK1	PRICKLE1	0.52613926	-0.5873488	1.11348806	1
IP6K2	NEGR1	0.5524746	-0.559207	1.1116816	1
NEGR1	PNPT1	0.58121806	-0.5300154	1.11123346	1
ESRRG	PID1	-0.69109595	0.41924796	1.11034391	1
IGF2R	LAGE3	-0.62079984	0.48883292	1.10963276	1
VPS33B	MLST8	-0.4795739	0.62919044	1.10876434	1
EIF4G3	PTPN21	0.5074395	-0.5996893	1.1071288	1
MCM3	CD48	0.6075954	-0.49880737	1.10640277	1
COG6	ANTXR2	-0.4602705	0.6427911	1.1030616	1
RBM17	GSTM5	0.8908159	-0.2110684	1.1018843	1
RBM17	DAZAP1	-0.5438388	0.55200905	1.09584785	1
UBC	ASB6	-0.5738544	0.5210189	1.0948733	1
COMMD3	VAPB	-0.39712018	0.6956961	1.09281628	1
RSU1	ISYNA1	0.3043943	-0.78771	1.0921043	1
APC	COMMD3	-0.47250283	0.61860675	1.09110958	1
IFNAR2	HELLS	-0.4181368	0.67231685	1.09045365	1
TCEB3	LCA5	-0.33578095	0.7536885	1.08946945	1
TBL1XR1	DCAF11	0.729263	-0.3602027	1.0894657	1
SLC7A1	CCDC117	0.5806442	-0.5078374	1.0884816	1
RBPJ	CCM2	-0.5958817	0.49147847	1.08736017	1
TOP2A	MCM5	0.7187027	-0.36840472	1.08710742	1
GTF3C1	ARPIN	0.49155882	-0.5941319	1.08569072	1
MAD2L1BP	CBLB	-0.47125065	0.6129404	1.08419105	1
ZBTB10	NOL9	-0.5248361	0.5585209	1.083357	1
RUFY3	VANGL1	-0.38680127	0.6961557	1.08295697	1
AGPAT5	LOC100996693	0.36950636	-0.71333957	1.08284593	1
BIK	BCL2	-0.7581294	0.32391524	1.08204464	1
TACC1	KIAA0391	-0.6769218	0.4049558	1.0818776	1
PCBP2	LNX1	0.30501166	-0.7758244	1.08083606	1
MPPED2	GSTM3	0.46799046	-0.61190784	1.0798983	1
NFKBIE	MIB2	0.6234857	-0.4561796	1.0796653	1
CEACAM1	CD164	-0.41444218	0.66521865	1.07966083	1
ARPP21	ETV3	-0.78889364	0.28922898	1.07812262	1
C12orf49	EGR1	0.46467733	-0.6133107	1.07798803	1
GSS	PARPBP	0.66556865	-0.41179305	1.0773617	1
SERBP1	ZBTB10	-0.6078574	0.4693091	1.0771665	1
HTT	FAM111A	-0.56689245	0.51018673	1.07707918	1
TCEB3	SNX1	-0.43235403	0.6431276	1.07548163	1
ZWINT	FANCB	0.72250295	-0.35273087	1.07523382	1
PPIL2	NEGR1	0.18870448	-0.8856535	1.07435798	1
SORBS1	PRICKLE1	0.39259452	-0.68136865	1.07396317	1
ZWINT	DCAF7	-0.59859043	0.4750465	1.07363693	1
POSTN	HELLS	-0.23205052	0.84146	1.07351052	1
GSS	DCAF8	-0.23436886	0.83849144	1.0728603	1
IFNAR2	DFNB31	-0.5066317	0.5661708	1.0728025	1
AGPAT5	CCDC117	0.3875129	-0.6846923	1.0722052	1
NEGR1	AGTRAP	0.33158484	-0.7400453	1.07163014	1
DCAF8	HPS6	-0.5539695	0.5162357	1.0702052	1
COG6	CLASRP	0.58974123	-0.4802644	1.07000563	1
CWF19L2	SDPR	0.5655303	-0.5035309	1.0690612	1
PSMB3	NAALADL2	-0.66128206	0.40726128	1.06854334	1
RBM45	MTHFSD	0.39024082	-0.6750295	1.06527032	1
RUFY3	MPHOSPH6	-0.71511245	0.34836802	1.06348047	1
LMO3	COL1A1	0.16715042	-0.8956476	1.06279802	1
BCS1L	SPERT	0.5736686	-0.48694137	1.06060997	1
ZWINT	GDAP2	-0.4487793	0.61167043	1.06044973	1

DTL	DCAF7	-0.4971641	0.56317335	1.06033745	1
SUFU	CA8	0.3113392	-0.7487991	1.0601383	1
LTA	MPHOSPH6	0.50067705	-0.5594571	1.06013415	1
COL5A1	AGTRAP	-0.31809118	0.741767	1.05985818	1
TYRO3	PRICKLE1	0.34964362	-0.7101503	1.05979392	1
DYSF	ENTHD2	-0.5185474	0.54112077	1.05966817	1
PCNA	CCDC90B	-0.28364736	0.7757734	1.05942076	1
TOR1AIP1	CWF19L2	0.46786803	-0.5910845	1.05895253	1
CSF3R	POLN	0.77685916	-0.28087813	1.05773729	1
SH3RF1	TBRG4	-0.42614567	0.6262359	1.05238157	1
BCL2	PRICKLE1	0.2665354	-0.78372824	1.05026364	1
CCT3	ADGRA2	-0.538114	0.5119274	1.0500414	1
MCM6	PCED1B	-0.6652462	0.38476378	1.05000998	1
TBC1D9B	PRICKLE1	0.47176006	-0.5776979	1.04945796	1
WARS	CD48	0.49212626	-0.5558259	1.04795216	1
CEACAM1	CCDC90B	-0.72654366	0.32124338	1.04778704	1
KCND2	TIMM23	-0.4389272	0.60861665	1.04754385	1
KARS	NAALADL2	-0.4804996	0.5668296	1.0473292	1
FOXJ2	TMEM159	-0.39228848	0.6550155	1.04730398	1
GFOD1	STAT3	0.39264286	-0.65258837	1.04523123	1
KIRREL	AAGAB	-0.47012866	0.5748902	1.04501886	1
ARPP21	KLHL5	-0.7031152	0.3417744	1.0448896	1
GNA12	CCDC117	0.53229994	-0.51215833	1.04445827	1
UBC	NFATC2IP	-0.7758697	0.26826587	1.04413557	1
RBM17	SOX4	0.5145349	-0.5285694	1.0431043	1
NAALADL2	SNRPC	-0.70690274	0.3349186	1.04182134	1
GYS1	MCM3	-0.5137019	0.5278243	1.0415262	1
POC1A	WRAP53	0.5938008	-0.44731456	1.04111536	1
ZFP36L1	WDR27	-0.31012595	0.7307512	1.04087715	1
UBC	ZWILCH	-0.45063546	0.5895613	1.04019676	1
NXF1	AMMECR1	0.39274746	-0.6473658	1.04011326	1
MAP4	PRICKLE1	0.4514179	-0.5884929	1.0399108	1
ATG2A	EGR1	0.49253404	-0.5471933	1.03972734	1
TEX264	C8orf33	-0.3618476	0.6774701	1.0393177	1
NEGR1	RNF207	0.3447052	-0.69423765	1.03894285	1
GEM	ADGRA2	0.84813964	-0.19013816	1.0382778	1
CIAO1	DCAF8	-0.49166837	0.54513663	1.036805	1
CCT3	NAALADL2	-0.5647555	0.47177148	1.03652698	1
CCDC90B	TBC1D1	-0.42032644	0.6154796	1.03580604	1
TSPAN9	CNOT11	0.60545003	-0.42890885	1.03435888	1
ANKRD28	TEAD4	-0.51011187	0.52319604	1.03330791	1
MCM3	CBX3	-0.28231075	0.7509167	1.03322745	1
APC	C19orf25	-0.35791075	0.6749284	1.03283915	1
TCF3	SEH1L	0.6875104	-0.34528542	1.03279582	1
LMO3	KCNQ4	-0.3001947	0.7321482	1.0323429	1
ABI2	SREK1IP1	0.5666971	-0.46564087	1.03233797	1
UBC	C1orf131	-0.31514254	0.7171173	1.03225984	1
NFASC	MAGOHB	-0.653534	0.37844336	1.03197736	1
EPS15	MPHOSPH6	-0.72660255	0.30341598	1.03001853	1
DAZAP1	MTHFSD	0.2493642	-0.78062314	1.02998734	1
COPB1	AP3M1	-0.6126713	0.41671422	1.02938552	1
ALDOB	HELLS	0.48257044	-0.5467494	1.02931984	1
DNAJB1	ATG2A	0.6785055	-0.35001776	1.02852326	1
COL5A1	C1QTNF6	-0.38592166	0.6423114	1.02823306	1
ITGB6	PDHB	-0.6707922	0.3568106	1.0276028	1
SRGAP1	TEX264	-0.44430655	0.58288395	1.0271905	1

EFCAB14	PHF8	-0.29353324	0.73354805	1.02708129	1
NEGR1	METTL17	0.3706643	-0.65464675	1.02531105	1
NAB2	RTN3	0.31388634	-0.709467	1.02335334	1
AP1G2	AAGAB	-0.4089178	0.614151	1.0230688	1
SRSF1	MTHFSD	0.23373093	-0.7883633	1.02209423	1
GDAP2	ZDHHC16	-0.3783108	0.64303756	1.02134836	1
SLC22A4	GFOD1	0.60649365	-0.4147337	1.02122735	1
ORC5	CCDC57	-0.52925044	0.49158594	1.02083638	1
RAB21	RAB30	0.8183601	-0.20050614	1.01886624	1
STAC	POLDIP3	0.32839075	-0.6903522	1.01874295	1
RBM17	OLA1	-0.48164925	0.5366872	1.01833645	1
STAT1	MCM5	-0.47085145	0.54647344	1.01732489	1
ATG2A	JUND	0.6876541	-0.32965648	1.01731058	1
GAPDH	BIK	0.6658838	-0.3511205	1.0170043	1
RAB5A	ZNF101	0.49935406	-0.5172664	1.01662046	1
BUB1B	SEH1L	0.69831747	-0.31802306	1.01634053	1
CTNNA1	NEGR1	0.26825455	-0.7480327	1.01628725	1
EIF5	NAALADL2	-0.43546405	0.5802863	1.01575035	1
RSU1	RBM45	-0.50191414	0.51271206	1.0146262	1
SYNPO2	WDR27	0.71380424	-0.29962647	1.01343071	1
EZH2	ZBTB10	-0.61711895	0.3960668	1.01318575	1
WARS	LCA5	-0.52122456	0.4918225	1.01304706	1
ANKRD28	NUP62	-0.5595331	0.45136604	1.01089914	1
VCAM1	ZNF397	0.270888	-0.7398598	1.0107478	1
ANKRD28	NUDCD2	-0.53264374	0.47800788	1.01065162	1
EPS15	AMMECR1	0.46777925	-0.5427329	1.01051215	1
SERBP1	ADGRA2	-0.7642088	0.24621964	1.01042844	1
KAT6B	LOC100996693	-0.3607878	0.6493609	1.0101487	1
SNRPB2	LMO3	-0.7394573	0.2701803	1.0096376	1
WDR59	SLIRP	0.32823607	-0.68037677	1.00861284	1
RPRD1A	CADM1	-0.2997755	0.70847136	1.00824686	1
ARPC5L	CWF19L2	-0.74136597	0.2667359	1.00810187	1
COPB1	TEC	-0.38034344	0.62769663	1.00804007	1
PID1	ARHGEF12	-0.84892863	0.15905389	1.00798252	1
COMMD3	TAOK3	-0.4208585	0.5867488	1.0076073	1
SEH1L	WDTC1	-0.8567451	0.15078372	1.00752882	1
INIP	FGFR1OP2	0.37000334	-0.63704133	1.00704467	1
RMI1	RRAS	-0.7891701	0.217509	1.0066791	1
AMMECR1	ATP6V1E2	-0.2558377	0.7507725	1.0066102	1
EFCAB14	FAM57A	-0.54524565	0.46129116	1.00653681	1
B2M	ASB9	-0.5523001	0.4534073	1.0057074	1
PELP1	MCM5	0.696459	-0.30912778	1.00558678	1
BUB1B	BIK	0.8427829	-0.16162047	1.00440337	1
MRPS12	SPERT	0.33298618	-0.67124724	1.00423342	1
MED15	PPIL2	-0.3942871	0.6088987	1.0031858	1
MAP4K4	CFAP58	0.5051219	-0.4978924	1.0030143	1
KDR	C1QTNF6	-0.40240288	0.60012156	1.00252444	1
NAB2	SKA3	-0.7885728	0.21379156	1.00236436	1
WDR46	CHD2	-0.4220999	0.58014524	1.00224514	1
TACC1	GALNT2	-0.39487213	0.6067073	1.00157943	1
DCUN1D5	PCED1B	-0.5107049	0.49064955	1.00135445	1
DCAF8	DTL	-0.75220406	0.24901359	1.00121765	1
ZFAND5	GPR155	-0.4250454	0.57603616	1.00108156	1
SAP18	NEGR1	-0.40275404	0.59820163	1.00095567	1
SERBP1	INIP	0.5518931	-0.44884282	1.00073592	1
SRSF1	LNX1	-0.84343237	0.15722166	1.00065403	1

TCEB3	URI1	0.6499754	-0.35017595	1.00015135	1
ESRRG	COPS7B	0.6647972	-0.33521503	1.00001223	1
CSNK1G2	CDK10	-0.22675765	0.8322353	1.05899295	0.964
QKI	DIEXF	-0.55084234	0.58976936	1.1406117	0.944
CDC7	CDK10	-0.71748704	0.61478335	1.33227039	0.915
SRSF1	NXF1	-0.57557195	0.5786942	1.15426615	0.912
ZNF202	MZF1	-0.3873601	0.66122246	1.04858256	0.909
P4HA1	PFDN4	0.8007077	-0.79464555	1.59535325	0.908
NAP1L1	SERBP1	-0.44640467	0.7054672	1.15187187	0.901
SSRP1	NAP1L1	-0.51146024	0.49648282	1.00794306	0.896
DNAJB1	DNAJB2	0.74878323	-0.26775053	1.01653376	0.884
SSB	LARP6	-0.30586734	0.78756064	1.09342798	0.871
ZWINT	PDE4DIP	-0.8203206	0.19987099	1.02019159	0.869
COL1A1	SDC2	0.40228355	-0.6409002	1.04318375	0.867
RAE1	NXF1	-0.3099789	0.84992075	1.15989965	0.866
HNRNPL	SNRPD3	0.34499562	-0.70912737	1.05412299	0.861
KANK1	STX6	-0.5675339	0.50640213	1.07393603	0.856
HNRNPA1	PRRC2A	-0.5642691	0.49814156	1.06241066	0.856
FHOD1	EXOSC8	-0.6887996	0.67712444	1.36592404	0.851
HSPA1L	HSPA1A	-0.7127358	0.7443941	1.4571299	0.845
ZBTB7A	ZBTB24	0.51536334	-0.5750412	1.09040454	0.843
RBCK1	KCNRG	0.65722334	-0.45961252	1.11683586	0.838
COL3A1	LAMA1	-0.65799004	0.62341124	1.28140128	0.837
ESRRG	FOXN3	-0.5227986	0.5083067	1.0311053	0.831
XPO1	IPO11	-0.6258964	0.54343957	1.16933597	0.826
CDK6	CDK10	0.37102935	-0.79431385	1.1653432	0.825
CTGF	WISP1	0.68132305	-0.5482444	1.22956745	0.818
MRPL17	POC1A	0.8826445	-0.52615	1.4087945	0.815
CADM1	KIAA1715	-0.68141055	0.63299024	1.31440079	0.815
TNFAIP2	HGF	0.71663713	-0.5712255	1.28786263	0.815
AIDA	C10orf54	0.66780835	-0.60333085	1.2711392	0.815
NFIA	AMMECR1	0.66065186	-0.600358	1.26100986	0.815
LOC100996693	DNAJC9	0.52931434	-0.7259336	1.25524794	0.815
LZTS1	TNFAIP2	-0.52054703	0.7116318	1.23217883	0.815
ATP6V1G1	C10orf54	-0.61456835	0.60099983	1.21556818	0.815
EMC6	HOXA7	-0.6081232	0.59016865	1.19829185	0.815
RSPO3	MZF1	0.62120795	-0.5679554	1.18916335	0.815
ALDH4A1	DFNB31	0.4930366	-0.6560589	1.1490955	0.815
ASB1	DNAJC9	-0.3834596	0.7550266	1.1384862	0.815
FTL	RSPO3	-0.6523495	0.4827626	1.1351121	0.815
ABCG1	TEX10	-0.74455565	0.37405452	1.11861017	0.815
POLR2D	NEGR1	0.6551444	-0.4520929	1.1072373	0.815
HDAC11	MCM3	-0.80089515	0.30178115	1.1026763	0.815
CSRP2BP	DNAJC9	-0.50650805	0.5806158	1.08712385	0.815
GALNT2	KIAA1715	-0.35888886	0.7242846	1.08317346	0.815
KCNRG	DNAJC9	0.57508415	-0.5019004	1.07698455	0.815
ALAD	PRICKLE1	0.50911564	-0.56226206	1.0713777	0.815
SCARB2	DNAJC9	-0.7732841	0.28791463	1.06119873	0.815
SOD2	IL32	0.606067	-0.45107317	1.05714017	0.815
MRPL17	CAB39L	-0.8894879	0.14118452	1.03067242	0.815
TRAF3IP1	MRPL17	-0.8489572	0.18100812	1.02996532	0.815
FOXM1	DNAJC9	0.8464383	-0.18082832	1.02726662	0.815
DNAJC9	DNAJC21	-0.67156607	0.35332018	1.02488625	0.815
TOP2A	DNAJC9	0.8227552	-0.19892383	1.02167903	0.815
NFIA	EMC6	-0.39338788	0.62637	1.01975788	0.815
EMC6	ADM	-0.22301282	0.7877839	1.01079672	0.815

PPM1A	C10orf54	0.542703	-0.46755177	1.01025477	0.815
GNAQ	DNAJC9	-0.62906575	0.37697747	1.00604322	0.815
UBC	DNAJC9	-0.43428937	0.568463	1.00275237	0.815
KLF12	KIAA1715	0.4699251	-0.5320963	1.0020214	0.815
STAT3	JUN	0.5783673	-0.5161511	1.0945184	0.813
MITF	SOX4	0.26411024	-0.75275457	1.01686481	0.813
SDC2	GSTM5	0.72626686	-0.29309803	1.01936489	0.812
TEAD4	FOS	0.6803981	-0.43715927	1.11755737	0.81
SP3	IKZF1	-0.6090331	0.40366527	1.01269837	0.81
STAT3	ARNTL	-0.43507928	0.82089096	1.25597024	0.806
USP4	USP9X	-0.60097766	0.49917448	1.10015214	0.804
NUDCD2	HLA-C	-0.7280905	0.48678458	1.21487508	0.802
CCT3	PFDN4	0.41293752	-0.7455363	1.15847382	0.802
STAT3	FOS	0.7200754	-0.7234661	1.4435415	0.797
PDE4DIP	DCUN1D5	-0.32123178	0.72346514	1.04469692	0.796
PFDN4	EXOSC8	0.5361857	-0.6293977	1.1655834	0.792
UBC	DIEXF	-0.36890203	0.63682777	1.0057298	0.792
LCMT2	PCMTD1	-0.5500099	0.79282504	1.34283494	0.791
PLOD3	P4HTM	-0.4091011	0.7145684	1.1236695	0.791
BUB1B	STK36	-0.4585298	0.5530535	1.0115833	0.791
ICK	CDK1	-0.7219814	0.3079972	1.0299786	0.79
PGF	C8orf4	0.67974925	-0.32031363	1.00006288	0.789
NOL8	ESRP2	0.58806103	-0.5204917	1.10855273	0.788
FLNB	FANCG	-0.6194927	0.40556452	1.02505722	0.788
CCT6A	PFDN4	0.5090917	-0.63450265	1.14359435	0.786
TAOK3	MAPK13	-0.55625266	0.48827466	1.04452732	0.786
CBX3	NOP56	-0.47170082	0.570689	1.04238982	0.786
UBC	TBRG4	-0.6679893	0.40027618	1.06826548	0.782
PDE4DIP	SLIRP	-0.5743951	0.7516256	1.3260207	0.779
KIAA0101	COL1A1	-0.52437705	0.5913987	1.11577575	0.779
STAT3	JUNB	0.62601656	-0.5454114	1.17142796	0.772
GSTM1	GSTM5	0.8144388	-0.32727993	1.14171873	0.771
CASP6	MALT1	-0.6552626	0.4567788	1.1120414	0.769
NDFIP2	HSPB8	-0.5175481	0.5286341	1.0461822	0.769
DLGAP1	FLNB	-0.36030015	0.6397461	1.00004625	0.768
PAX2	KLF12	-0.28146842	0.7843493	1.06581772	0.766
KIRREL	NOL8	-0.37321374	0.79609835	1.16931209	0.764
EPS15	CCT3	-0.65270823	0.48376718	1.13647541	0.763
PPIL2	TRIM21	-0.36593443	0.6570427	1.02297713	0.761
CAP2	TBRG4	-0.5453762	0.47270718	1.01808338	0.76
ZHX2	ZNF202	-0.4670785	0.8016713	1.2687498	0.759
SP3	ZNF704	0.42617965	-0.68144774	1.10762739	0.759
WRAP53	CHD2	-0.73855925	0.33959028	1.07814953	0.759
SSB	DCAF13	0.7445243	-0.2836994	1.0282237	0.759
GTF2I	SOX4	0.71291053	-0.47237834	1.18528887	0.758
RUFY3	FANCI	-0.72921365	0.4386647	1.16787835	0.758
AIDA	VPS16	0.6559119	-0.48493427	1.14084617	0.758
PDE4DIP	MOCOS	-0.67457616	0.41170415	1.08628031	0.758
KIAA0101	RIMS2	0.5761811	-0.42500842	1.00118952	0.757
SIRT7	PELP1	0.31591538	-0.8010819	1.11699728	0.754
CTNNA1	LRRC16A	-0.58339894	0.5230261	1.10642504	0.754
COL1A1	LAMA1	-0.49565515	0.5162164	1.01187155	0.753
ABI2	HTATSF1	0.43066204	-0.68601036	1.1166724	0.75
GTF3C2	MTHFSD	0.2629797	-0.84749097	1.11047067	0.75
CCT3	SYCP2	-0.7417758	0.36332184	1.10509764	0.75
PFDN4	PPP1R13L	0.55293137	-0.5298478	1.08277917	0.75

ARNTL	RRBP1	-0.52673286	0.5294475	1.05618036	0.75
KLF12	TIAL1	0.12928444	-0.9057491	1.03503354	0.75
ZCCHC24	IRF1	0.39271316	-0.6271717	1.01988486	0.75
WWTR1	AMOT	-0.59752774	0.63270485	1.23023259	0.749
CALD1	HLA-G	0.637929	-0.36221766	1.00014666	0.749
PPIB	HNRNPL	0.5447583	-0.50198734	1.04674564	0.748
KSR1	DDR2	0.32826757	-0.68470216	1.01296973	0.745
ESRRG	KANK1	-0.4917046	0.77668875	1.26839335	0.744
SERPINA1	CTNNA1	-0.40989202	0.59800124	1.00789326	0.744
TNC	LOX	0.66996866	-0.45230088	1.12226954	0.743
COL1A1	EFEMP2	0.7572437	-0.25757506	1.01481876	0.743
ESRRG	PDE4DIP	-0.63184583	0.65505725	1.28690308	0.739
TRAP1	APP	-0.42484367	0.6357621	1.06060577	0.739
DCUN1D5	HLA-C	-0.44687223	0.5940498	1.04092203	0.738
PDE4DIP	ERP44	-0.42722142	0.5973411	1.02456252	0.738
ITGB1	COL1A1	0.3337619	-0.7299039	1.0636658	0.735
MYOM1	ARPC5L	-0.67755723	0.36501363	1.04257086	0.735
ESRRG	PVR	0.7541373	-0.3593888	1.1135261	0.734
UBC	MRPL9	-0.4282755	0.61211324	1.04038874	0.733
AIMP1	PFDN4	0.6637982	-0.37550443	1.03930263	0.733
SSRP1	PFDN4	0.4182255	-0.60507095	1.02329645	0.733
PDE4DIP	FGFR1OP2	-0.6609982	0.35716468	1.01816288	0.732
QKI	PNPT1	-0.6164963	0.55719644	1.17369274	0.728
CAP2	NAB2	0.55788994	-0.595908	1.15379794	0.728
IGF2R	CALD1	0.60491306	-0.43061996	1.03553302	0.728
DDX6	DDX54	-0.61319894	0.57970726	1.1929062	0.727
ESRRG	NFIA	-0.7137432	0.36423326	1.07797646	0.727
MCM6	CBX3	-0.23152962	0.8249951	1.05652472	0.727
NAP1L1	BID	-0.6549522	0.39993054	1.05488274	0.726
POSTN	NUDCD2	-0.4658458	0.6939619	1.1598077	0.725
BID	BCL2L1	0.6780408	-0.51420134	1.19224214	0.723
ESRRG	PTTG1	0.58200264	-0.5458637	1.12786634	0.723
CEACAM1	SSFA2	-0.3781209	0.6864549	1.0645758	0.723
CSRP2BP	GET4	-0.6641821	0.4188429	1.083025	0.722
EFEMP2	NID2	0.61518186	-0.56723356	1.18241542	0.721
SCARB2	GIPC1	0.50431967	-0.6062013	1.11052097	0.721
LAMA1	CNTNAP1	-0.3957782	0.7160822	1.1118604	0.72
UBC	TIMELESS	-0.38052148	0.6916902	1.07221168	0.719
MAP1B	RPL14	-0.59971946	0.46552166	1.06524112	0.719
ABI2	NOL8	0.79753774	-0.38143715	1.17897489	0.718
GALNT11	GALNT2	-0.7279422	0.33548272	1.06342492	0.717
EPS15	ESRRG	-0.47418997	0.54574496	1.01993493	0.716
YWHAG	CCND2	-0.5877914	0.42935923	1.01715063	0.715
ESRRG	PARVA	-0.6211608	0.67109597	1.29225677	0.714
SORBS1	COL5A1	0.4843188	-0.54093426	1.02525306	0.714
HSD17B1	HSD11B1	-0.5437215	0.59811795	1.14183945	0.712
TXNIP	SMAD7	-0.48638016	0.55969864	1.0460788	0.712
QKI	CNOT11	-0.55218977	0.73432446	1.28651423	0.711
EPS15	PSMB3	-0.70325464	0.5374246	1.24067924	0.711
CD47	CTNNA1	-0.8061776	0.62431586	1.43049346	0.708
ADAM22	NDFIP2	-0.76740295	0.2616655	1.02906845	0.706
SHQ1	COL5A1	-0.36673638	0.70004374	1.06678012	0.705
GDF5	FIGF	-0.26984397	0.7534625	1.02330647	0.705
CAST	RBM45	-0.34418926	0.8180549	1.16224416	0.704
COL5A1	SDC2	0.59049535	-0.4870071	1.07750245	0.703
UBC	DHX30	-0.39483097	0.67189497	1.06672594	0.703

CRYZL1	TP53I3	0.6626646	-0.6270307	1.2896953	0.701
CALD1	GABBR1	-0.45572054	0.5961504	1.05187094	0.7
KMT2E	RTP4	0.62693214	-0.40790692	1.03483906	0.7
PCNA	TOP2A	0.816714	-0.48899704	1.30571104	0.699
AGPAT5	CSPG5	-0.40926233	0.7721068	1.18136913	0.699
ARHGEF2	RCC2	-0.88251185	0.43579084	1.31830269	0.698
PCNA	TEFM	-0.5488623	0.48797062	1.03683292	0.698
SFN	SERBP1	0.71890205	-0.46149254	1.18039459	0.696
USP9X	MALT1	-0.80719745	0.35338166	1.16057911	0.696
RARA	ARNTL	-0.6977274	0.445814	1.1435414	0.696
MYOC	INTS6	0.5125604	-0.5876353	1.1001957	0.696
APBB2	TOR1AIP1	0.7359421	-0.35006818	1.08601028	0.696
CUX1	CTNNA1	-0.80673116	0.5041051	1.31083626	0.695
UBC	STX6	-0.47045258	0.7437598	1.21421238	0.694
CALD1	COL3A1	0.7369608	-0.42747268	1.16443348	0.694
EPS8	HPS6	-0.69223696	0.33090472	1.02314168	0.694
SERPINA1	CLSTN1	-0.25358465	0.7721847	1.02576935	0.692
STK10	ADPGK	-0.21074618	0.8058027	1.01654888	0.692
CD69	SPIN3	-0.42560267	0.5926242	1.01822687	0.691
PSMB2	SPG20	-0.7259946	0.8019689	1.5279635	0.69
LDLRAD4	CNOT11	-0.6005252	0.7011989	1.3017241	0.69
EFCAB14	EFHD2	-0.5642069	0.6961921	1.260399	0.69
LDLRAD4	TIMELESS	-0.6573043	0.5827976	1.2401019	0.69
NAALADL2	MSRB1	-0.56262267	0.6737894	1.23641207	0.69
LDLRAD4	UBE2B	0.8111707	-0.39975074	1.21092144	0.69
NEGR1	SEMA4B	0.5695496	-0.6230023	1.1925519	0.69
ESRRG	LDLRAD4	-0.5257557	0.65377116	1.17952686	0.69
LDLRAD4	GET4	-0.57808375	0.5731146	1.15119835	0.69
ZBTB10	MSRB1	-0.69867396	0.43466866	1.13334262	0.69
ZNF106	MYL12A	-0.667963	0.45492303	1.12288603	0.69
XRCC5	LDLRAD4	-0.4865959	0.6105527	1.0971486	0.69
PARPBP	MYO19	0.83737975	-0.2594363	1.09681605	0.69
LDLRAD4	PRICKLE1	0.6908707	-0.39948794	1.09035864	0.69
COL4A1	ACTN3	0.45797643	-0.6141607	1.07213713	0.69
OPRK1	LDLRAD4	-0.61761147	0.4482097	1.06582117	0.69
FAM129B	TIMM23	0.4257001	-0.61983716	1.04553726	0.69
NGFR	TMOD2	-0.39564335	0.64618427	1.04182762	0.69
APC	STX17	-0.4837778	0.5566598	1.0404376	0.69
ZWINT	LDLRAD4	-0.7026666	0.3311873	1.0338539	0.69
MCM6	LDLRAD4	-0.5184038	0.5024719	1.0208757	0.69
LDLRAD4	GALNT2	-0.514632	0.4986503	1.0132823	0.69
LDLRAD4	CCT3	-0.5140807	0.49695292	1.01103362	0.69
ESRRG	SORBS1	-0.54498845	0.73174226	1.27673071	0.689
ZWINT	SCML2	0.4276383	-0.6348283	1.0624666	0.689
CBX3	DDX39A	-0.2496942	0.7946061	1.0443003	0.689
ESRRG	IL4	0.7409403	-0.3611838	1.1021241	0.688
CADM1	PRKRIR	-0.4563288	0.54857373	1.00490253	0.688
APC	ZWINT	-0.28098065	0.8400697	1.12105035	0.687
UBE2H	CBX3	0.40497738	-0.6029431	1.00792048	0.687
MUC1	CTNNA1	-0.85999155	0.39518526	1.25517681	0.686
KIAA0101	MOCOS	0.71442765	-0.34855816	1.06298581	0.686
XRCC5	RGS5	-0.6820114	0.4670268	1.1490382	0.685
UBC	PNPT1	-0.3008409	0.7290713	1.0299122	0.683
KARS	CSDE1	-0.5289772	0.5211677	1.0501449	0.682
GRWD1	FOXP2	-0.58140016	0.47807494	1.0594751	0.681
ABI2	IFT81	0.582227	-0.50003743	1.08226443	0.68

UBC	ZWINT	-0.5954513	0.4346751	1.0301264	0.679
PARK2	FTL	-0.56185335	0.77378285	1.3356362	0.678
WWOX	CADM1	-0.532145	0.57086766	1.10301266	0.678
KLF4	SCML2	-0.41872182	0.6661863	1.08490812	0.675
RUFY3	NDFIP2	-0.6085199	0.5061176	1.1146375	0.674
SP3	HDAC1	-0.43330374	0.6773109	1.11061464	0.673
SMAD7	CADM1	-0.3598246	0.69732666	1.05715126	0.673
LTA	CD40	0.601374	-0.45353138	1.05490538	0.672
IGF2R	ACTN3	0.3440902	-0.82413274	1.16822294	0.671
ESRRG	DNAJB2	-0.7207885	0.29283985	1.01362835	0.671
RACGAP1	SNX18	-0.39255902	0.70535016	1.09790918	0.67
BLOC1S6	ABI2	0.50106245	-0.54237187	1.04343432	0.67
ORC5	ABI2	0.6011006	-0.40033844	1.00143904	0.67
SFN	RPS2	0.6195587	-0.51567465	1.13523335	0.669
STX7	STK10	0.51113576	-0.58835524	1.099491	0.668
IGF2R	NUDT21	-0.4874406	0.70636874	1.19380934	0.667
CSTF3	TOP2A	0.6826116	-0.47755593	1.16016753	0.667
EPS15	SNRPB2	-0.41180673	0.6403269	1.05213363	0.667
TBL1X	RGS3	-0.27797696	0.7365691	1.01454606	0.667
PTTG1IP	TOP3A	-0.7134585	0.6479284	1.3613869	0.666
SERBP1	TOP2A	0.8048916	-0.31118912	1.11608072	0.665
ITSN1	STX6	-0.7359009	0.35485414	1.09075504	0.665
ANXA11	ELOVL5	-0.58424765	0.47362718	1.05787483	0.665
KLF12	TIMELESS	0.39968038	-0.6392183	1.03889868	0.665
PTPN2	CTNNA1	-0.63019276	0.55935156	1.18954432	0.664
TOR1AIP1	GSTM5	0.47559306	-0.62589824	1.1014913	0.664
SRSF1	NID2	-0.4202769	0.7032825	1.1235594	0.663
DCN	KANK1	0.7766999	-0.2558911	1.032591	0.662
HDAC11	DNAJC9	-0.7757294	0.30704254	1.08277194	0.661
DDX6	TUFM	-0.2598255	0.81133866	1.07116416	0.661
PPIA	HLA-C	-0.36266574	0.66943187	1.03209761	0.661
CSTF3	HLA-G	-0.59477663	0.783978	1.37875463	0.66
VCAM1	STX7	0.5451238	-0.6361771	1.1813009	0.66
MYOC	NAB2	0.44379443	-0.59403217	1.0378266	0.66
NAP1L1	PEX5	-0.49201137	0.63946354	1.13147491	0.659
SCRN1	TPM4	0.45293704	-0.6347734	1.08771044	0.659
ZNF148	HDAC1	-0.5784274	0.6275175	1.2059449	0.658
HINFP	ZNF704	0.55269563	-0.57949	1.13218563	0.658
RAB11A	OPTN	-0.6281144	0.39885807	1.02697247	0.658
WBP4	ARHGEF12	-0.8427173	0.15959619	1.00231349	0.658
LAMA4	POLDIP3	-0.68610704	0.59920406	1.2853111	0.657
KARS	AHNAK	-0.78242654	0.3547428	1.13716934	0.657
RANGAP1	PNPT1	0.58743787	-0.52351886	1.11095673	0.657
QKI	MOCOS	-0.59548956	0.45025384	1.0457434	0.657
SERBP1	SPG20	-0.57173276	0.61413366	1.18586642	0.656
NUP62	BFSP1	-0.5619732	0.5289272	1.0909004	0.656
ATP1A2	PRUNE2	0.521971	-0.57466865	1.09663965	0.655
ESRRG	PRRC2A	0.6343329	-0.588512	1.2228449	0.654
TOP2A	EXOSC8	0.60555494	-0.5838029	1.18935784	0.654
BZW2	TEFM	-0.6958247	0.4695608	1.1653855	0.654
RCC2	HLA-B	-0.53295356	0.61733496	1.15028852	0.654
ESRRG	QKI	-0.34581143	0.71304744	1.05885887	0.654
ESRRG	RTN4	-0.5156167	0.709066	1.2246827	0.652
APC	TMED5	-0.43034574	0.59760207	1.02794781	0.652
CAST	XRCC5	-0.4013713	0.828773	1.2301443	0.651
RUVBL2	MCM4	0.8200741	-0.3568373	1.1769114	0.651

HIVEP3	ZNF467	0.50646406	-0.5126283	1.01909236	0.651
LAMA1	COL5A1	-0.40478745	0.7146711	1.11945855	0.65
TOP2A	DNMT1	0.6709932	-0.44785297	1.11884617	0.65
SERBP1	GTF2I	-0.6768561	0.33753642	1.01439252	0.65
EPS8	FANCI	-0.6969327	0.36316445	1.06009715	0.649
STAT5B	SOX4	0.54572093	-0.73821187	1.2839328	0.647
SERPINA1	ESRRG	0.53184414	-0.53754646	1.0693906	0.647
AMOT	HSPA14	0.30245754	-0.7239107	1.02636824	0.647
CYTH2	PHLDB2	0.54781055	-0.4710974	1.01890795	0.646
CCT3	KCNRG	0.6988981	-0.67004406	1.36894216	0.645
GTF2I	STX6	-0.7897	0.52001107	1.30971107	0.645
ARPC5L	CSRP2BP	-0.65855473	0.48565644	1.14421117	0.645
ZWINT	KATNAL1	-0.69113505	0.4427822	1.13391725	0.645
VEGFA	PFDN4	0.3751019	-0.66815436	1.04325626	0.645
DKC1	TOP2A	0.7207499	-0.5431717	1.2639216	0.644
GNAQ	RACGAP1	-0.53943247	0.63008803	1.1695205	0.644
LRRC16A	LZTS1	-0.5200607	0.51440674	1.03446744	0.644
CSTF3	HLA-B	-0.40256575	0.86526024	1.26782599	0.643
TUFM	HLA-C	-0.5745392	0.6098776	1.1844168	0.643
GNAL	ARPP21	-0.5579378	0.5816815	1.1396193	0.643
MAP1B	TRIM14	0.56764406	-0.4892745	1.05691856	0.643
SLC37A4	SLC46A3	-0.6315035	0.39373267	1.02523617	0.643
KLF12	CCT3	0.21877795	-0.8040697	1.02284765	0.643
IGF1	AP3M1	-0.7526485	0.45096508	1.20361358	0.642
PCCB	CEACAM1	0.53249294	-0.4782593	1.01075224	0.642
UBC	PCNA	-0.5420241	0.6319789	1.174003	0.641
PDE4DIP	PDHB	-0.3073981	0.74493796	1.05233606	0.64
SEPT11	PARP14	0.3131131	-0.7276701	1.0407832	0.64
SERPINA1	COL5A1	-0.50202477	0.8716276	1.37365237	0.639
APC	NUTF2	-0.61029077	0.65565896	1.26594973	0.639
HDAC1	GTF2I	-0.7143855	0.33260393	1.04698943	0.639
SNAP29	DBI	-0.29147232	0.8037116	1.09518392	0.638
RAB35	RAB11A	0.7514887	-0.29974625	1.05123495	0.638
PDE4DIP	RACGAP1	-0.5779714	0.4925445	1.0705159	0.637
QKI	MED31	-0.43937474	0.5682688	1.00764354	0.637
MAP1B	CD40	0.49613935	-0.67409074	1.17023009	0.636
KSR1	CCT3	-0.55880284	0.6099007	1.16870354	0.636
SET	KIRREL	-0.7055112	0.40080148	1.10631268	0.636
HTT	OPRK1	-0.63296664	0.42336932	1.05633596	0.636
DLG1	CADM1	-0.4899295	0.51186776	1.00179726	0.636
GNPTAB	TIRAP	-0.48869073	0.63363916	1.12232989	0.635
VCAM1	AIDA	0.25666496	-0.8242073	1.08087226	0.635
APC	MED31	-0.5342431	0.66795975	1.20220285	0.634
TOP2A	PNPT1	0.65077364	-0.6601712	1.31094484	0.632
APC	PSMB3	-0.51573616	0.6981604	1.21389656	0.632
MRPS14	PHF5A	-0.43329734	0.6164956	1.04979294	0.631
ZNF426	EFEMP2	0.70492	-0.30955058	1.01447058	0.631
CNKSR2	ARHGEF12	-0.700485	0.357958	1.058443	0.63
CAST	ABI2	0.35160077	-0.6891022	1.04070297	0.63
SCML2	PER3	-0.60513276	0.5776438	1.18277656	0.629
SFN	PSMB3	0.6704302	-0.50959593	1.18002613	0.629
APC	AGPAT5	-0.46867555	0.5509405	1.01961605	0.628
TBL1XR1	MLST8	0.44174793	-0.67253107	1.114279	0.627
ELOVL6	TNS1	0.7976853	-0.24186252	1.03954782	0.627
DCUN1D5	KLF12	0.31545037	-0.86744386	1.18289423	0.626
PALLD	MAP4	0.6354828	-0.43082902	1.06631182	0.626

NOD1	CASP2	0.37777826	-0.6510643	1.02884256	0.626
TNC	THBS1	0.48590118	-0.57446855	1.06036973	0.625
GNAQ	CNOT11	-0.55279833	0.73550224	1.28830057	0.624
CSNK2A1	QKI	-0.47369668	0.80256504	1.27626172	0.624
CTNNA1	ELF4	-0.32121375	0.7762791	1.09749285	0.624
HSP90AA1	HNRNPC	0.4835374	-0.57838184	1.06191924	0.624
PARP9	NTNG2	0.2547608	-0.7639992	1.01876	0.624
CSNK2A1	CLASP2	0.17319344	-0.8492957	1.02248914	0.623
UBE2H	PFDN4	-0.4290003	0.590329	1.0193293	0.623
ADAM10	CTNNA1	-0.52761173	0.6141549	1.14176663	0.622
PPP3CB	CNOT11	-0.47362438	0.65018284	1.12380722	0.622
KATNAL1	CNOT11	-0.43312544	0.68765	1.12077544	0.622
KIAA0101	SCML2	0.48726758	-0.5264521	1.01371968	0.622
EPS15	RACGAP1	-0.6537358	0.72451884	1.37825464	0.621
UBE2B	RTN4	0.8929009	-0.31329998	1.20620088	0.621
PCNA	QKI	-0.6723786	0.51972324	1.19210184	0.621
UBC	ESRRG	-0.5287906	0.49817026	1.02696086	0.621
VCAM1	SEPT11	0.52393156	-0.6083982	1.13232976	0.62
TRIM2	BCAR1	-0.6255761	0.46121898	1.08679508	0.62
ZWINT	ZHX2	-0.55164146	0.48557505	1.03721651	0.62
SH3PXD2A	PDLIM4	-0.5195822	0.53528446	1.05486666	0.619
CTNNA1	ADAMTS3	-0.68176335	0.5528184	1.23458175	0.618
XRCC5	CPNE3	-0.647098	0.5718199	1.2189179	0.618
KARS	PDE4DIP	-0.591933	0.6040287	1.1959617	0.618
ESRRG	MLC1	0.71634066	-0.4426236	1.15896426	0.618
TOP2A	HDAC1	0.72864985	-0.424231	1.15288085	0.618
ZHX2	CEP68	0.5992194	-0.44353366	1.04275306	0.618
PTPRN2	SLAMF1	-0.6077708	0.41324285	1.02101365	0.618
SNRPC	VAMP4	-0.61024857	0.5652271	1.17547567	0.616
RACGAP1	KANK1	-0.6832969	0.40213504	1.08543194	0.616
CST2	RCC2	0.4302196	-0.6264897	1.0567093	0.616
SCARB2	RPL14	-0.22240694	0.7931292	1.01553614	0.616
GSTT1	CIT	-0.5345158	0.47136754	1.00588334	0.616
ARNTL	MLXIP	-0.6965945	0.5013171	1.1979116	0.615
KPNA4	LIF	0.31533054	-0.7251153	1.04044584	0.615
DDX20	SUFU	-0.5386305	0.6417277	1.1803582	0.613
MIR3917	NUDT21	0.69483787	-0.3623577	1.05719557	0.613
SH3PXD2A	TAB2	-0.74847454	0.44730538	1.19577992	0.612
EZH2	TOP2A	0.9200974	-0.20291199	1.12300939	0.611
TOP2A	NOP2	0.6007039	-0.4695227	1.0702266	0.61
DNMT1	RTN4	-0.6279068	0.40212762	1.03003442	0.609
SMYD3	MDN1	-0.7415718	0.5442571	1.2858289	0.608
STX8	RACGAP1	-0.72435915	0.4225021	1.14686125	0.608
APC	TRMT1	-0.51154655	0.6210714	1.13261795	0.608
NAP1L1	CD40	-0.6689553	0.37472323	1.04367853	0.608
PLCB4	NUDCD2	-0.6758708	0.3412433	1.0171141	0.608
KLF12	MED31	0.4543897	-0.70844424	1.16283394	0.607
SRSF1	WAC	-0.41110066	0.6777352	1.08883586	0.607
SNX5	GALNT2	-0.59128505	0.481777	1.07306205	0.607
PPP2CB	ESRRG	-0.41394582	0.64143634	1.05538216	0.607
KARS	OSR2	-0.7589206	0.56658095	1.32550155	0.606
NAALADL2	GGCT	-0.6228409	0.67354214	1.29638304	0.606
ANKRD28	MTHFD1L	-0.39404166	0.801708	1.19574966	0.606
C4A	VANGL1	-0.54589427	0.629502	1.17539627	0.606
XPNPEP1	DFNB31	-0.31586328	0.7643926	1.08025588	0.606
TUFM	CRY2	-0.6256237	0.48006207	1.10568577	0.605

MCM6	HLA-B	-0.44576102	0.6338162	1.07957722	0.605
SEPT11	TGM2	0.36117458	-0.6524439	1.01361848	0.605
GALNT2	HIST1H2BE	0.66363394	-0.34017998	1.00381392	0.605
CLIP1	GPC1	0.7214304	-0.4187191	1.1401495	0.604
CDK6	IL1A	-0.6573744	0.635505	1.2928794	0.603
USP4	PIK3IP1	-0.6369051	0.49503773	1.13194283	0.603
TCF3	C1QBP	0.6729451	-0.41988254	1.09282764	0.602
NTRK2	HSP90AA1	-0.6441401	0.4206457	1.0647858	0.602
HSP90AA1	PPIB	0.52669966	-0.5197287	1.04642836	0.602
TRIAP1	TLN2	-0.48838192	0.5409843	1.02936622	0.602
ASB6	ASB14	0.27414504	-0.7462184	1.02036344	0.602
AGPAT5	ASB1	-0.25459918	0.7628178	1.01741698	0.602
SET	RANGAP1	0.6226386	-0.5360075	1.1586461	0.601
NXF1	NUP62	-0.4826116	0.5684447	1.0510563	0.601
DCN	MAP6	0.6892586	-0.35560963	1.04486823	0.601
KLF12	DIEXF	0.21240428	-0.8021883	1.01459258	0.601
BID	BCL2	-0.6409309	0.41418755	1.05511845	0.6
NAB2	AIDA	0.6567966	-0.34470737	1.00150397	0.6
SFN	RACGAP1	0.6797043	-0.48782387	1.16752817	0.599
RELB	C1QBP	0.4056167	-0.71522164	1.12083834	0.599
CYBRD1	GSTM5	0.7474644	-0.3110563	1.0585207	0.599
CSK	DDB2	-0.33645743	0.71927327	1.0557307	0.599
SERBP1	KLF12	0.39778247	-0.6678063	1.06558877	0.598
UBC	NUP62	-0.48331717	0.5552413	1.03855847	0.598
RPN2	AUP1	-0.26980424	0.7427143	1.01251854	0.598
UBC	TEC	0.75500816	-0.5243374	1.27934556	0.597
ADAM10	ABI2	0.55411416	-0.44971368	1.00382784	0.597
LTF	SLPI	0.43467492	-0.565942	1.00061692	0.597
VEGFA	POLN	0.37214333	-0.73089737	1.1030407	0.596
TUBB	PFDN4	0.48534355	-0.605056	1.09039955	0.596
FKBP1A	CXCL2	0.6416518	-0.39799577	1.03964757	0.596
PPIL2	STX6	-0.35221702	0.6710907	1.02330772	0.596
ZWINT	ZHX3	-0.71350884	0.29305294	1.00656178	0.596
HSPH1	KIRREL	-0.66973156	0.33522448	1.00495604	0.596
LAMA1	STK36	-0.48783937	0.69827884	1.18611821	0.595
ESRRG	MID2	-0.45287892	0.7001661	1.15304502	0.595
UBC	DNMT1	-0.32775608	0.70042276	1.02817884	0.595
SLU7	IFT81	0.57286364	-0.4429489	1.01581254	0.595
FOXN3	BID	-0.7600835	0.3725654	1.1326489	0.594
S100B	NPC1	0.4037654	-0.65197974	1.05574514	0.594
SNRPB2	SCARB2	-0.42779824	0.613042	1.04084024	0.594
PDIA4	BCL2L1	0.41521782	-0.7573309	1.17254872	0.593
CSNK2A1	MAP4	-0.59042674	0.5670053	1.15743204	0.593
KARS	LMNB2	0.79333794	-0.37594557	1.16928351	0.592
PCNA	WBP4	-0.4142487	0.70819056	1.12243926	0.592
WIPI2	DNAJB1	0.72459245	-0.37055448	1.09514693	0.592
KIRREL	LCLAT1	-0.44309118	0.83655965	1.27965083	0.591
OPRK1	C5AR1	-0.47325298	0.53015894	1.00341192	0.591
ESRRG	TBC1D9B	-0.80233085	0.58972096	1.39205181	0.59
ESRRG	THRB	-0.5717415	0.6863906	1.2581321	0.59
EPS15	NPC1	-0.4847582	0.7267547	1.2115129	0.59
PSMB2	CRTAP	-0.43968195	0.74788505	1.187567	0.59
BUB1B	PSMB2	0.754086	-0.31043255	1.06451855	0.59
KCNRG	LONRF2	-0.6177186	0.38886985	1.00658845	0.59
IPO11	ELOVL6	-0.6548725	0.4017521	1.0566246	0.589
NVL	HLA-C	-0.65263045	0.56034464	1.21297509	0.588

COL5A1	FCN1	-0.34237522	0.8476598	1.19003502	0.587
PALLD	URI1	-0.4685031	0.56289935	1.03140245	0.587
TRIM27	EGR1	0.5319095	-0.4959182	1.0278277	0.587
ENG	BCAR1	0.5847919	-0.4317761	1.016568	0.587
CAST	SCML2	-0.8043349	0.20574313	1.01007803	0.587
MAPK13	APP	-0.30505916	0.69958514	1.0046443	0.587
MMP14	CADM1	0.76475376	-0.57371956	1.33847332	0.586
PCNA	HLA-C	-0.54737633	0.6588227	1.20619903	0.586
PCNA	HLA-B	-0.36643443	0.6853435	1.05177793	0.586
ASS1	GTF2I	-0.30180913	0.7484409	1.05025003	0.586
TLN2	GALNT2	-0.5867796	0.41852158	1.00530118	0.586
SSRP1	MCM4	0.79677445	-0.28996253	1.08673698	0.585
XPO1	STK10	-0.45359528	0.63242817	1.08602345	0.585
YARS	VAMP4	-0.5453731	0.53137356	1.07674666	0.585
RPS21	NUDCD2	0.37932038	-0.64150405	1.02082443	0.584
MCM6	QKI	-0.7469317	0.4281812	1.1751129	0.583
NDRG2	JUND	0.66230136	-0.40307567	1.06537703	0.583
MOCOS	SHMT2	0.696562	-0.36385238	1.06041438	0.583
RACGAP1	ARHGEF2	-0.72430295	0.33197173	1.05627468	0.583
RBM10	RELA	-0.35043028	0.70381635	1.05424663	0.583
PGRMC2	COL5A1	0.5299159	-0.513119	1.0430349	0.583
ALDOB	PCNA	0.49608478	-0.527948	1.02403278	0.583
CASP6	CEACAM1	-0.6031079	0.5189782	1.1220861	0.582
PALLD	ENG	0.38916737	-0.6409297	1.03009707	0.582
UBC	RACGAP1	-0.46169287	0.56786346	1.02955633	0.582
ITSN1	ESRRG	-0.66610426	0.66731447	1.33341873	0.581
B2M	DHX30	-0.6630218	0.63950795	1.30252975	0.581
APC	TRIM27	-0.56901246	0.6068926	1.17590506	0.58
EPS8	NUDT21	-0.5303873	0.55078363	1.08117093	0.58
CSNK2A1	SPTBN1	-0.6092908	0.4678246	1.0771154	0.58
HSP90AA1	H2AFY	0.39460644	-0.68046486	1.0750713	0.58
RRM1	FARSA	-0.2662156	0.765486	1.0317016	0.58
SCML2	PER2	-0.7892402	0.5836983	1.3729385	0.579
CDC25B	MDN1	-0.39749587	0.6932275	1.09072337	0.579
FKBP1A	GABBR1	-0.43757498	0.6334294	1.07100438	0.579
MCM6	RUFY3	-0.7507518	0.5137244	1.2644762	0.578
GSTP1	RACGAP1	-0.66346496	0.58140224	1.2448672	0.578
XPO1	POSTN	-0.5100051	0.68519276	1.19519786	0.578
ADAM10	PFDN4	0.5455552	-0.6489095	1.1944647	0.578
PARK2	KCND3	-0.6510445	0.44853646	1.09958096	0.578
PTTG1	CDC37L1	-0.77453375	0.22637203	1.00090578	0.578
APC	EIF2B2	-0.53113896	0.4817596	1.01289856	0.577
ALAD	GALNT2	-0.48295513	0.67143	1.15438513	0.576
USP4	NXF1	-0.6068722	0.5157963	1.1226685	0.576
MED19	MED16	-0.37384227	0.6783539	1.05219617	0.576
ESRRG	GRB14	-0.27712205	0.77323735	1.0503594	0.576
PELP1	NOL9	0.4768944	-0.54959106	1.02648546	0.576
TRAPPC3	ZBTB7A	-0.5883453	0.48621237	1.07455767	0.575
CSNK2A1	PGRMC2	-0.34943253	0.6656352	1.01506773	0.575
ESRRG	AGFG1	-0.6163158	0.5793373	1.1956531	0.574
PDIA4	CLSTN1	-0.21075134	0.8411412	1.05189254	0.574
CBX3	XPO5	-0.15654816	0.87864614	1.0351943	0.574
SYNPO	RTN4RL1	-0.59227926	0.43174714	1.0240264	0.574
SP3	RCC2	-0.7283448	0.7502496	1.4785944	0.573
ESRRG	DLG1	-0.56595933	0.67561066	1.24156999	0.573
BCL2L1	DNMT1	0.39891997	-0.6843761	1.08329607	0.573

TRA2B	CHD1L	0.37040603	-0.83092296	1.20132899	0.572
PRMT5	PCMTD1	-0.6645098	0.4055175	1.0700273	0.571
HDAC1	HDAC11	-0.74961543	0.27786276	1.02747819	0.571
PALLD	LTBP4	0.3567859	-0.6452331	1.002019	0.571
CLIP1	EIF4G3	0.6190065	-0.6013059	1.2203124	0.57
GSTM3	URI1	-0.6261838	0.5561902	1.182374	0.569
SMYD3	AACS	-0.59811556	0.46663427	1.06474983	0.569
OPRK1	GPR34	-0.69782233	0.6470283	1.34485063	0.567
EPS15	GAPDH	-0.73043734	0.4155373	1.14597464	0.567
UBC	HDAC1	-0.634058	0.5047849	1.1388429	0.567
TAF1C	RIC1	-0.36899817	0.6390366	1.00803477	0.567
ESRRG	APOD	-0.6827962	0.47703657	1.15983277	0.566
SPPL2A	RIN3	0.3932637	-0.6911485	1.0844122	0.566
EIF5	KLF12	0.2871113	-0.7812882	1.0683995	0.566
GSTM5	ZHX3	0.73642725	-0.31890857	1.05533582	0.566
STAT5B	C8orf4	-0.5565181	0.4750577	1.0315758	0.565
GRB14	MLST8	0.5475147	-0.49806637	1.04558107	0.564
CSNK2A1	CALM1	-0.3026342	0.7016945	1.0043287	0.564
HDAC11	CCT3	-0.70223165	0.4053549	1.10758655	0.563
SERBP1	IRF9	-0.5328789	0.5276755	1.0605544	0.562
METTL3	HIST1H2BE	-0.39994228	0.6269651	1.02690738	0.562
BID	ZHX3	-0.6931899	0.5142995	1.2074894	0.561
XRCC5	CBX3	-0.46476158	0.57459176	1.03935334	0.561
PIKFYVE	ACTN3	-0.4993772	0.5250647	1.0244419	0.561
DLG1	EIF4G3	0.7213695	-0.49307704	1.21444654	0.56
SAP30L	STAT3	-0.48826304	0.6587151	1.14697814	0.56
USP9X	STX6	-0.46326324	0.67200565	1.13526889	0.56
RBPJ	BST2	-0.532398	0.56374615	1.09614415	0.56
PDIA4	LRWD1	0.5395261	-0.51876646	1.05829256	0.56
PCNA	RUFY3	-0.74981827	0.7793764	1.52919467	0.558
PCNA	ITCH	-0.64811224	0.50884974	1.15696198	0.558
RSPO3	C10orf90	-0.8119126	0.37814882	1.19006142	0.557
GSTP1	ZWINT	-0.5672679	0.58431834	1.15158624	0.557
KARS	ALAD	-0.52936184	0.58945954	1.11882138	0.557
NSMF	MYO19	-0.27359405	0.8279819	1.10157595	0.557
INTS6	LZTS1	-0.6230984	0.4609288	1.0840272	0.557
MYOM1	GALNT2	-0.7358197	0.33484095	1.07066065	0.557
LMOD3	DNAJC9	0.42174283	-0.6138938	1.03563663	0.557
DHX30	HLA-C	-0.34993175	0.8517271	1.20165885	0.556
DCN	ZNF219	0.62513894	-0.39868784	1.02382678	0.556
ITCH	BID	-0.6429665	0.36498407	1.00795057	0.556
XRCC5	THRB	-0.7427294	0.48075923	1.22348863	0.555
GNAQ	TEC	0.5640928	-0.51608664	1.08017944	0.555
RPS20	NAB2	0.5995236	-0.43772703	1.03725063	0.555
ZHX1	BID	-0.53336537	0.47427258	1.00763795	0.555
RPL27A	RPN2	0.5089794	-0.60281783	1.11179723	0.554
NFE2L1	ARPC5L	-0.745861	0.36297947	1.10884047	0.554
TRIAP1	S100A16	0.7716256	-0.24383022	1.01545582	0.554
SSBP2	PEX5	-0.55529904	0.668944	1.22424304	0.553
PJA2	MALT1	-0.8521082	0.3259926	1.1781008	0.553
ZNF385D	HINFP	-0.55193686	0.5242294	1.07616626	0.553
PALLD	FOXJ2	-0.28776047	0.7746882	1.06244867	0.553
SNRPB2	EPS8	-0.44844052	0.58005977	1.02850029	0.553
KIF13A	GSTM5	0.78473055	-0.36142126	1.14615181	0.552
APC	KANSL2	-0.5523659	0.4855669	1.0379328	0.552
CADM1	RHOQ	-0.29809403	0.70735776	1.00545179	0.552

CFH	LEPR	0.55297995	-0.6037897	1.15676965	0.551
EPS15	EIF2B2	-0.4028363	0.73950815	1.14234445	0.551
VAV2	MOCOS	0.44394985	-0.5582598	1.00220965	0.551
CBS	SERPINE2	-0.5597397	0.5472537	1.1069934	0.55
UBE2B	AHCYL1	0.8783563	-0.15404168	1.03239798	0.55
APC	HNRNPC	-0.5265743	0.5624219	1.0889962	0.548
RRBP1	FOS	0.73889977	-0.5868063	1.32570607	0.547
SCRN1	BMP1	0.7231019	-0.45920885	1.18231075	0.547
UBE2B	ATP6V1G1	-0.48814714	0.60152346	1.0896706	0.547
KAT6B	MAP4	0.56209	-0.5251118	1.0872018	0.547
HDAC11	BID	-0.73573256	0.4132407	1.14897326	0.546
ADAMTS9	TLL2	0.5437577	-0.478506	1.0222637	0.546
FBN1	THBS3	0.67843425	-0.3230952	1.00152945	0.545
PPIL2	AMOT	0.71436757	-0.5507488	1.26511637	0.544
EBF1	SLIRP	-0.6768384	0.49922705	1.17606545	0.544
PIK3R1	RPL14	-0.3694979	0.7096241	1.079122	0.544
GALNT2	CST1	0.46672082	-0.73749727	1.20421809	0.543
DDX6	MOCOS	-0.75473726	0.37804753	1.13278479	0.543
PCNA	TK1	0.8228824	-0.30838484	1.13126724	0.543
KLF12	ADARB1	0.34229413	-0.7087858	1.05107993	0.543
ITCH	NUDT21	-0.4098869	0.6026237	1.0125106	0.543
PTPRG	NTSR2	0.42506647	-0.67199826	1.09706473	0.542
KCNK3	PDLIM4	-0.60294104	0.45831183	1.06125287	0.542
MCM4	RACGAP1	0.82585716	-0.20700018	1.03285734	0.542
MCM4	BST2	0.57062817	-0.69127506	1.26190323	0.541
FOXM1	NDFIP2	0.7235189	-0.5082417	1.2317606	0.541
TRIO	SERPINE2	-0.6653005	0.460458	1.1257585	0.541
ENG	SOX30	0.296265	-0.73232484	1.02858984	0.541
SHMT1	PGF	-0.73984903	0.473753	1.21360203	0.54
BYSL	HDAC1	0.7240958	-0.47808442	1.20218022	0.54
FOXN3	PSMA4	-0.6483671	0.4646596	1.1130267	0.54
FOXN3	PSMB3	-0.6873873	0.37733114	1.06471844	0.54
SORL1	SHANK2	0.5011979	-0.5509091	1.052107	0.54
DNAJC15	ARHGEF12	-0.60403	0.42188603	1.02591603	0.54
YARS	KLF12	0.33122638	-0.68156374	1.01279012	0.54
KIF5B	IPO9	0.39395198	-0.61545825	1.00941023	0.539
MAPKAP1	GET4	-0.51234984	0.59840006	1.1107499	0.538
CSF3R	FGF18	-0.63752395	0.45956895	1.0970929	0.538
PPM1A	PSMA4	-0.660219	0.42957717	1.08979617	0.538
LIMK2	SAFB2	-0.453538	0.6059376	1.0594756	0.538
PPM1A	PSMB3	-0.62532	0.39023435	1.01555435	0.538
RACGAP1	KCNRG	0.6094859	-0.68615985	1.29564575	0.537
CSTF3	POLN	0.39007404	-0.70415616	1.0942302	0.537
CHMP3	PDHB	-0.42775026	0.59885186	1.02660212	0.537
URI1	FBXO9	0.7017003	-0.43823206	1.13993236	0.536
ARHGEF12	ZHX2	-0.8713973	0.25804472	1.12944202	0.536
CDK6	CHURC1-FNTB	-0.47587612	0.58088917	1.05676529	0.536
CHD2	JDP2	0.33857828	-0.7098905	1.04846878	0.536
DYSF	STK36	-0.44145682	0.5694143	1.01087112	0.536
RELB	NUDT21	0.44620797	-0.6092139	1.05542187	0.535
NOL9	ASB1	-0.55212194	0.8078885	1.36001044	0.534
APC	XPO5	-0.5323083	0.5820152	1.1143235	0.534
ITSN1	LTF	-0.6379661	0.46220395	1.10017005	0.533
USP4	RAB13	0.75192463	-0.397662	1.14958663	0.532
CYBRD1	PSMB3	-0.6525976	0.43540448	1.08800208	0.532
ESRRG	MPL	-0.34488168	0.732917	1.07779868	0.532

CTNNA1	CELSR1	-0.4056062	0.665527	1.0711332	0.532
ZNF467	ZNF668	-0.32876667	0.70259833	1.031365	0.532
CADM1	KLHL17	0.3258665	-0.67667055	1.00253705	0.532
APC	MAP4K4	-0.6490401	0.51782197	1.16686207	0.531
WNK1	CADM1	-0.49021128	0.64046866	1.13067994	0.531
DDB2	ARPC1B	-0.35592726	0.80492336	1.16085062	0.53
DHX30	PARP14	-0.41891614	0.6270429	1.04595904	0.53
XPO1	TBC1D1	-0.47593957	0.536549	1.01248857	0.53
XRCC5	PDE4DIP	-0.73589355	0.4822489	1.21814245	0.529
EIF6	DNAJC15	-0.46461016	0.72606474	1.1906749	0.529
ZNF385D	LBP	0.7586787	-0.40264794	1.16132664	0.529
RBMS3	RPL27A	-0.75034046	0.2569855	1.00732596	0.529
SET	APOE	-0.5876586	0.65531695	1.24297555	0.526
CEACAM1	ARPP19	-0.24685712	0.88734627	1.13420339	0.526
PRKRIR	FZD10	0.5740327	-0.5562316	1.1302643	0.526
KIF5B	PRRC2A	0.42077848	-0.6658427	1.08662118	0.525
CAST	ESRRG	-0.71303475	0.36917806	1.08221281	0.525
PTPRG	RANGAP1	0.28849638	-0.75415957	1.04265595	0.525
SH3PXD2A	USP4	0.56821656	-0.53590643	1.10412299	0.524
DNMT1	PCMTD1	-0.71268237	0.5099577	1.22264007	0.523
NFE2L1	MICALL1	-0.36308724	0.7570519	1.12013914	0.523
EIF4G3	BRAP	0.48156443	-0.57743734	1.05900177	0.523
ADA	LUC7L3	-0.4742921	0.5760813	1.0503734	0.523
NXF1	MAPK13	-0.30400467	0.69970286	1.00370753	0.523
PTPN11	CADM1	-0.60741264	0.4453332	1.05274584	0.522
PCNA	EBF1	-0.90139437	0.24223669	1.14363106	0.521
TMOD3	LAMA1	0.7644256	-0.36480615	1.12923175	0.521
KIF13A	TBRG4	-0.5868999	0.47588605	1.06278595	0.521
PRMT1	CBX3	-0.19390346	0.82464266	1.01854612	0.521
XRCC5	ST13	-0.5314729	0.602935	1.1344079	0.52
IGF1R	ELOVL5	-0.5569788	0.526405	1.0833838	0.52
SORL1	WWC1	0.552252	-0.4535627	1.0058147	0.52
PCNA	USP9X	-0.36636275	0.6342602	1.00062295	0.52
SAP30L	XPO5	-0.63205475	0.59834296	1.23039771	0.519
DKC1	IGF2R	-0.7505311	0.38575688	1.13628798	0.519
GPHN	LCLAT1	-0.60920286	0.45559156	1.06479442	0.519
MDM1	LMOD3	0.67617935	-0.4382559	1.11443525	0.518
ADAM10	KCNE3	0.36800933	-0.6861812	1.05419053	0.518
CHD2	AIDA	0.48558083	-0.5502739	1.03585473	0.518
CBX4	ARHGEF2	-0.3969752	0.77885234	1.17582754	0.517
GEM	LTBP4	0.7546099	-0.34157214	1.09618204	0.517
PIAS2	PSMA4	-0.41506597	0.636161	1.05122697	0.517
GNAQ	FUT11	-0.3656538	0.67132443	1.03697823	0.517
SFN	SNU13	0.64818513	-0.6103026	1.25848773	0.516
COL3A1	PRMT8	-0.83920074	0.41447073	1.25367147	0.516
VCAM1	PPIL2	-0.5320185	0.7362928	1.2683113	0.515
KIRREL	LRP8	-0.7324652	0.27639434	1.00885954	0.515
TYRO3	TNIP1	-0.2267862	0.77730703	1.00409323	0.515
KIRREL	LCMT2	-0.6944882	0.69140863	1.38589683	0.514
SETD7	FZD10	0.27529296	-0.778769	1.05406196	0.514
CSNK2A1	KCNQ4	-0.6339098	0.5891574	1.2230672	0.513
AUP1	TEAD4	-0.4214954	0.72132355	1.14281895	0.513
ESRRG	NPR3	-0.2580772	0.8488113	1.1068885	0.513
SOD2	GABARAPL1	-0.59948707	0.50114375	1.10063082	0.513
TRIM27	CSRP2BP	-0.37207332	0.706193	1.07826632	0.513
MDN1	HDAC1	-0.5081038	0.5678982	1.076002	0.513

SIX1	CADM1	-0.28597906	0.720879	1.00685806	0.513
WWC1	LMOD3	-0.6587433	0.46138453	1.12012783	0.512
GNAQ	AGPAT5	-0.46876568	0.68987596	1.15864164	0.511
NEDD4L	ARPC5L	-0.71472716	0.34648997	1.06121713	0.511
PPM1A	BMP1	0.41559735	-0.584458	1.00005535	0.511
MVP	FOS	0.53318423	-0.67943096	1.21261519	0.509
MVP	JUN	0.5060352	-0.5994051	1.1054403	0.509
KIF13A	TEC	0.40670556	-0.6264632	1.03316876	0.509
IPO11	ABCA1	-0.6168594	0.6731664	1.2900258	0.508
KARS	VAV2	0.7770853	-0.4971271	1.2742124	0.508
ESRRG	SSFA2	-0.58733183	0.64258385	1.22991568	0.508
PFDN4	USP3	0.60700786	-0.56634647	1.17335433	0.508
ZNF385D	ZNF148	-0.52181053	0.6176165	1.13942703	0.508
HDAC1	CASP6	-0.6237511	0.46194217	1.08569327	0.508
PCNA	CDKN3	0.8788936	-0.28739098	1.16628458	0.507
XRCC5	WBP4	-0.39283657	0.67784417	1.07068074	0.507
XRCC5	KANK1	-0.69177175	0.3609746	1.05274635	0.507
GNAQ	TRIM2	0.41882876	-0.62769246	1.04652122	0.507
MMS19	JDP2	0.42286512	-0.6076294	1.03049452	0.507
DCUN1D5	THRB	-0.2646342	0.7620404	1.0266746	0.506
PRMT8	FBN1	-0.75142187	0.27307254	1.02449441	0.506
ESRRG	CYBRD1	-0.58438814	0.6679339	1.25232204	0.505
B2M	MED14	-0.4739867	0.6733753	1.147362	0.505
OLMALINC	SERPINE2	-0.5921834	0.4390673	1.0312507	0.505
KLF12	XPO5	0.2296844	-0.7757916	1.005476	0.505
RAB35	AHI1	0.6014385	-0.58553165	1.18697015	0.504
STAT3	CLSTN1	-0.20614776	0.80432075	1.01046851	0.504
DCN	LTBP4	0.9358703	-0.25856522	1.19443552	0.503
DIAPH1	SOX4	-0.622739	0.5522068	1.1749458	0.503
PELP1	PHF8	0.6664695	-0.5032513	1.1697208	0.503
IPO11	CHD2	-0.49395874	0.5920686	1.08602734	0.503
YARS	AUP1	-0.22810902	0.7722622	1.00037122	0.503
MED14	HLA-C	-0.46427476	0.7101785	1.17445326	0.502
PSMA4	EBF1	-0.7309841	0.40315396	1.13413806	0.502
SMC4	FOXM1	0.8803084	-0.24588135	1.12618975	0.502
XRCC5	IGF2R	-0.62491566	0.44563824	1.0705539	0.502
SNRPB2	DPP3	0.7793292	-0.2426178	1.021947	0.502
CCND2	PPP2R4	0.78921294	-0.5342362	1.32344914	0.501
KCNAB1	COL1A1	0.5467718	-0.60329264	1.15006444	0.501
KIF11	NUSAP1	0.95173293	-0.11290682	1.06463975	0.501
UBE2B	MAP4	0.56445485	-0.45064545	1.0151003	0.501
TK1	DDAH2	-0.7263438	0.52926314	1.25560694	0.5
COL4A1	VLDLR	-0.2917448	0.7305824	1.0223272	0.5
