# Supplementary material for: Identification of key regulatory genes connected to NF-κB family of proteins in visceral adipose tissues using gene expression and weighted protein interaction network
Source: PLoS One. 2019 Apr 23;14(4):e0214337. doi: 10.1371/journal.pone.0214337 (PMC6478283; doi:10.1371/journal.pone.0214337)
Supplement: S7 Table — (PDF) [file pone.0214337.s007.pdf]

S7 Table: List of genes involved in obesity or obesity related disorders with their fold change and statistical significance. The bold colored gene represents the target genes of NF-κB proteins.

Gene	FC	P value	PubMed
ELOVL6	-3.57931	0.004858	17906635, 20228221, 19259639, 26628376
VEGFA	-1.54068	0.039188	27819131, 27819131, 23805408, 18713823, 22451920
JUNB	2.429319	0.013038	24925581, 24186979, 16306344, 24186979, 24411941, 22169755
NGFR	-1.53768	0.019127	26278479, 25896784, 24608438,
PGRMC2	1.573806	0.015074	28388727
PIK3R1	1.79652	0.012608	22698915, 27766312, 15331535, 28478612, 23991359, 17016694
PTPN11	1.66582	0.001426	15520383, 22431513
SPTBN1	1.69658	0.002139	29115457, 29145611
FOS	5.645199	0.005852	10435786, 28527681, 10856820
ABCA1	-1.50857	0.01755	21289254, 24443560, 29348118, 17135600, 26891315,
AHNAK	1.612503	0.013673	30154465, 26466345, 26987950
STAT1	-1.85297	0.026271	22969086, 23735217, 26972840
ABCG1	-2.17004	0.000164	26451289, 25249572, 22179025, 25249572
MITF	1.988386	0.000116	24789918, 24477476, 28380427
IRF1	-1.62929	0.032121	28416283, 22874466, 21187013
HSP90AA1	1.608236	0.007092	23261432, 24096869
YME1L1	-1.94133	0.019475	29579150,
JUND	1.999995	0.001439	24186979, 24411941, 22169755
SP3	-1.60374	0.027501	12378384, 18664368
PALLD	1.580293	0.016407	21602305, 24549139, 21602305
AGPAT5	-1.59866	0.004458	25651185, 16436371, 25415055
CD69	2.332444	0.003264	29888230, 27609769, 21911743, 29888230, 29560551, 22024641
DDX39A	-1.50852	0.001805	29441128
CALM1	1.72647	0.002641	28246289
NEDD4L	1.925482	0.001355	23549273, 28017963, 21154329, 20003179
PTX3	-2.57139	0.047881	24415446, 28770376, 28400677, 30105036, 24215445, 25467846, 26725766, 28440716
EZH2	-1.60725	0.020643	9122234, 26869351, 27151441
SORL1	2.453291	0.001113	27322061, 27832290
KLF4	2.919027	0.000531	29864940, 24928509, 21670502, 27881420, 30240435, 27777310
TXNIP	1.755448	0.028682	21119640, 26696635, 21508227, 24482226, 19875615, 29339473
ERG1	3.7059453	0.002368	23502673, 19229250, 29607419
TNC	-3.07138	0.003811	26603137, 22851489,27612200, 29449623
BCL2	1.615368	0.013141	23193206, 29100331, 29348885, 26900794, 28280903, 27416945, 28607631
COL1A1	-1.58716	0.014842	21775118, 20107860
VCAM1	-2.07178	0.009974	27853845, 19249917, 10523328, 28297678
JUN	3.955379	0.000236	23967297, 21182758, 26608096
TEAD4	-1.55101	0.009679	29666371, 21187013
HSPA1A	2.238211	0.00024	25720752, 25379403, 24599575, 25720752, 18223156
IGF1R	-1.52481	0.024148	29150385, 29430464, 26237614, 28402847, 28244645
TGM2	-1.98368	0.003705	26313919, 21900603
